# Visible-Light-Triggered and Rhodamine B‑Catalyzed Thiolation of Bromoalkynes: Accessing Alkynyl Thioethers

**DOI:** 10.1021/acs.joc.5c01295

**Published:** 2025-07-23

**Authors:** Rekha Bai, Yu-Ling Chiu, Indrajit Karmakar, Chin-Fa Lee

**Affiliations:** a Department of Chemistry, 34916 National Chung Hsing University, Taichung City, Taiwan 402, R.O.C.; b i-Center for Advanced Science and Technology (iCAST), 34916 National Chung Hsing University, Taichung City, Taiwan 402, R.O.C.; c Innovation and Development Center of Sustainable Agriculture (IDCSA), 34916 National Chung Hsing University, Taichung City, Taiwan 402, R.O.C.

## Abstract

A visible-light-mediated C­(sp)–S^II^ *cross*-coupling of 1-haloalkynes with aryl/alkyl thiols is reported. The reaction was catalyzed by Rhodamine B under purple LED irradiation in an open-air atmosphere. Rhodamine B is a basic xanthene dye that is easily degradable and highly soluble in water. A wide range of 1-haloalkynes efficiently coupled with aryl and alkyl thiols, obtained corresponding alkynyl thioethers in moderate to excellent yields. Metal-free, room temperature, easy operation, and broad substrate scope are the salient features of the established protocol.

## Graphical abstract

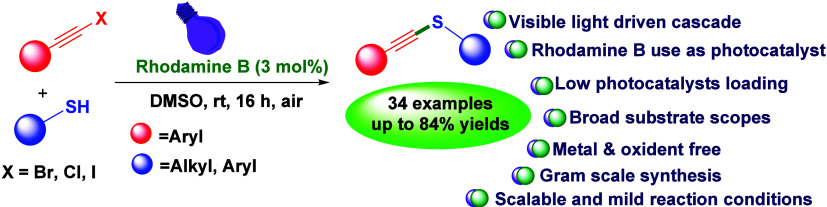

## Introduction

In modern organic synthesis, the visible-light-catalyzed strategies have gradually become one of the most powerful tools in organic synthetic chemistry.^1–4^ The availability, abundance, and safety of visible light makes the synthetic strategy green, operationally feasible, and eco-efficient. However, most organic compounds cannot directly absorb visible light, limiting their ability to undergo desired transformations.^5^ To address this challenge, organic chemists employ various photocatalysts with visible light, for example nanoparticles,^6^ transition metal complexes,^3c^ and organic dyes. Despite their advantages, both transition metal complexes and nanoparticles have some notable drawbacks such as nanoparticles, which often suffer from reduced crystallinity and a large band gap, leading to lower efficiency, while transition metal complex photocatalysts suffered from limited sustainability, high cost, low robustness, inherent harmfulness, and significant toxicity.^7^ As a result, organic dyes such as eosin Y, eosin B, Rhodamine 6G, and Rhodamine B (Rh B) are some of the well-known examples recognized as an excellent alternative of the nanoparticles and metal complex forms of photocatalysts.^7e^

Alkynyl thioethers, containing a sulfur atom directly bonded to a carbon–carbon triple bond, are crucial precursors for organic synthesis^8,9^ as they are present in biologically active moieties.^10^ They also serve as key intermediates for the production of sulfur-rich functional polymers.^9e,11^ Some examples of molecules having alkynyl thioether functionality are shown in Figure 1, which includes roles as pesticides **A**, MTase inhibitor candidate **B**, mGlu5 receptor antagonists **C**, HIV-1 reverse transcriptase inhibitors **D**, trichophytosis **E**, and antimicrobial agent precursor **F**.^12,13^ Due to the importance of alkynyl sulfides, significant efforts have been made to develop efficient and selective synthesis.

**Figure 1:**
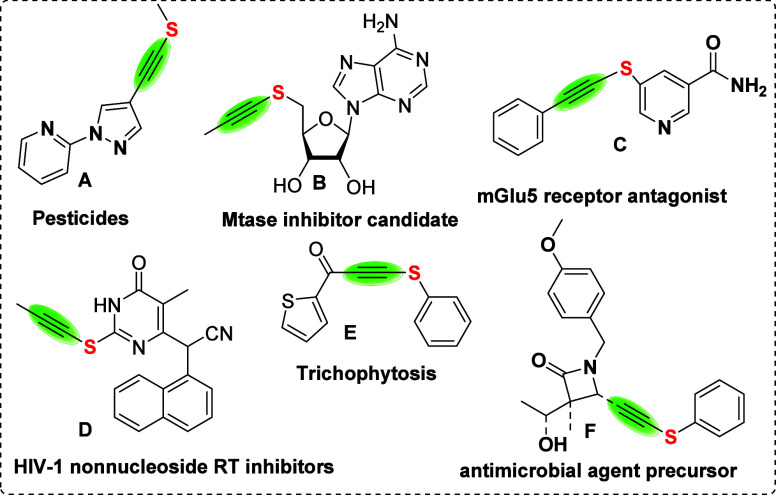
Some examples of bioactive alkynyl sulfides.

Although various synthetic methods have been developed for C­(sp^3^)–S and C­(sp^2^)–S bond formation,^14,15^ there are still challenges for the construction of C­(sp)–S bonds, predominantly for the achievement of C­(sp)–S^II^ bond formations. Traditional approaches have employed alkynyl halides or terminal alkynes with sulfur surrogates such as thiols, disulfides, and sulfenyl halides (Scheme 1a).^16^ Later, metal-free strategies were also developed for the synthesis of alkynyl sulfides. For example, Pan and co-workers^17,17a^ have developed a C­(sp)–S^II^ coupling of 1,1-dibromoalkenes and thiols using 4.0 equiv. Cs_2_CO_3_ as a base under nitrogen atmosphere (Scheme 1b). Wilden’s group introduced an alternative approach for synthesizing alkynyl chalcogenides through the displacement at the sp center.^17b^ Later, Reeves and co-workers^17c^ have described a methodology for the preparations of alkynyl sulfides using Grignard reagent and Bunte salts as starting precursors and tetrahydrofuran as solvent (Scheme 1c). Photochemical strategies have also been extensively employed in construction of C–S bonds.^18^ However, reported photoinduced C–S coupling reactions are predominantly limited to the formation of C­(sp^2^/sp^3^)–S bonds.^19,20^ A few reports are available for the synthesis of alkynyl thioethers by using visible light. For example, Collins established a photochemical C­(sp)–S^II^ cross-coupling reaction between bromoalkynes and thiols to synthesize alkynyl thioethers, employing a dual-catalytic system comprising a photocatalyst and NiCl_2_.dme (Scheme 1d).^21,21a^ A visible light-induced and copper-catalyzed C­(sp)–S coupling between the dimer of 2-amino thiol and terminal alkynes was developed by Anandhan and Reddy (Scheme 1e).^21b^ In 2021, an amino-assisted and visible-light-catalyzed Csp–S *cross*-coupling reaction of bromoalkynes with 2,2′-diaminodiaryldisulfides to excess alkynyl sulfides has been developed by Wang and co-workers^21c^ (Scheme 1f). These methods have some associated drawbacks and limitations such as low selectivity to the targeted products, use of expensive metal photocatalysts or additives, limited substrate scope, and high temperature to hasten the reaction. Herein, we report a visible-light-mediated C­(sp)–S^II^ *cross*-coupling reaction between 1-bromoalkynes and alkyl/aryl thiols to synthesize alkynyl sulfides under an open-air atmosphere, utilizing the commercially available and easily degradable photocatalyst Rhodamine B (Scheme 1g).^22–24^

**Scheme 1:**
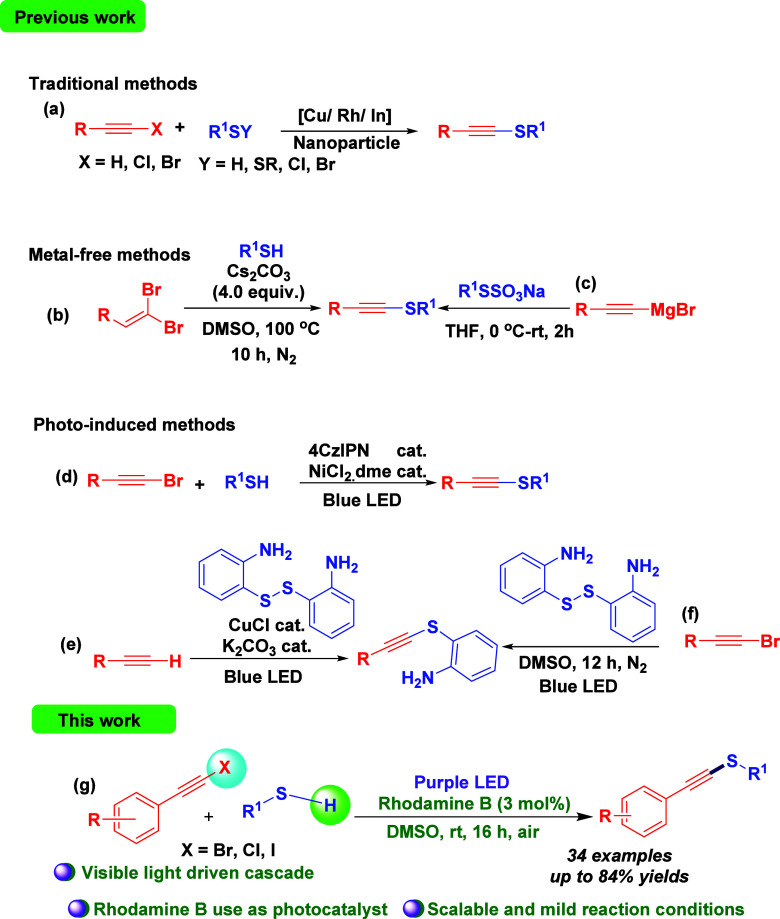
Synthetic Methodologies of Alkynyl Thioethers

## Results and Discussion

We began our investigation by choosing (bromoethynyl)­benzene (**1a**) and 3-methoxybenene thiol (**2a**) as model substrates. Initially, we reacted (bromoethynyl)­benzene (**1a**, 0.5 mmol) with 3-methoxy benzene thiol (**2a**, 0.7 mmol) in DMSO (2 mL) using 3 mol % of eosin Y as a photocatalyst under purple light-emitting-diode (LED) light for 16 h, and the expected product **3a** was obtained in 20% yield (Table 1, entry 1). Motivated by this finding, we then performed other experiments with varied reaction parameters, including the photocatalyst, light source, solvent, and reaction time (Table 1). Only trace amounts of the product **3a** was detected by GC-MS analysis when the reaction was carried out in the absence of photocatalyst or visible light (Table 1, entries 2 and 3), demonstrating that both light source and photocatalysts are essential for this C­(sp)–S^II^ coupling reaction. Interestingly, product **3a** was isolated in 82% yield when the reaction was performed in the presence of 3 mol % Rhodamine B as the photocatalyst (Table 1, entry 4). An 83% yield of **3a** was obtained when Rhodamine B increased to 5 mol % (Table 1, entry 5). Testing other photocatalysts including methylene blue, Rose Bengal, and Rhodamine 6G showed that these photocatalysts were less reactive than Rhodamine B to give the desired product **3a** in 50–60% yields (Table 1, entries 6–8). Subsequently, other light sources such as green LED, white LED, and blue LED were also investigated, but they could only give relatively lower yields ranging from 42 to 60% (Table 1, entries 9–11). Solvents were also tested; when DMF and ACN were used as the solvents, 62 and 60% of the product **3a** were isolated, respectively (Table 1, entries 12 and 13). No desired product was observed when H_2_O was used as the solvent (Table 1, entry 14). Other solvents such as THF and toluene were less effective than DMSO and could not provide better yield (Table 1, entries 15 and 16). Finally, some contrast experiments were also evaluated performing the reaction under O_2_ or N_2_ atmosphere and changing the reaction time did not afford higher yields of the product **3a** (Table 1, entries 17–19). To improve the efficiency of the photocatalytic system, we further investigated the effect of various bases such as K_2_CO_3_, Cs_2_CO_3_, and NEt_3_ (Table[Table tbl1], entries 20–22). When 0.5 mmol of K_2_CO_3_ or Cs_2_CO_3_ was employed, the alkynyl thioether **3a** was isolated in 84 and 83% yields, respectively (Table 1, entries 20 and 21). In contrast, the use of NEt_3_ resulted in 65% yield of **3a** (Table 1, entry 22). Additionally, when 1.0 mol % Rhodamine B was used as photocatalyst, only 25% of **3a** was isolated (Table 1, entry 23).

**Table 1:**
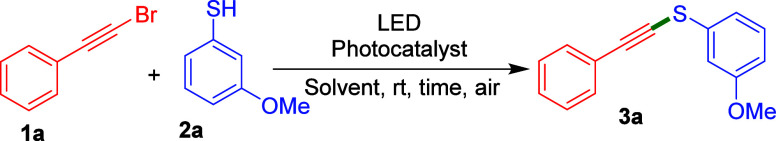
Optimization of Reaction Conditions^a^

**entry**	**LED**	**photocatalyst**	**solvent**	**yield % (3a)** ^b^
1	purple	eosin Y	DMSO	20
2	purple		DMSO	trace
3		eosin Y	DMSO	trace
**4**	**purple**	rhodamine B	**DMSO**	**82**
5^c^	purple	rhodamine B	DMSO	83
6	purple	methylene blue	DMSO	50
7	purple	Rose Bengal	DMSO	60
8	purple	rhodamine 6G	DMSO	52
9	green	rhodamine B	DMSO	60
10	white	rhodamine B	DMSO	55
11	blue	rhodamine B	DMSO	42
12	purple	rhodamine B	DMF	62
13	purple	rhodamine B	ACN	60
14	purple	rhodamine B	H_2_O	NR
15	purple	rhodamine B	THF	19
16	purple	rhodamine B	toluene	7
17^d^	purple	rhodamine B	DMSO	80
18^e^	purple	rhodamine B	DMSO	78
19^f^	purple	rhodamine B	DMSO	72
20^g^	purple	rhodamine B	DMSO	84
21^h^	purple	rhodamine B	DMSO	83
22^i^	purple	rhodamine B	DMSO	65
23^j^	purple	rhodamine B	DMSO	25

a Reaction conditions: (bromoethynyl)­benzene **1a** (0.5 mmol), 3-methoxybenzene thiol **2a** (0.7 mmol), photocatalyst (3–5 mol %), were stirred in solvent (2 mL) under visible light for 10–20 h in an open-air atmosphere.

b Isolated yields.

c Photocatalyst used 5 mol %.

d Under O_2_.

e Under N_2_.

f Yield after 10 h.

g 0.5 mmol K_2_CO_3_ used.

h 0.5 mmol Cs_2_CO_3_ used.

i 0.5 NEt_3_ used.

j 1.0 mol % photocatalyst used.

With optimized reaction conditions in hand, we then proceeded to explore the substrate scope of the visible light-induced C­(sp)–S^II^ *cross*-coupling reaction and results are abridged in Tables 2 and 3. Initially, the *S*-arylation and *S*-alkylation of a (haloethynyl)­benzene were investigated with a broad range of aryl, heteroaryl, and alkyl thiols under optimized reaction conditions (Table 2). First, 3-methoxybenzenethiol (**2a**) efficiently reacted with (bromoethynyl)­benzene, (chloroethynyl)­benzene, and (iodoethynyl)­benzene under optimized reaction conditions and delivered the desired product **3a** in 82, 84, and 60% yields, respectively. Benzene thiol (**2b**) efficiently reacted with (bromoethynyl)­benzene, yielding the desired product **3b** in 63% yield. Aryl thiols bearing electron-donating groups, such as 2-methoxybenzene thiol (**2c**), 4-methoxybenzene thiol (**2d**), and 4-methylbenzene thiol (**2e**), successfully achieved the desired alkynyl thioethers **3c**, **3d**, and **3e** in 71, 55, and 53% yields, respectively. Notably, aryl thiols with bulky and sterically hundred substituents at different positions, including 2-ethyl, 2-*iso*-propyl, and 4-*tert*-butly groups, were also efficiently coupled with (bromoethynyl)­benzene (**1a**), affording desired product **3f**–**3h** in 52–69% yields. Furthermore, disubstituted thiols, for example, 2,6-dimethylbenzene thiol, 2,4-dimethylbenzene thiol, and 3,4-dimethoxybenzene thiol, underwent successfully C–S coupling reaction with **1a**, producing the corresponding thioethers **3i**, **3j**, and **3k** in 66, 58, and 69% yields, respectively. Interestingly, 4-methoxybenzenethiol and 2,6-dimethylbenzene thiol were also compatible with both (chloroethynyl)­benzene and (iodoethynyl)­benzene, delivering the desired products **3d** and **3i** in 35–53% yields. Similarly, aryl thiols bearing an electron-withdrawing group at the *meta*-and *ortho*-positions such as 2-bromobenzene thiol and 3-chloro benzene thiol were also efficiently reacted with (bromoethynyl)­benzene, rendering the desired products **3l** and **3m** in 68 and 73%, respectively. Moreover, to our delight, the *S*-alkylation of (bromoethynyl)­benzene with alkyl thiols such as cyclohexanethiol (**2n**), dodecanethiol (**2o**), and benzyl mercaptan (**2p**) successfully yielded the corresponding thioethers **3n**, **3o**, and **3p** in 46, 48, and 78% yields, respectively. To further expand the substrate scope, naphthalene-2-thiol (**2q**), benzo­[*d*]­oxazole-2-thiol (**2r**), and 4-aminobenzenethiol (**2s**) reacted with (bromoethynyl)­benzene, (chloroethynyl)­benzene, and (iodoethynyl)­benzene under optimized reaction conditions, providing the desired alkenyl thioethers **3q**–**3s** in good yields. The structures of products **3a**–**s** were determined using ^1^H NMR, ^13^C NMR, ^19^F NMR, and HRMS data analyses.

**Table 2:**
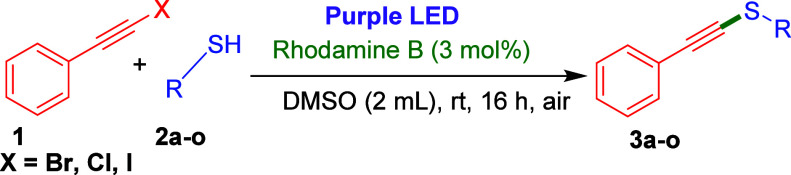

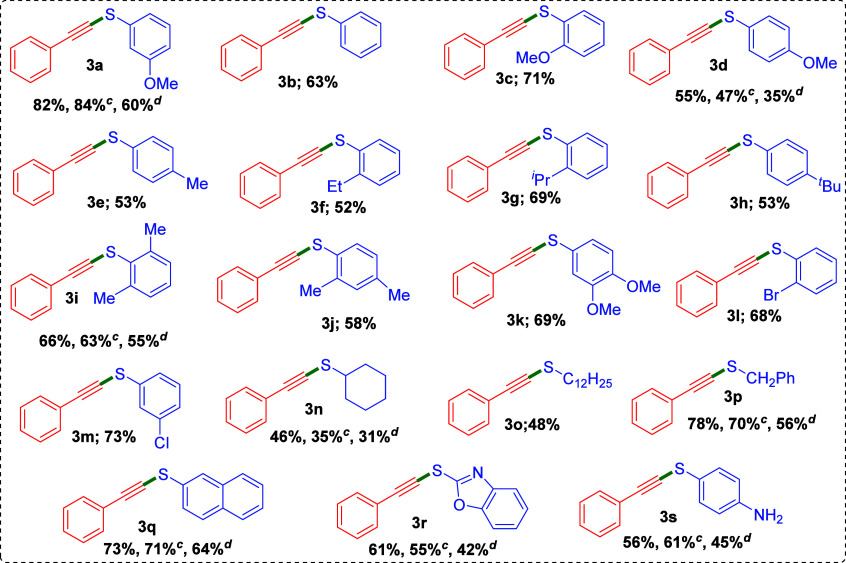
Substrate Scope of Various Thiols with (Bromoethynyl)­benzene^a^ ^,^ ^b^

a Reaction conditions: (bromoethynyl)­benzene **1a** (0.5 mmol), thiols **2a**–**o** (0.7 mmol), and rhodamine B (3 mol %) were stirred in DMSO (2 mL) under purple LED for 16 h under in an open-air atmosphere.

b Isolated yields.

c 0.5 mmol (chloroethynyl)­benzene used.

d 0.5 mmol (iodoethynyl)­benzene used.

**Table 3:**
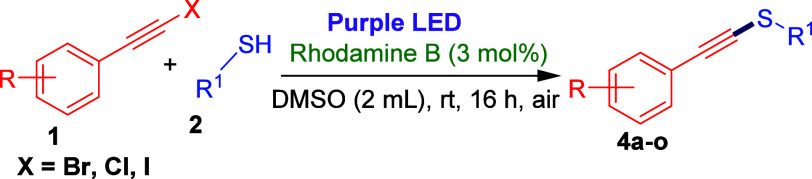

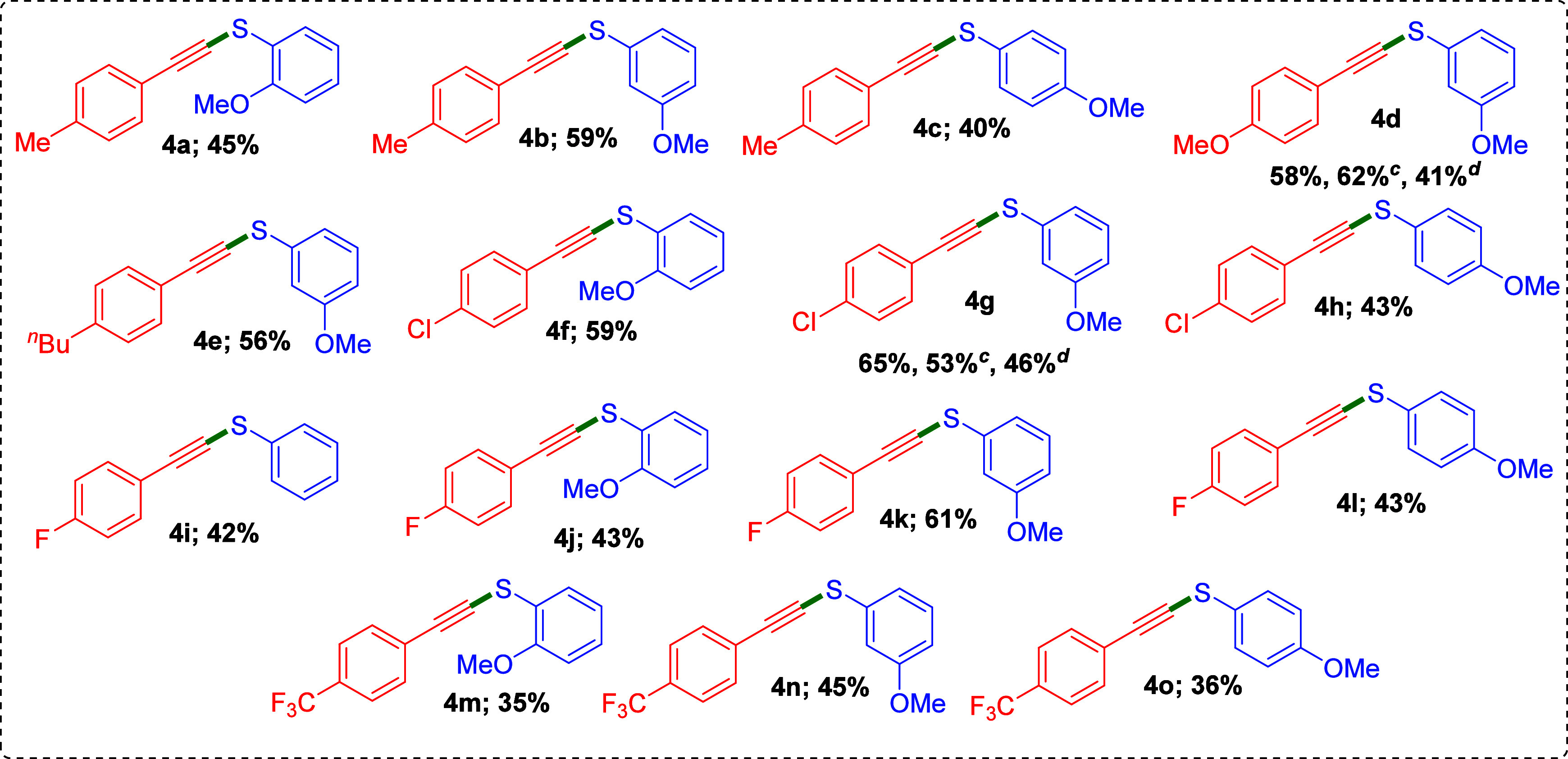
Substrate Scope of Various 1-Bromoalkynes with Aryl Thiols^a^ ^,^ ^b^

a Reaction conditions: 1-bromoalkynes **1a**–**g** (0.5 mmol), thiols **2** (0.7 mmol), and rhodamine B (3 mol %) were stirred in DMSO (2 mL) under purple LED for 16 h in an open-air atmosphere.

b Isolated yields.

c 0.5 mmol (chloroethynyl)­benzene used.

d 0.5 mmol (iodoethynyl)­benzene used.

After exploring the scope of thiols, we next investigated the C­(sp)–S^II^ *cross*-coupling reaction for 1-bromoalkynes with aryl thiols possessing different functional groups (Table 3). We first explored the 1-bromoalkynes bearing electron-donating groups at the *para* position of the phenyl ring, such as 4-methyl (**1b**), 4-methoxy (**1c**), and 4-butyl (**1d**), with aryl thiols. 1-(Bromoethynyl)-4-methylbenzene (**1b**) efficiently reacted with 2-methoxy, 3-methoxy, and 4-methoxybenzene thiols under optimized reaction conditions, yielding the corresponding alkynyl thioethers **4a**, **4b**, and **4c** in 45, 59, and 40% yields, respectively. Likewise, 1-(bromoethynyl)-4-methoxybenzene (**1c**) and 1-(bromoethynyl)-4-butylbenzene (**1d**) underwent coupling with 3-methoxybenzene thiol, rendering the desired products **4d** and **4e** in 58 and 56% yields, respectively. We then examined 1-bromoalkynes possessing electron-withdrawing groups, for example, 1-(bromoethynyl)-4-chlorobenzene (**1e**), 1-(bromoethynyl)-4-fluorobenzene (**1f**), and 1-(bromoethynyl)-4-(trifluoromethyl)­benzene (**1g**), under optimized reaction conditions. 1-(Bromoethynyl)-4-chlorobenzene (**1e**) successfully coupled with 2-methoxy, 3-methoxy, and 4-methoxybenzene thiols under optimized conditions, converting into corresponding products **4f**, **4g**, and, **4h** in 43–65% yields. Similarly, 1-(bromoethynyl)-4-fluorobenzene (**1f**) and 1-(bromoethynyl)-4-(trifluoromethyl)­benzene (**1g**) reacted with thiophenol, 2-methoxy, 3-methoxy, and 4-methoxybenzene thiols, yielding the desired products **4i**–**4o** in 36–61% yields. Interestingly, 3-methoxybenzenethiol (**2a**) also efficiently reacted with 1-(choloroethynyl)-4-methoxybenzene, 1-(iodoethynyl)-4-methoxybenzene, 1-(chloroethynyl)-4-chlorobenzene, and 1-(iodoethynyl)-4-chlorobenzene under optimized reaction conditions, delivering the desired products **4d** and **4g** in 41–62% yields. The structure of products **4a**–**o** were determined using ^1^H NMR, ^13^C NMR, ^19^F NMR, and HRMS data analyses.

To demonstrate the practical applicability of the visible-light-triggered photocatalytic thiolation reaction, a large-scale reaction was performed (Scheme 2). The reaction between (bromoethynyl)­benzene (**1a**; 5.0 mmol; 0.910 g) and 3-methoxybenzene thiol (**2a**; 7.0 mmol; 0.980 g) was carried out under optimized reaction conditions, yielding 79% (0.955 g) of desired product **3a**. This yield is comparable to that obtained on a 0.5 mmol scale, confirming that this catalytic system is easy to scale up.

**Scheme 2:**
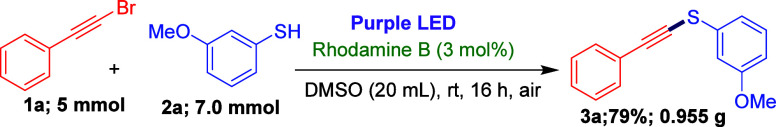
Large-Scale Synthesis of **3a**

To probe the reaction mechanism, some control experiments were conducted (Scheme 3). First, a reaction between **1a** and 3-methoxy diphenyl disulfide (**5a**) was performed under optimized reaction conditions, resulting in that no target product **3a** was obtained (Scheme 3a). Next, a radical trapping experiment was performed by adding 2.0 equiv of 2,2,6,6-tetramethylpiperidin-1-oxyl (**TEMPO**) to the standard reaction, resulting in that a trace amount of desired product **3a** and thiol/TEMPO adduct was detected in HRMS. Similarly, a reaction between **1a**, **2a**, and 2.0 equiv of **BHT** reacted under optimized reaction condition, with no detection of the desired product **3a** and thiol/BHT adduct in HRMS (Scheme 3b,c). These findings indicate that the reaction might involve the radical intermediates. Additionally, we have conducted the fluorescence quenching (Stern–Volmer) experiments to investigate the interaction between the excited state of Rh B and starting precursors. The results proved that an energy transfer process occurs between rhodamine B and 3-methoxybenzenethiol (**2a**) under visible-light irradiation (Figure 2).

**Scheme 3:**
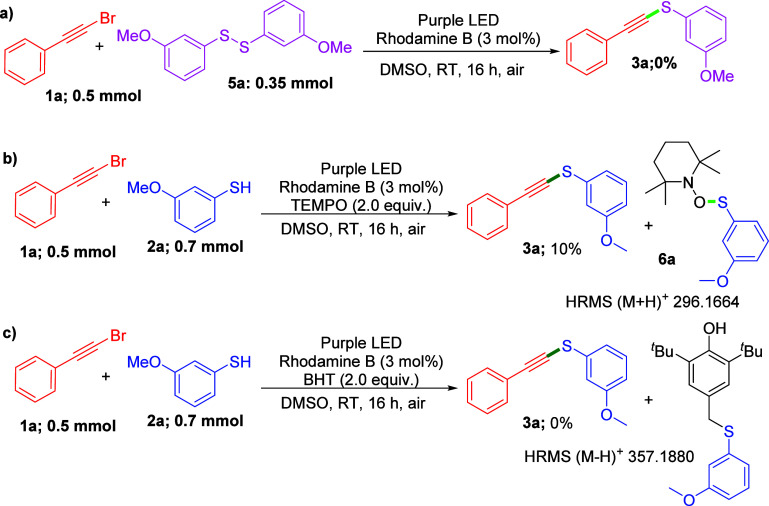
Control Experiments

**Figure 2:**
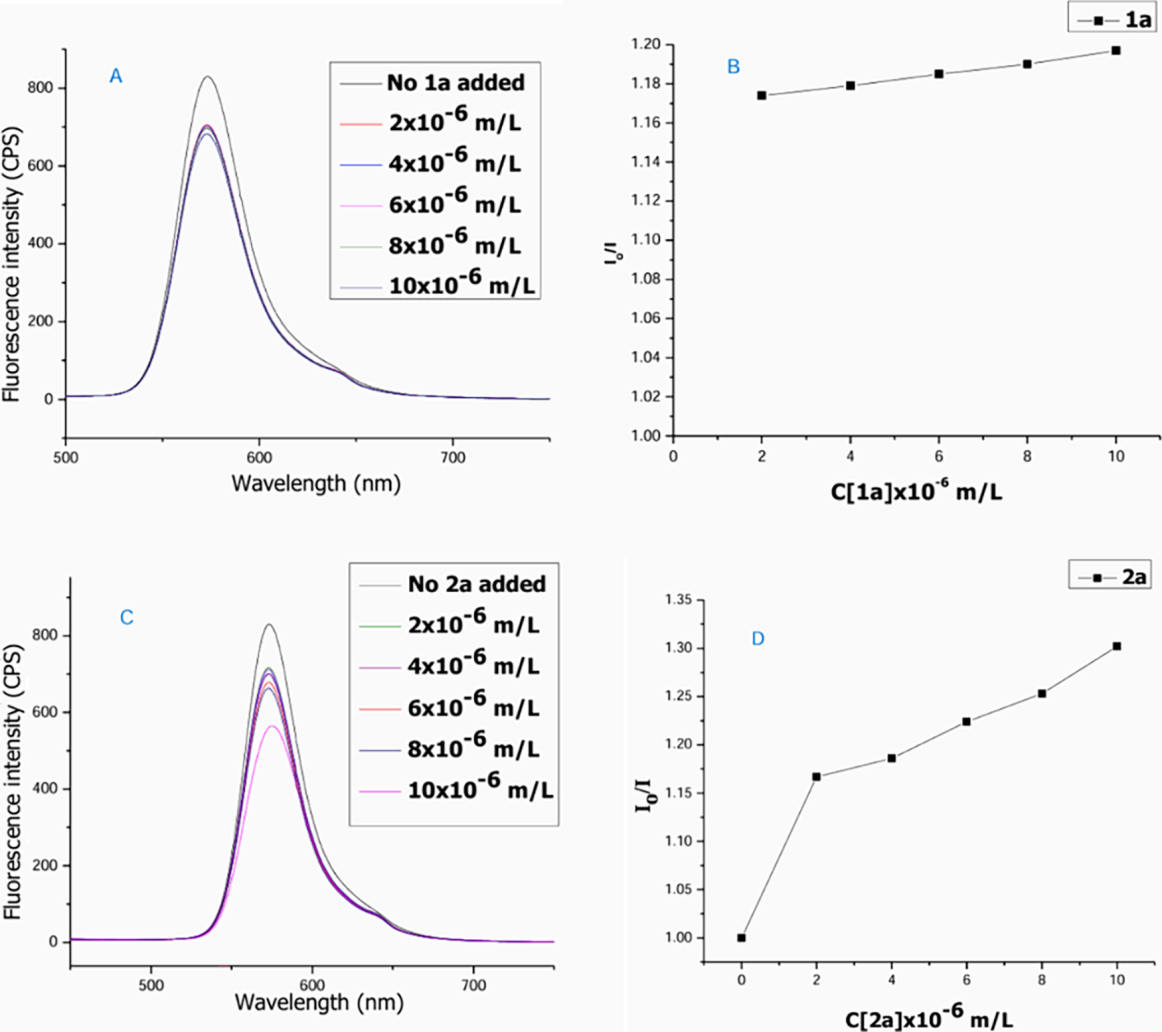
Stern–Volmer quenching experiments.

Based on control experiments and previously reported literature^15c,15e^ a plausible reaction mechanism is proposed (Scheme 4). Initially, in the presence of purple LED, Rhodamine B (Rh B) is photoexcited to Rh B*, which undergoes a single electron transfer (SET) electron from 3-methoxybenzene thiol (**2a**), affording Rh B^•–^ and a 3-methoxybenzenethiyl radical intermediate **I**. This thiyl radical intermediate **I** then reacts with (bromoethynyl)­benzene (**1a**) and converts into another intermediate **II**. This intermediate subsequently undergoes dehalogenation to afford desired product **3a** along with a bromine radical. Bromine radical reduced to bromide anions and regenerated Rh B. Finally, the bromide anion reacted with H^+^, leading to the generation of hydrogen bromide.

**Scheme 4:**
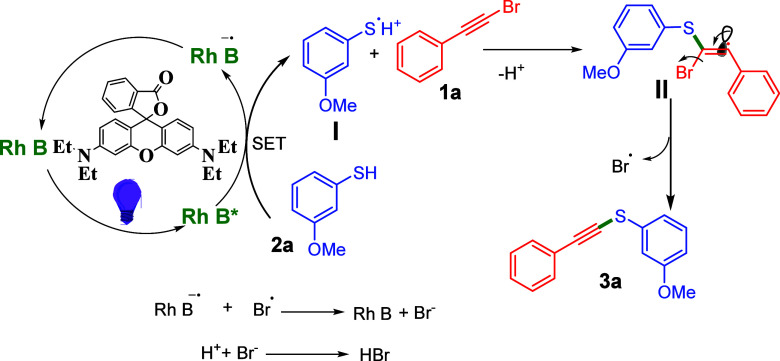
Plausible Reaction Mechanism

## Conclusions

We have established a simple and efficient method for the synthesis of alkynyl thioether from 1-haloalkynes and aryl/alkyl thiols. Rhodamine B catalyzes this transformation under purple light-emitting diode (LED) in the absence of an inert gas atmosphere. Rhodamine B, a xanthene dye, which is highly soluble in water and easily degradable, was found to be an excellent photocatalyst for this transformation. The established strategy was used to develop a broad substrate scope, in which various 1-haloalkynes efficiently coupled with aryl and alkyl thiols to produce alkynyl thioethers in 36–84% yields.

## Experimental Section

### General Information

All of the solvents used in this work were distilled and dried before use, following standard procedures. The instrument for photocatalysis is MDQL-CB108 (Diligent Vision LED-DB-6021B-AU) with no filters (MORITEX Corporation, Japan). The reaction-performed vessel is a Schlenk borosilicate tube. The distance from the light source to the irradiation Schlenk tube is ∼20–30 mm. Reactions were performed under an atmosphere of open air, O_2_, N_2_, or Ar gas with magnetic stirring. Reactions were monitored by thin-layer chromatography (TLC). TLC was performed using E. Merck precoated silica plates (60F-254) with a 0.25 mm thickness and visualized using short-wave UV light. Purifications were performed using flash column chromatography with 60–120-mesh silica gel as the stationary phase and a gradient of ethyl acetate in hexanes as the mobile phase. Known compounds were characterized by comparing their ^1^H NMR and ^13^C NMR spectra to the previously reported data. New compounds were characterized by ^1^H NMR, ^13^C NMR, and HRMS. The copies of ^1^H NMR, ^13^C NMR, and ^19^F NMR spectra are included at the end of the Supporting Information. NMR spectra were recorded in chloroform-*d* or dmso-*d*_*6*_ with tetramethylsilane as internal standard on a JEOL-400 MHz or Agilent-400 MHz NMR instruments. ^1^H NMR chemical shifts are reported in ppm (δ) relative to internal standard TMS (δ 0.00 ppm). ^13^C NMR chemical shifts are reported in ppm with respect to solvent resonance as the internal standard (CDCl_3_ at 77.10 ppm). ^1^H NMR data are reported as given here: chemical shift [multiplicity [singlet (s), doublet (d), triplet (t), quartet (q), pentet (p), multiplet (m), and broad singlet (br s), coupling constant (Hz), and integration]]. GC-MS analyses were carried out on an Agilent Technologies 5977A GC equipped with Agilent 7890B MS. High-resolution mass spectra (HRMS) were recorded on a JEOL JMS-HX 110 quadrupole type spectrometer provided by National Chung Hsing University. Starting materials **1a**–**e** were synthesized by reported literature procedures,^22a^ and benzene thiols and photocatalysts are commercially purchased and used without purification.

### Safety Statement

Caution! All reactions involving thiols, brominated compounds, and organic solvents must be conducted in a well-ventilated fume hood and wear appropriate personal protective equipment, to ensure safe handling of all reagents and byproducts. After reaction, hydrogen bromide forms as a byproduct, hence direct contact should be avoided, as it is corrosive and harmful. Use appropriate light shielding or protective covers for LED light sources to prevent eye damage and skin irritation. Never look directly into the light beam.

#### General Procedure for Synthesis of Compounds **3** and **4**

1-Bromoalkynes (0.5 mmol) and thiols (0.7 mmol) were added to a reaction vessel along with Rhodamine B (3 mol %) and DMSO (2 mL). The resulting mixture was stirred under purple LED light in open air for 16 h. Reaction progress was monitored by TLC. Upon completion, 20 mL of water and 20 mL of ethyl acetate were added. The organic layer was collected and concentrated by solvent removal. The resulting crude residue was purified using silica gel flash column chromatography (EA/hexanes) to obtained desired products **3** and **4**.

#### (3-Methoxyphenyl)­(phenylethynyl)­sulfane (**3a**)^22b^

Following the general procedure, (bromoethynyl)­benzene (**1a**, 0.5 mmol, 91 mg), 3-methoxythiophenol (**2a**, 0.7 mmol, 98 mg), Rhodamine B (3 mol %, 7.0 mg) were used and then purified by column chromatography (SiO_2_, hexanes) to provide **3a** as yellow liquid; yield 99.0 mg, 82%. ^1^H NMR (400 MHz, CDCl_3_): δ 7.52–7.49 (m, 2H), 7.36–7.33 (m, 3H), 7.28–7.24 (m, 1H), 7.07–7.04 (m, 2H), 6.79–6.76 (m, 1H), 3.81 (s, 3H); ^13^C­{H} NMR (100 MHz, CDCl_3_): δ 160.3, 134.2, 131.7, 130.1, 128.7, 128.5, 122.9, 118.5, 112.5, 111.6, 98.3, 75.3, 55.4.

#### Phenyl­(phenylethynyl)­sulfane (**3b**)^22c^

Following the general procedure, (bromoethynyl)­benzene (**1a**, 0.5 mmol, 91 mg), thiophenol (**2b**, 0.7 mmol, 77 mg), and Rhodamine B (3 mol %, 7.0 mg) were used and then purified by column chromatography (SiO_2_, hexanes) to provide **3b** as a colorless liquid; yield 66 mg, 63%. ^1^H NMR (400 MHz, CDCl_3_): δ 7.55–7.48 (m, 4H), 7.39–7.33 (m, 5H), 7.27–7.21 (m, 1H); ^13^C­{H} NMR (100 MHz, CDCl_3_): δ 133.0, 131.8, 129.3, 128.7, 128.4, 126.6, 126.2, 122.9, 98.0, 75.5.

#### (2-Methoxyphenyl)­(phenylethynyl)­sulfane (**3c**)

Following the general procedure, (bromoethynyl)­benzene (**1a**, 0.5 mmol, 91 mg), 2-methoxythiophenol (**2c**, 0.7 mmol, 98 mg), and Rhodamine B (3 mol %, 7.0 mg) were used and then purified by column chromatography (SiO_2_, hexanes) to provide **3c** as yellow liquid; yield 85.0 mg, 71%. ^1^H NMR (400 MHz, CDCl_3_): δ 7.67 (dd, *J* = 7.6 and 2.0 Hz, 1H), 7.58–7.52 (m, 2H), 7.36–7.34 (m, 3H), 7.25–7.19 (m, 1H), 7.05–7.01 (m, 1H), 6.85 (d, *J* = 8.0 Hz, 1H), 3.89 (s, 3H); ^13^C­{H} NMR (100 MHz, CDCl_3_): δ 155.2, 131.8, 128.6, 128.4, 127.3, 126.5, 123.0, 121.7, 121.6, 110.4, 98.5, 75.3, 55.9. HRMS (EI) calcd for C_15_H_12_OS [M]^+^ 240.0609, found 240.0617.

#### (4-Methoxyphenyl)­(phenylethynyl)­sulfane (**3d**)^22c^

Following the general procedure, (bromoethynyl)­benzene (**1a**, 0.5 mmol, 91 mg), 4-methoxythiophenol (**2d**, 0.7 mmol, 98 mg), and Rhodamine B (3 mol %, 7.0 mg) were used and then purified by column chromatography (SiO_2_, hexanes) to provide **3d** as yellow liquid; yield 66.0 mg, 55%. ^1^H NMR (400 MHz, CDCl_3_): δ 7.48–7.42 (m, 4H), 7.32–7.31 (m, 3H), 6.92–6.88 (m, 2H), 3.79 (s, 3H); ^13^C­{H} NMR (100 MHz, dmso*-d*_*6*_) δ 161.4, 131.5, 129.2, 128.9, 128.8, 122.6, 121.5, 115.6, 96.1, 55.4.

#### (Phenylethynyl)­(*p*-tolyl)­sulfane (**3e**)^22c^

Following the general procedure, (bromoethynyl)­benzene (**1a**, 0.5 mmol, 91 mg), 4-methylthiophenol (**2e**, 0.7 mmol, 87 mg), and Rhodamine B (3 mol %, 7.0 mg) were used and then purified by column chromatography (SiO_2_, hexanes) to provide **3e** as yellow solid; M.P. = 40–42 °C; yield 60 mg, 53%. ^1^H NMR (400 MHz, CDCl_3_): δ 7.51–7.48 (m, 2H), 7.38 (dd, *J* = 6.4 and 2.0 Hz, 2H), 7.35–7.33 (m, 3H), 7.16 (d, *J* = 8.0 Hz, 2H), 2.34 (s, 3H); ^13^C­{H} NMR (100 MHz, CDCl_3_): δ 136.7, 131.8, 130.1, 128.7, 128.6, 128.4, 126.6, 123.1, 97.3, 76.1, 21.1.

#### (2-Ethylphenyl)­(phenylethynyl)­sulfane (**3f**)

Following the general procedure, (bromoethynyl)­benzene (**1a**, 0.5 mmol, 91 mg), 2-ethylthiophenol (**2f**, 0.7 mmol, 98 mg), and Rhodamine B (3 mol %, 7 mg) were used and then purified by column chromatography (SiO_2_, hexanes) to provide **3f** as a yellow liquid; yield 62 mg, 52%. ^1^H NMR (400 MHz, CDCl_3_): δ 7.73 (d, *J* = 8.4 Hz, 1H), 7.52–7.50 (m, 2H), 7.34–7.32 (m, 3H), 7.24–7.14 (m, 3H), 2.71 (q, *J* = 7.6 Hz, 2H), 1.27 (t, *J* = 7.6 Hz, 3H); ^13^C­{H} NMR (100 MHz, CDCl_3_); δ 141.1, 131.8, 131.4, 128.6, 128.5, 127.1, 126.9, 123.1, 97.7, 75.8, 26.4, 14.2. HRMS (EI) calcd for C_16_H_14_S [M]^+^ 238.0816, found 238.0806.

#### (2-*iso-*Propylphenyl)­(phenylethynyl)­sulfane (**3g**)

Following the general procedure, (bromoethynyl)­benzene (**1a**, 0.5 mmol, 91 mg), 2-*iso*-propyl benzenethiol (**2g**, 0.7 mmol, 107 mg), and Rhodamine B (3 mol %, 7 mg) were used and then purified by column chromatography (SiO_2_, hexanes) to provide **3g** as a yellow liquid; yield 87 mg, 69%. ^1^H NMR (400 MHz, CDCl_3_): δ 7.77–7.32 (m, 1H), 7.53–7.47 (m, 2H), 7.35–7.33 (m, 3H), 7.28–7.22 (m, 3H), 3.27–3.17 (m, 1H), 1.28 (d, *J* = 7.6 Hz, 6H); ^13^C­{H} NMR (100 MHz, CDCl_3_): δ 145.9, 131.8, 130.9, 128.6, 128.5, 127.3, 127.1, 127.0, 125.6, 123.1, 97.7, 76.1, 30.3, 23.1. HRMS (EI) calcd for C_17_H_16_S [M]^+^ 252.0973, found 252.0977.

#### (4-(*tert*-Butyl)­phenyl)­(phenylethynyl)­sulfane (**3h**)^22d^

Following the general procedure, (bromoethynyl)­benzene (**1a**, 0.5 mmol, 91 mg), 4-(*tert-*butyl) benzenethiol (**2h**, 0.7 mmol, 117 mg), and Rhodamine B (3 mol %, 7 mg) were used and then purified by column chromatography (SiO_2_, hexanes) to provide **3h** as a yellow liquid; yield 71 mg, 53%. ^1^H NMR (400 MHz, CDCl_3_): δ 7.50–7.48 (m, 2H), 7.44–7.37 (m, 4H), 7.34–7.31 (m, 3H), 1.31 (s, 9H); ^13^C­{H} NMR (100 MHz, CDCl_3_): δ 150.0, 131.7, 129.3, 128.6, 128.4, 126.48, 126.42, 123.1, 97.3, 76.1, 34.6, 31.3.

#### (2,6-Dimethylphenyl)­(phenylethynyl)­sulfane (**3i**)

Following the general procedure, (bromoethynyl)­benzene (**1a**, 0.5 mmol, 91 mg), 2,6-dimethylbenzenethiol (**2i**, 0.7 mmol, 97 mg), and Rhodamine B (3 mol %, 7 mg) were used and then purified by column chromatography (SiO_2_, hexanes) to provide **3i** as a yellow liquid; yield 79 mg, 66%. ^1^H NMR (400 MHz, CDCl_3_): δ 7.37–7.34 (m, 2H), 7.26–7.23 (m, 3H), 7.19–7.11 (m, 3H), 2.63 (s, 6H); ^13^C­{H} NMR (100 MHz, CDCl_3_): δ 142.0, 131.6, 128.8, 129.2, 128.6, 128.3, 128.1, 123.5, 90.8, 78.6, 22.0. HRMS (EI) calcd for C_16_H_14_S [M]^+^ 238.0816, found 238.0826.

#### (2,4-Dimethylphenyl)­(phenylethynyl)­sulfane (**3j**)

Following the general procedure, (bromoethynyl)­benzene (**1a**, 0.5 mmol, 91 mg), 2,4-dimethylbenzenethiol (**2j**, 0.7 mmol, 97 mg), and Rhodamine B (3 mol %, 7 mg) were used and then purified by column chromatography (SiO_2_, hexanes) to provide **3j** as a yellow liquid; yield 69 mg, 58%. ^1^H NMR (400 MHz, CDCl_3_): δ 7.57 (d, *J* = 8.0 Hz, 1H), 7.50–7.48 (m, 2H), 7.34–7.32 (m, 3H), 7.04 (dd, *J* = 8.0 and 2.0 Hz, 1H), 7.00 (d, *J* = 2.0 Hz, 1H), 2.36 (s, 3H), 2.32 (s, 3H); ^13^C­{H} NMR (100 MHz, CDCl_3_): δ 136.8, 135.7, 131.7, 131.3, 128.5, 128.4, 128.3, 127.8, 127.5, 123.2, 96.8, 76.4, 20.9, 19.6. HRMS (EI) calcd for C_16_H_14_S [M]^+^ 238.0816, found 238.0823.

#### (3,4-Dimethoxyphenyl)­(phenylethynyl)­sulfane (**3k**)

Following the general procedure, (bromoethynyl)­benzene (**1a**, 0.5 mmol, 91 mg), 3,4-dimethoxybenzenethiol (**2k**, 0.7 mmol, 119 mg), and Rhodamine B (3 mol %, 7 mg) were used and then purified by column chromatography (SiO_2_, hexanes) to provide **3k** as a yellow liquid; yield 93 mg, 69%. ^1^H NMR (400 MHz, CDCl_3_): δ 7.48–7.46 (m, 2H), 7.34–7.31 (m, 3H), 7.08–7.05­(m, 2H), 6.86 (d, *J* = 8.0 Hz, 1H), 3.90 (s, 3H), 3.87 (s, 3H); ^13^C­{H} NMR (100 MHz, CDCl_3_): δ 149.7, 148.5, 131.5, 128.5, 128.4, 123.4, 123.0, 120.0, 112.0, 110.8, 96.8, 76.8, 56.1, 56.0. HRMS (EI) calcd for C_16_H_14_O_2_S [M]^+^ 270.0715, found 270.0721.

#### (2-Bromophenyl)­(phenylethynyl)­sulfane (**3l**)

Following the general procedure, (bromoethynyl)­benzene (**1a**, 0.5 mmol, 91 mg), 2-bromobenzenethiol (**2l**, 0.7 mmol, 132 mg), and Rhodamine B (3 mol %, 7 mg) were used and then purified by column chromatography (SiO_2_, hexanes) to provide **3l** as a colorless liquid; yield 98 mg, 68%. ^1^H NMR (400 MHz, CDCl_3_): δ 7.76 (dd, *J* = 8.0 and 2.0 Hz, 1H), 7.56–7.53 (m, 2H), 7.51 (dd, *J* = 8.0 and 1.6 Hz, 1H), 7.39–7.35 (m, 4H), 7.10 (dt, *J* = 7.6 and 1.6 Hz, 1H); ^13^C­{H} NMR (100 MHz, CDCl3): δ 134.8, 132.8, 132.0, 129.1, 128.5, 128.3, 127.5, 127.1, 122.6, 119.6, 99.7, 74.9. HRMS (EI) calcd for C_14_H_9_BrS [M]+ 287.9608, found 287.9613.

#### (3-Chlorophenyl)­(phenylethynyl)­sulfane (**3m**)^23a^

Following the general procedure, (bromoethynyl)­benzene (**1a**, 0.5 mmol, 91 mg), 3-chlorobenzenethiol (**2m**, 0.7 mmol, 101 mg), and Rhodamine B (3 mol %, 7 mg) were used and then purified by column chromatography (SiO_2_, hexanes) to provide **3m** as a colorless liquid; yield 89 mg, 73%. ^1^H NMR (400 MHz, CDCl_3_): δ 7.55–7.50 (m, 2H), 7.49–7.48 (m, 1H), 7.36–7.33 (m, 4H), 7.29 (dd, *J* = 7.6 and 0.4 Hz, 1H), 7.22–7.19 (m, 1H); ^13^C­{H} NMR (100 MHz, CDCl_3_): δ 135.3, 135.2, 131.9, 130.3, 129.2, 128.5, 126.7, 125.8, 124.4, 122.6, 99.1, 74.2.

#### Cyclohexyl­(phenylethynyl)­sulfane (**3n**)^23b^

Following the general procedure, (bromoethynyl)­benzene (**1a**, 0.5 mmol, 91 mg), cyclohexanethiol (**2n**, 0.7 mmol, 119 mg), and Rhodamine B (3 mol %, 7 mg) were used and then purified by column chromatography (SiO_2_, hexanes) to provide **3n** as a colorless liquid; yield 50 mg, 46%. ^1^H NMR (400 MHz, CDCl_3_); δ 7.43–7.39 (m, 2H), 7.32–7.26 (m, 3H), 3.94–2.96 (m, 1H), 2.13–2.19 (m, 2H), 1.85–1.79 (m, 2H), 1.66–1.52 (m, 2H), 1.39–1.20 (m, 4H); ^13^C NMR (100 MHz, CDCl_3_) δ 131.5, 128.3, 127.9, 123.8, 94.4, 78.6, 47.8, 33.1, 26.1, 25.5.

#### Dodecyl­(phenylethynyl)­sulfane (**3o**)^23c^

Following the general procedure, (bromoethynyl)­benzene (**1a**, 0.5 mmol, 91 mg), dodecane-1-thiol (**2o**, 0.7 mmol, 142 mg), and Rhodamine B (3 mol %, 7 mg) and were used and then purified by column chromatography (SiO_2_, hexanes) to provide **3o** as a colorless liquid; yield 73 mg, 48%. ^1^H NMR (400 MHz, CDCl_3_): δ 7.34–7.31 (m, 2H), 7.22–7.18 (m, 3H), 2.72 (t, *J* = 7.2 Hz, 2H), 1.76–1.68 (m, 2H), 1.39–1.34 (m, 2H), 1.24–1.18 (m, 16H), 0.81 (t, *J* = 6.8 Hz, 3H); ^13^C­{H} NMR (100 MHz, CDCl_3_); δ 131.5, 128.3, 127.9, 123.7, 92.9, 79.8, 35.9, 31.9, 29.73, 29.71, 29.7, 29.6, 29.43, 29.40, 29.2, 28.3, 22.8, 14.2.

#### Benzyl­(phenylethynyl)­sulfane (**3p**)^24a^

Following the general procedure, (bromoethynyl)­benzene (**1a**, 0.5 mmol, 91 mg), phenylmethanethiol (**2p**, 0.7 mmol, 87 mg), and Rhodamine B (3 mol %, 7 mg) were used and then purified by column chromatography (SiO_2_, hexanes) to provide **3p** as a colorless liquid; yield 88 mg, 78%. ^1^H NMR (400 MHz, CDCl_3_): δ 7.39–7.27 (m, 10H), 4.02 (s, 2H); ^13^C­{H} NMR (100 MHz, CDCl_3_); δ 136.6, 131.4, 129.2, 128.7, 128.3, 128.1, 127.9, 123.4, 94.7, 79.2, 40.5.

#### Naphthalen-2-yl­(phenylethynyl)­sulfane (**3q**)^24b^

Following the general procedure, (bromoethynyl)­benzene (**1a**, 0.5 mmol, 91 mg), naphthalene-2-thiol (**2q**, 0.7 mmol, 112 mg), and Rhodamine B (3 mol %, 7 mg) were used and then purified by column chromatography (SiO_2_, hexanes) to provide **3q** as a colorless liquid; yield 90 mg, 73%. ^1^H NMR (400 MHz, CDCl_3_): δ 7.86 (t, *J* = 2.0 Hz, 1H), 7.74–7.68 (m, 3H), 7.49–7.36 (m, 5H), 7.29–7.26 (m, 3H); ^13^C­{H} NMR (100 MHz, CDCl_3_): δ 133.8, 131.9, 130.3, 129.6, 129.1, 128.8, 128.5, 127.9, 127.2, 126.9, 126.1, 124.6, 124.2, 122.9, 98.1, 75.8.

#### 2-((Phenylethynyl)­thio)­benzo­[*d*]­oxazole (**3r**)

Following the general procedure, (bromoethynyl)­benzene (**1a**, 0.5 mmol, 91 mg), benzo­[*d*]­oxazole-2-thiol (**2r**, 0.7 mmol, 105 mg), and Rhodamine B (3 mol %, 7 mg) were used and then purified by column chromatography (SiO_2_, hexanes) to provide **3r** as a colorless liquid; yield 77 mg, 61%. ^1^H NMR (400 MHz, CDCl_3_): δ 7.58–7.55 (m, 3H), 7.33 (dd, *J* = 6.8 and 2.4 Hz, 1H), 7.28–7.25 (m, 3H), 7.24–7.21 (m, 2H); ^13^C­{H} NMR (100 MHz, CDCl_3_): δ 152.0, 141.6, 137.9, 136.0, 129.2, 128.7, 127.6, 124.7, 124.5, 119.3, 114.6, 110.1. HRMS (EI) calcd for C_15_H_9_NOS [M]+ 251.0405, found 251.0409.

#### 4-((Phenylethynyl)­thio)­aniline (**3s**)

Following the general procedure, (bromoethynyl)­benzene (**1a**, 0.5 mmol, 91 mg), 4-aminobenzenethiol (**2s**, 0.7 mmol, 88 mg), and Rhodamine B (3 mol %, 7.0 mg) were used and then purified by column chromatography (SiO_2_, hexanes) to provide **3s** as a yellow liquid; yield 63 mg, 56%. ^1^H NMR (400 MHz, CDCl_3_): δ 7.38–7.36 (m, 2H), 7.24–7.21 (m, 5H), 6.59 (dt, *J* = 9.6 and 3.2 Hz, 2H), 3.11 (brs, 2H); ^13^C­{H} NMR (100 MHz, CDCl_3_) δ 146.1, 134.8, 131.6, 129.7, 128.4, 128.3, 123.3, 119.7, 116.1, 95.5, 77.9. HRMS (EI) calcd for C_14_H_11_NS [M]+ 225.0612, found 225.0618.

#### (2-Methoxyphenyl)­(*p*-tolylethynyl)­sulfane (**4a**)

Following the general procedure, 1-(bromoethynyl)-4-methylbenzene (**1b**, 0.5 mmol, 98 mg), 2-methoxybenzenethiol (**2c**, 0.7 mmol, 98 mg), and Rhodamine B (3 mol %, 7 mg) were used and then purified by column chromatography (SiO_2_, hexanes) to provide **4a** as a colorless liquid; yield 58 mg, 45%. ^1^H NMR (400 MHz, CDCl_3_): δ 7.67 (d, *J* = 8.0 Hz, 1H), 7.43 (d, *J* = 8.0 Hz, 2H), 7.25–7.14 (m, 3H), 7.04–6.99 (m, 1H), 6.85 (d, *J* = 8.4 Hz, 1H), 3.89 (s, 3H), 2.37 (s, 3H); ^13^C­{H} NMR (100 MHz, CDCl_3_): δ 155.2, 138.9, 131.9, 129.2, 127.2, 126.6, 121.9, 121.7, 120.0, 10.4, 98.7, 74.3, 56.0, 21.6. HRMS (EI) calcd for C_16_H_14_OS [M]^+^ 254.0765, found 254.0766.

#### (3-Methoxyphenyl)­(*p*-tolylethynyl)­sulfane (**4b**)

Following the general procedure, 1-(bromoethynyl)-4-methylbenzene (**1b**, 0.5 mmol, 98 mg), 3-methoxybenzenethiol (**2a**, 0.7 mmol, 98 mg), and Rhodamine B (3 mol %, 7 mg) were used and then purified by column chromatography (SiO_2_, hexanes) to provide **4b** as a colorless liquid; yield 75 mg, 59%. ^1^H NMR (400 MHz, CDCl_3_): δ 7.40 (dd, *J* = 6.4 and 1.6 Hz, 2H), 7.27–7.23 (m, 1H), 7.14 (d, *J* = 8.0 Hz, 2H), 7.05–7.03 (m, 2H), 6.77–6.64 (m, 1H), 3.80 (s, 3H), 2.36 (s, 3H); ^13^C­{H} NMR (100 MHz, CDCl_3_): δ 160.2, 139.1, 134.5, 131.8, 130.1, 129.2, 119.8, 118.4, 112.4, 111.5, 98.6, 74.3, 55.4, 21.6.HRMS (EI) calcd for C_16_H_14_OS [M]^+^ 254.0765, found 254.0769.

#### (4-Methoxyphenyl)­(*p*-tolylethynyl)­sulfane (**4c**)

Following the general procedure, 1-(bromoethynyl)-4-methylbenzene (**1b**, 0.5 mmol, 98 mg), 4-methoxybenzenethiol (**2d**, 0.7 mmol, 98 mg), and Rhodamine B (3 mol %, 7 mg) were used and then purified by column chromatography (SiO_2_, hexanes) to provide **4c** as a yellow solid; M.P. 38–40 °C; yield 51 mg, 40%. ^1^H NMR (400 MHz, CDCl_3_): δ 7.42 (dd, *J* = 8.8 and 2.0 Hz, 2H), 7.37 (dd, *J* = 8.4 and 2.5 Hz, 2H), 7.12 (dd, *J* = 8.4 and 2.4 Hz, 2H), 6.90 (dd, *J* = 8.8 and 2.4 Hz, 2H), 3.80 (s, 3H), 2.35 (s, 3H); ^13^C­{H} NMR (100 MHz, CDCl_3_): δ 159.0, 138.8, 131.8, 129.2, 128.4, 123.4, 120.1, 115.1, 96.6, 76.0, 55.5, 21.6. HRMS (EI) calcd for C_16_H_14_OS [M]^+^ 254.0765, found 254.0764.

#### (3-Methoxyphenyl)­((4-methoxyphenyl)­ethynyl)­sulfane (**4d**)

Following the general procedure, 1-(bromoethynyl)-4-methoxybenzene (**1c**, 0.5 mmol, 106 mg), 3-methoxybenzenethiol (**2a**, 0.7 mmol, 98 mg), and Rhodamine B (3 mol %, 7 mg) were used and then purified by column chromatography (SiO_2_, hexanes) to provide **4d** as a yellow liquid; yield 79 mg, 58%.^1^H NMR (400 MHz, CDCl_3_): δ 7.47 (dt, *J* = 9.6 and 2.8 Hz, 2H), 7.26 (t, *J* = 4.8 Hz, 1H), 7.05 −7.03 (m, 2H), 6.87 (dt, *J* = 9.6 and 3.2 Hz, 2H), 6.77–6.74 (m, 1H), 3.83 (s, 3H), 3.81 (s, 3H); ^13^C­{H} NMR (100 MHz, CDCl_3_): δ 160.3, 160.2, 134.8, 133.8, 130.1, 118.4 115.0, 114.1, 112.3, 111.5, 98.4, 73.4, 55.4. HRMS (EI) calcd for C_16_H_14_O_2_S [M]^+^ 270.0715, found 270.0719.

#### ((4-Butylphenyl)­ethynyl)­(3-methoxyphenyl)­sulfane (**4**e)

Following the general procedure, 1-(bromoethynyl)-4-butylbenzene (**1d**, 0.5 mmol, 119 mg), 3-methoxybenzenethiol (**2a**, 0.7 mmol, 98 mg), and Rhodamine B (3 mol %, 7 mg) were used and then purified by column chromatography (SiO_2_, hexanes) to provide **4e** as a yellow liquid; yield 83 mg, 56%. ^1^H NMR (400 MHz, CDCl_3_): δ 7.42 (dd, *J* = 6.8 and 2.0 Hz, 2H), 7.27–7.23 (m, 1H), 7.15 (d, *J* = 8.0 Hz, 2H), 7.06–7.04 (m, 2H), 6.77–6.74 (m, 1H), 3.81 (s, 3H), 2.62 (t, *J* = 8.0 Hz, 2H), 1.63–1.55 (m, 2H), 1.39–1.31 (m, 2H), 0.93 (t, *J* = 7.6 Hz, 3H); ^13^C­{H} NMR (100 MHz, CDCl_3_): δ 160.3, 144.1, 134.6, 131.9, 130.1, 128.6, 120.1, 118.4, 112.4, 111.5, 98.6, 74.3, 55.4, 35.7, 33.4, 22.4, 13.9. HRMS (EI) calcd for C_19_H_20_OS [M]^+^ 296.1235, found 296.1241.

#### ((4-Chlorophenyl)­ethynyl)­(2-methoxyphenyl)­sulfane (**4f**)

Following the general procedure, 1-(bromoethynyl)-4-cholorobenzene (**1e**, 0.5 mmol, 103 mg), 2-methoxybenzenethiol (**2c**, 0.7 mmol, 98 mg), and Rhodamine B (3 mol %, 7 mg) were used and then purified by column chromatography (SiO_2_, hexanes) to provide **4f** as a white solid, M. P. 108–110 °C; yield 81 mg, 59%. ^1^H NMR (400 MHz, CDCl_3_) δ 7.63 (dd, *J* = 8.0 and 1.6 Hz, 1H), 7.44 (dt, *J* = 9.2 and 2.4 Hz, 2H), 7.31 (dt, *J* = 9.2 and 2.4 Hz, 2H), 7.23–7.19 (m, 1H), 7.02 (td, *J* = 7.6 and 1.2 Hz, 1H), 6.86 (dd, *J* = 8.4 and 1.2 Hz, 1H), 3.89 (s, 3H); ^13^C­{H} NMR (100 MHz, CDCl_3_): δ 155.3, 134.6, 132.9, 128.8, 127.5, 126.6, 121.7, 121.6, 121.2, 110.5, 97.3, 76.7, 55.9. HRMS (EI) calcd for C_15_H_11_ClOS [M]^+^ 274.0219, found 274.0226.

#### ((4-Chlorophenyl)­ethynyl)­(3-methoxyphenyl)­sulfane (**4g**)

Following the general procedure, 1-(bromoethynyl)-4-cholorobenzene (**1e**, 0.5 mmol, 103 mg), 3-methoxybenzenethiol (**2a**, 0.7 mmol, 98 mg), and Rhodamine B (3 mol %, 7 mg) were used and then purified by column chromatography (SiO_2_, hexanes) to provide **4g** as a yellow liquid; yield 91 mg, 65%. ^1^H NMR (400 MHz, CDCl_3_) δ 7.43 (dt, *J* = 9.6 and 2.4 Hz, 2H), 7.32 (dt, *J* = 9.6 and 2.4 Hz, 2H), 7.26 (d, *J* = 2.4 Hz, 1H), 7.06–7.03 (m, 2H), 6.79–6.77 (m, 1H), 3.82 (s, 3H); ^13^C­{H} NMR (100 MHz, DMSO-*d*_*6*_): δ 160.0, 133.9, 133.1, 132.6, 130.7, 128.9, 120.6, 118.0, 112.9, 111.2, 97.1, 76.3, 55.3. HRMS (EI) calcd for C_15_H_11_ClOS [M]^+^ 274.0219, found 274.0222.

#### ((4-Chlorophenyl)­ethynyl)­(4-methoxyphenyl)­sulfane (**4h**)

Following the general procedure, 1-(bromoethynyl)-4-cholorobenzene (**1e**, 0.5 mmol, 103 mg), 3-methoxybenzenethiol (**2a**, 0.7 mmol, 98 mg), and Rhodamine B (3 mol %, 7 mg) were used and then purified by column chromatography (SiO_2_, hexanes) to provide **4h** as a white solid, M. P. 58–61 °C; yield 59 mg, 43%. ^1^H NMR (400 MHz, CDCl_3_): δ 7.43–7.37 (m, 4H), 7.31–7.25 (m, 2H), 6.93–6.89 (m, 2H), 3.80 (s, 3H); ^13^C­{H} NMR (100 MHz, CDCl_3_): δ 159.2, 134.5, 132.9, 129.3, 128.8, 122.7, 121.6, 115.2, 95.0, 78.6, 55.5. HRMS (EI) calcd for C_15_H_11_ClOS [M]^+^ 274.0219, found 274.0223.

#### ((4-Fluorophenyl)­ethynyl)­(phenyl)­sulfane (**4i**)^22c^

Following the general procedure, using 1-(bromoethynyl)-4-fluorobenzene (**1f**, 0.5 mmol, 100 mg), benzenethiol (**2b**, 0.7 mmol, 77 mg), and Rhodamine B (3 mol %, 7 mg) were used and then purified by column chromatography (SiO_2_, hexanes) to provide **4i** as a yellow liquid; yield 48 mg, 42%. ^1^H NMR (400 MHz, CDCl_3_): δ 7.52–7.46 (m, 4H), 7.38–7.34 (m, 2H), 7.26–7.22 (m, 1H), 7.07–7.01­(m, 2H); ^13^C­{H} NMR (100 MHz, CDCl_3_): δ 162.8 (d, *J* = 250.0 Hz), 133.9 (d, *J* = 8.0 Hz), 132.8, 129.4, 126.7, 126.3, 119.1 (d, *J* = 4.0 Hz), 115.8 (d, *J* = 23 Hz), 96.8, 75.3; ^19^F NMR (376 MHz, CDCl_3_): δ −109.78.

#### ((4-Fluorophenyl)­ethynyl)­(2-methoxyphenyl)­sulfane (**4j**)

Following the general procedure, 1-(bromoethynyl)-4-fluorobenzene (**1f**, 0.5 mmol, 100 mg), 2-methoxybenzenethiol (**2c**, 0.7 mmol, 98 mg), and Rhodamine B (3 mol %, 7 mg) were used and then purified by column chromatography (SiO_2_, hexanes) to provide **4j** as a yellow solid, M. P. 68–69 °C; yield 56 mg, 43%. ^1^H NMR (400 MHz, CDCl_3_): δ 7.64 (dd, *J* = 7.6 Hz, 1H), 7.53–7.50 (m, 2H), 7.26–7.19 (m, 1H), 7.06–7.00 (m, 3H), 6.86 (dd, *J* = 8.0, 1.2 Hz, 1H), 3.90 (s, 3H); ^13^C­{H} NMR (100 MHz, CDCl_3_): δ 162.8 (d, *J* 249.0 Hz), 155.3, 133.9 (d, *J* = 9.0 Hz), 127.4, 126.6, 121.7, 121.5, 119.2 (d, *J* = 4.0 Hz), 115.8 (d, *J* = 22.0 Hz), 110.5, 97.3, 75.1, 56.0; ^19^F NMR (376 MHz, CDCl_3_) δ −90.05. HRMS (EI) calcd for C_15_H_11_FOS [M]^+^ 258.0515, found 258.0523.

#### ((4-Fluorophenyl)­ethynyl)­(3-methoxyphenyl)­sulfane (**4k**)

Following the general procedure, 1-(bromoethynyl)-4-fluorobenzene (**1f**, 0.5 mmol, 100 mg), 3-methoxybenzenethiol (**2a**, 0.7 mmol, 98 mg), and Rhodamine B (3 mol %, 7 mg) were used and then purified by column chromatography (SiO_2_, hexanes) to provide **4k** as a yellow liquid; yield 79 mg, 61%. ^1^H NMR (400 MHz, CDCl_3_): δ 7.52–7.48 (m, 2H), 7.28–7.25 (m, 1H), 7.06–7.02 (m, 4H), 6.79–6.76 (m, 1H), 3.81 (s, 3H); ^13^C­{H} NMR (100 MHz, CDCl_3_): δ 162.8 (d, *J* = 249.0 Hz), 134.31, 133.9 (d, *J* = 9.0 Hz), 131.2, 119.0 (d, *J* = 3.0 Hz), 118.5, 115.8 (d, *J* = 22.0 Hz), 112.4, 111.8, 97.1, 75.1, 55.4; ^19^F NMR (376 MHz, CDCl_3_) δ −109.72.HRMS (EI) calcd for C_15_H_11_FOS [M]^+^ 258.0515, found 258.0506.

#### ((4-Fluorophenyl)­ethynyl)­(4-methoxyphenyl)­sulfane (**4l**)

Following the general procedure, 1-(bromoethynyl)-4-fluorobenzene (**1f**, 0.5 mmol, 100 mg), 4-methoxybenzenethiol (**2d**, 0.7 mmol, 98 mg), and Rhodamine B (3 mol %, 7 mg) were used and then purified by column chromatography (SiO_2_, hexanes) to provide **4l** as a yellow liquid; yield 56 mg, 43%. ^1^H NMR (400 MHz, CDCl_3_): δ 7.48–7.41 (m, 4H), 7.05–6.99 (m, 2H), 6.93–6.90 (m, 2H), 3.81 (s, 3H); ^13^C­{H} NMR (100 MHz, CDCl_3_): δ 162.7 (d, *J* = 249.0 Hz), 159.2, 133.8 (d, *J* = 9.0 Hz), 129.1, 122.9, 119.2 (d, *J* = 3.0 Hz), 115.7 (d, *J* = 22.0 Hz), 115.2, 95.1, 55.5; ^19^F NMR (376 MHz, CDCl_3_) δ −109.93.HRMS (EI) calcd for C_15_H_11_FOS [M]^+^ 258.0515, found 258.0524.

#### (2-Methoxyphenyl)­((4-(trifluoromethyl)­phenyl)­ethynyl)­sulfane (**4m**)

Following the general procedure, 1-(bromoethynyl)-4-(trifluoromethyl)­benzene (**1g**, 0.5 mmol, 125 mg), 2-methoxybenzenethiol (**2c**, 0.7 mmol, 98 mg), and Rhodamine B (3 mol %, 7 mg) were used and then purified by column chromatography (SiO_2_, hexanes) to provide **4m** as a yellow solid, M. P. 53–54 °C; yield 54 mg, 35%. ^1^H NMR (400 MHz, CDCl_3_): δ 7.64 (dd, *J* = 8.0 and 2.0 Hz, 1H), 7.60 (s, 4H), 7.26–7.22 (m, 1H), 7.04 (td, *J* = 7.6 and 1.2 Hz, 1H), 6.88 (dd, *J* = 8.4 and 1.2 Hz, 1H), 3.91 (s, 3H); ^13^C­{H} NMR (100 MHz, CDCl_3_): δ 155.4, 131.6, 130.1 (d, *J* = 32.0 Hz), 127.7, 126.9, 126.7, 125.4 (q, *J* = 4.0 Hz), 122.6, 121.8, 120.8, 110.6, 97.1, 79.0, 56.0; ^19^F NMR (376 MHz, CDCl_3_) δ −62.69. HRMS (EI) calcd for C_16_H_11_F_3_OS [M]^+^ 308.0483, found 308.0485.

#### (3-Methoxyphenyl)­((4-(trifluoromethyl)­phenyl)­ethynyl)­sulfane (**4n**)

Following the general procedure, 1-(bromoethynyl)-4-(trifluoromethyl)­benzene (**1g**, 0.5 mmol, 125 mg), 3-methoxybenzenethiol (**2a**, 0.7 mmol, 98 mg), and Rhodamine B (3 mol %, 7 mg) were used and then purified by column chromatography (SiO_2_, hexanes) to provide **4n** as a yellow liquid; yield 69 mg, 45%. ^1^H NMR (400 MHz, CDCl_3_): δ 7.58 (m, 4H), 7.29–7.25 (m, 1H), 7.08–7.04 (m, 2H), 6.81–6.78 (m, 1H), 3.82 (s, 3H); ^13^C­{H} NMR (100 MHz, CDCl_3_): δ 160.4, 133.4, 131.6, 130.3, 129.6, 126.7, 125.4 (q, *J* = 4.0 Hz), 122.6, 118.8, 112.7, 112.1, 96.9, 79.1, 55.4; ^19^F NMR (376 MHz, CDCl_3_) δ −62.70. HRMS (EI) calcd for C_16_H_11_F_3_OS [M]^+^ 308.0483, found 308.0490.

#### (4-Methoxyphenyl)­((4-(trifluoromethyl)­phenyl)­ethynyl)­sulfane (**4o**)

Following the general procedure, 1-(bromoethynyl)-4-(trifluoromethyl)­benzene (**1g**, 0.5 mmol, 125 mg), 4-methoxybenzenethiol (**2d**, 0.7 mmol, 98 mg), and Rhodamine B (3 mol %, 7 mg) were used and then purified by column chromatography (SiO_2_, hexanes) to provide **4o** as a yellow liquid; yield 56 mg, 36%. ^1^H NMR (400 MHz, CDCl_3_): δ 7.52–7.58 (m, 4H), 7.46–7.42 (m, 2H), 6.95–6.91 (m, 2H), 3.81 (s, 3H); ^13^C­{H} NMR (100 MHz, CDCl_3_): δ 159.4, 131.5, 129.6, 126.9, 125.3 (q, *J* = 4.0 Hz), 122.6, 122.2, 115.2, 94.8, 80.9, 55.5; ^19^F NMR (376 MHz, CDCl_3_) δ −62.68. HRMS (EI) calcd for C_16_H_11_F_3_OS [M]^+^ 308.0483, found 308.0478.

## Supplementary Material



## Data Availability

The data underlying this study are available in the published article and its Supporting Information.

## Notes

Copies of ^1^H NMR, ^13^C NMR, and ^19^F NMR spectra for the products (PDF)

## References

[1] Li G., Yan Q., Gan Z., Li Q., Dou X., Yang D. (2019). Photocatalyst-Free Visible-Light-Promoted C­(sp^2^)S Coupling: A Strategy For the Preparation of *S*-Aryl Dithiocarbamates. Org. Lett..

[2] Yu X.-Y., Chen J.-R., Xiao W.-J. (2021). Visible Light-Driven
Radical-Mediated C–C Bond Cleavage/ Functionalization in Organic
Synthesis. Chem. Rev..

[3] Pitre S. P., Overman L. E. (2022). Strategic use of
Visible-Light Photoredox
Catalysis in Natural Product Synthesis. Chem.
Rev..

[4] Allen A. R., Noten E. A., Stephenson C. R. J. (2022). Aryl
Transfer Strategies Mediated by Photoinduced Electron Transfer. Chem. Rev..

[5] Peng Y., Feng C. T., Li Y. Q., Chen F. X., Xu K. (2019). Exploring
the Ring-Opening Reactions of Imidazo [1, 5-a] quinolines
for the Synthesis of Imides under Photochemical Conditions. Org. Biomol Chem..

[6] Peiris S., McMurtrie J., Zhu H. Y. (2016). Metal Nanoparticle Photocatalysts:
Emerging Processes for Green Organic Synthesis. Cata. Sci. Technol..

[7] Yadav A. K., Yadav L. D. S. (2014). Visible-Light-Promoted Aerobic Oxidative
Cyclization to Access 1, 3, 4-Oxadiazoles from Aldehydes and Acylhydrazides. Tetrahedron Lett..

[8] Kumar R., Garima K., Srivastava V., Singh P. P., Singh P. K. (2025). Visible Light Induced Eosin Y Catalyzed
Green Synthesis of Substituted Pyrroles. Catal.
Lett..

[9] Gray V. J., Wilden J. D. (2016). The Chemistry of
Ynol and Thioynol
Ethers. Org. Biomol. Chem..

[10] Diederich, F. ; Stang, P. J. ; Tykwinski, R. R. Acetylene Chemistry: Chemistry, Biology, and Material Science, ed. Wiley-VCH, Weinheim: 2005. 2.

[11] Timothy B. C., Woerpel K. A. (2005). Silver-Catalyzed
Silacyclopropenation of 1-Heteroatom-Substituted Alkynes and Subsequent
Rearrangement Reactions. Organometallics.

[12] b Akira, N. ; Hirotaka, F. ; Keisuke, T. ; Kiminori, A. Pyrazole derivative and pest control agent. JP2014015447A, 2014.

[13] a Koichi, H. ; Yuji, I. ; Atsushi, N. ; Shunichi, M. Production of optically active azetidinone derivative. JPS61280295A, 1986.

[14] Dunbar K. L., Scharf D. H., Litomska A., Hertweck C. (2017). Enzymatic Carbon-Sulfur Bond Formation in Natural Product
Biosynthesis. Chem. Rev..

[15] Singh P., Bai R., Choudhary R., Sharma M. C., Badsara S. S. (2017). Regio- and Stereoselective Syntheses
of Allylic Thioethers under Metal Free Conditions. RSC Adv..

[16] Braga A. L., Silviera C. C., Reckziegel A., Menezes P. H. (1993). Convenient Preparation of Alkynyl Selenides, Sulfides
and Tellurides from Terminal Alkynes and Prenylchalcogenyl Halides
in the Presence of Copper (I) Iodide. Tetrahedron
Lett..

[17] Ni Z., Wang S., Mao H., Pan Y. (2012). A Concise Synthetic Strategy to Alkynyl Sulfides *via* Transition-Metal-Free Catalyzed C–S Coupling of 1,1-Dibromo-1-alkenes
with Thiophenols. Tetrahedron Lett..

[18] Guo W., Tao K., Tan W., Zhao M., Zheng L., Fan X. (2019). Recent Advances in
Photocatalytic C-S/P-S Bond Formation *via* The Generation
of Sulfur Centered Radicals and Functionalization. Org. Chem. Front..

[19] Liu B., Lim C.-H., Miyake G. M. (2017). Visible-Light-Promoted
C-S Cross-Coupling *via* Intermolecular Charge Transfer. J. Am. Chem. Soc..

[20] Xuan F., Feng Z.-F., Chen J.-R., Lu L.-Q., Xiao W.-J. (2014). Visible-Light-Induced C-S Bond Activation:
Facile Access to 1,4-Diketones from β-Ketosulfones. Chem. - Eur. J..

[21] Santandrea J., Minozzi C., Cruché C., Collins S. K. (2017). Photochemical dual-catalytic synthesis of alkynyl sulfides. Angew. Chem., Int. Ed..

[22] Marino J. P., Nguyen H. N. (2002). Bulky Trialkylsilyl
Acetylenes in the Cadiot-Chodkiewicz Cross-Coupling Reaction. J. Org. Chem..

[23] Gao W. C., Shang Y. Z., Chang H. H., Li X., Wei W. L., Yu X. Z., Zhou R. (2019). N-Alkynylthio Phthalimide:
A Shelf-Stable Alkynylthio Transfer Reagent for the Synthesis of Alkynyl
Thioethers. Org. Lett..

[24] Zhang Y. Q., Zhu X. Q., Chen Y. B., Tan T. D., Yang M. Y., Ye L. W. (2018). Synthesis of Isothiochroman-3-ones *via* Metal-Free Oxidative Cyclization of Alkynyl Thioethers. Org. Lett..

