# Supplementary material for: Visible-Light-Triggered and Rhodamine B‑Catalyzed Thiolation of Bromoalkynes: Accessing Alkynyl Thioethers
Source: J Org Chem. 2025 Jul 23;90(30):10880–91. doi: 10.1021/acs.joc.5c01295 (PMC12322964; doi:10.1021/acs.joc.5c01295)
Supplement: Supplementary file 1 [file jo5c01295_si_001.pdf]

Visible-Light-Triggered and Rhodamine B-catalyzed Thiolation of Bromoalkynes: Accessing Alkynyl Thioethers

Rekha Bai[a], Yu-Ling Chiu[a], Indrajit Karmakar[a] and Chin-Fa Lee*[a,b,c]

[a] Department of Chemistry, National Chung Hsing University, Taichung, Taiwan 402, R.O.C.
Email: cfalee@dragon.nchu.edu.tw

[b] *i*-Center for Advanced Science and Technology (*i*CAST), National Chung Hsing University, Taichung, Taiwan 402, R.O.C.

[c] Innovation and Development Center of Sustainable Agriculture (IDCSA), National Chung Hsing University, Taichung, Taiwan 402, R.O.C.

Entry	Contents	Page No.
1	Stern-Volmer studies	S2-S5
2	Quantum yield calculations	S5-S6
3	Reference	S6
4	Copies of ^{1}H, ^{13}C and ^{19}F-NMR Spectra	S7-S81
5	Copies of HRMS of TEMPO and BHT adducts	S82-S83

Fluorescence quenching studies (Stern–Volmer Studies)

The fluorescence emission intensities were recorded on a F-7000 FL Spectrophotometer. The excitation wavelength was set at 320 nm, and the emission wavelength was measured over the range of 330-750 nm. Samples were prepared by mixing Rhodamine B (3.0×10^{-5} mol/L) with varying concentration of (bromoethynyl)benzene (**1a**) in DMSO (total volume = 2.0 mL) in a quartz fluorescence cuvette. For each quenching experiment, 1.0 mL of (bromoethynyl)benzene stock solution at different concentration was added to 1.0 mL of the Rhodamine B solution, yielding a total volume of 2.0 mL. Then the emission intensity was collected and the data were presented in Figure S1.

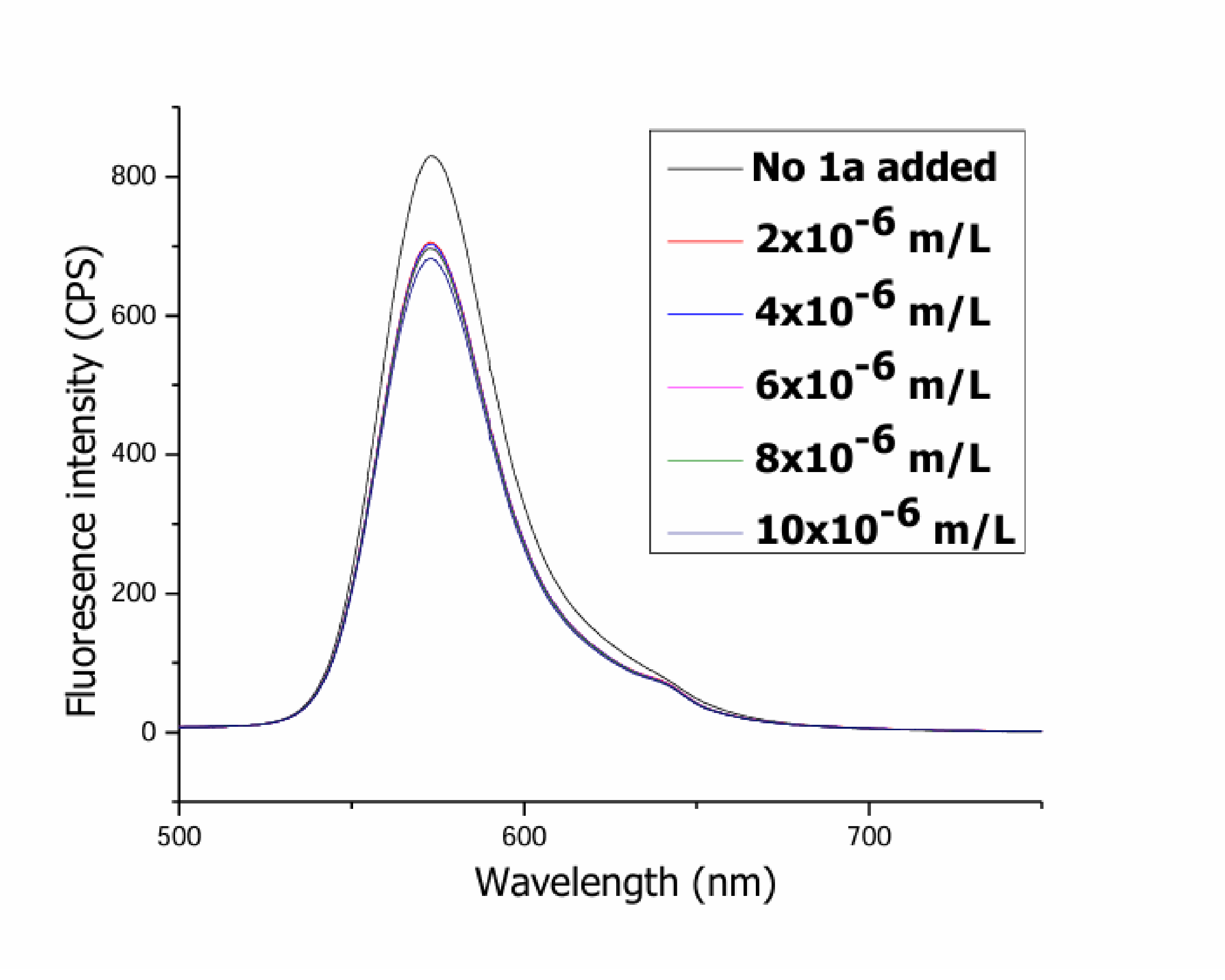

Figure S1: Quenching of Rhodamine B fluorescence emission in the presence of

(bromoethynyl)benzene (**1a**).

In this case, no significant fluorescence quenching of Rhodamine B was observed with increasing concentrations of (bromoethynyl)benzene (**1a**), as shown by the Stern–Volmer plot ([I_0/I] vs. C[**1a**]) in Figure S2. The linearity of the Stern–Volmer plot indicates that no energy transfer occurs between the excited state of Rhodamine B and (bromoethynyl)benzene (**1a**).

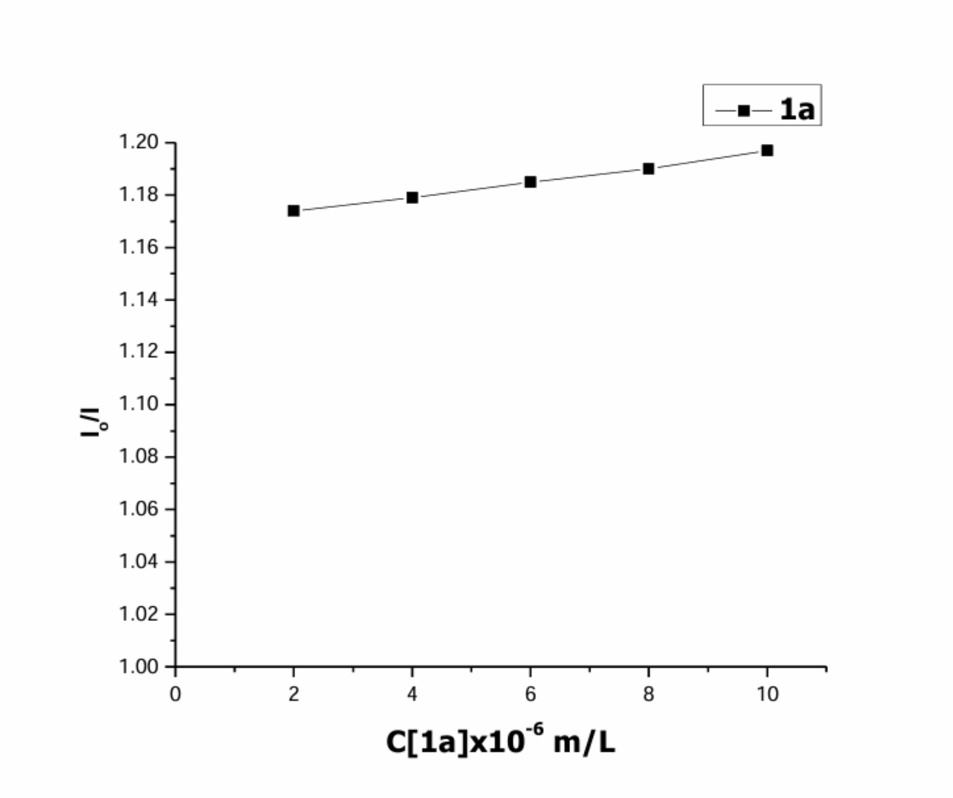

Figure S2. Stern-Volmer plots

The fluorescence emission intensities were recorded on a F-7000 FL Spectrophotometer. The excitation wavelength was set at 320 nm, and the emission wavelength was measured over the range of 330-750 nm. Samples were prepared by mixing Rhodamine B (3.0×10^{-5} mol/L) with varying concentration of 3-methoxybenzenethiol (**2a**) in DMSO (total volume = 2.0 mL) in a quartz fluorescence cuvette. For each quenching experiment, 1.0 mL of 3-methoxybenzenethiol (**2a**) stock solution at different concentration was added to 1.0 mL of the Rhodamine B solution, yielding a total volume of 2.0 mL. Then the emission intensity was collected and the data were presented in Figure S3.

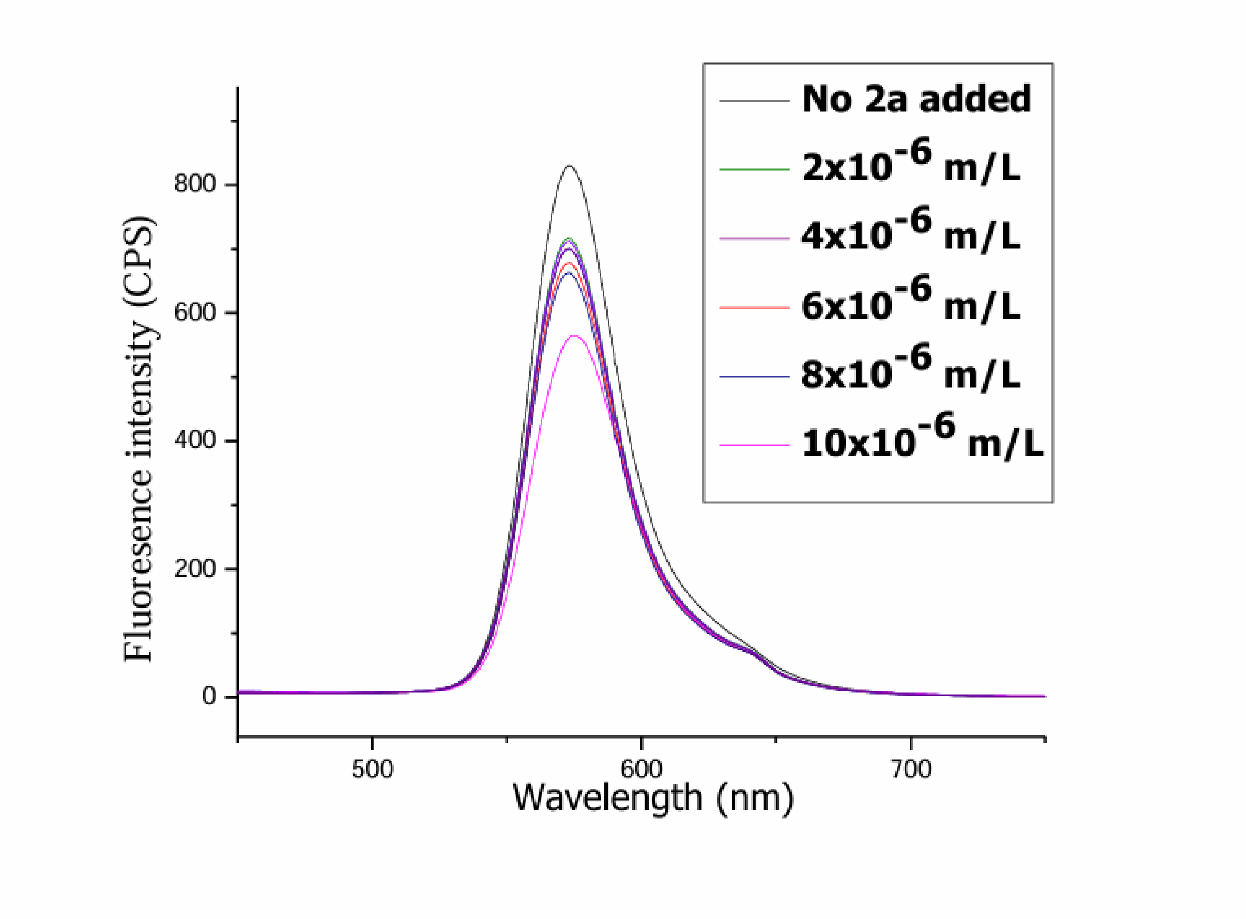

Figure S3: Quenching of Rhodamine B fluorescence emission in the presence of 3-methoxybenzene thiol (**2a**).

A clear fluorescence quenching effect of Rhodamine B was observed with increasing concentrations of 3-methoxybenzenethiol (**2a**), as illustrated by the Stern–Volmer plot ([I_0/I] vs. C[**2a**]) in Figure S4. Notably, at a concentration of 2×10^{-6} mol/L of **1a**, the non-linear nature of the Stern–Volmer plot suggests that an energy transfer process occurs between the excited state of Rhodamine B and 3-methoxybenzenethiol (**2a**).

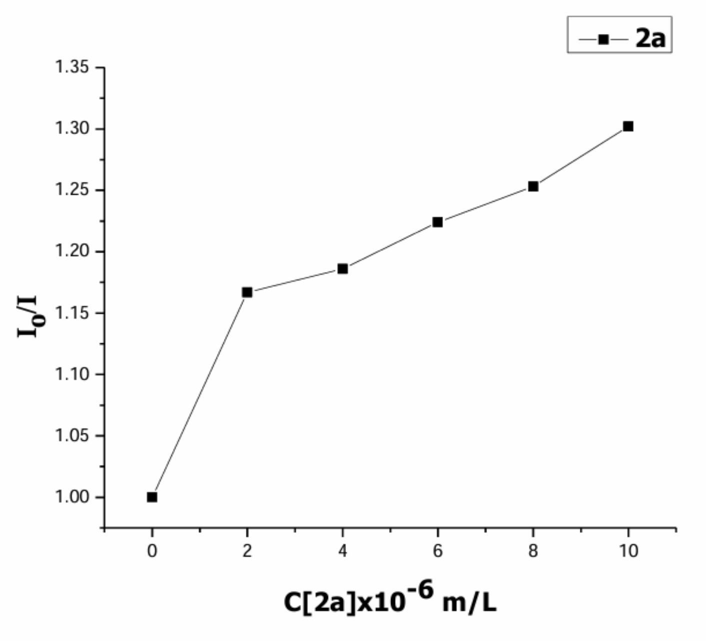

Figure S4. Stern-Volmer plots

Determination of the reaction quantum yield

The actinometry measurements were done as follows based on previous literature[5]

The photon flux of the LED light source (λmax = 405) was determined by using standard ferrioxalate actinometry. A 0.15 M potassium ferrioxalate solution was preparing by dissolving 1.474 g of potassium ferrioxalate hydrate in 20 mL of 0.05 M sulfuric acid. Separately, a buffered 1,10-phenanthroline solution was prepared by dissolving 5.0 mg of 1,10 phenanthroline and 1.13 g of sodium acetate in 5 mL of 0.5 M sulfuric acid. Both solutions were protected from light and stored in the dark. For the actinometric measurement, 3.0 mL of the ferrioxalate solution was transferred to a cuvette and irradiated at 410 nm for 90 seconds. After irradiation, 0.525 mL of the buffered phenanthroline solution was added to the cuvette. The mixture was then kept in the dark for 1 hour to allow the Fe^{2+} ions completely coordinate to the phenanthroline. The absorbance of the resulting complex was measured at 510 nm. A non-irradiated sample was also prepared, and its absorbance was recorded under identical conditions.

mol Fe^{2+} = (V × ΔA)/ (l× ε) 2.833 mol

Fe^{2+}= [3.525×10-3 L× (2.833-0.076)]/ (1 cm × 11100 L mol-1cm-1) =8.7553×10^{-7} mol

V is the total volume (0.003525 L) of the solution after addition of phenanthroline, ΔA is the difference in absorbance at 510 nm between the irradiated and non-irradiated solutions, l is the pathlength (1.00 cm), and ε is the molar absorptivity of the ferrioxalate actinometer at 510 nm (11,100Lmol-1cm-1)

photo flux = mol Fe^{2+}/ (Φ × t × f)

photo flux =8.7553×10-7/ (1.12 ×90 × 0.9998) = 8.1219 × 10^{-8} Einstein s^{-1}

Where Φ is the quantum yield for the ferrioxalate actinometer (1.12 at λ = 410 nm) is the irradiation time (90 s), and f is the fraction of light absorbed at 410 nm by the ferrioxalate actinometer. This value is calculated using, where A410 nm is the absorbance of the ferrioxalate solution at 410 nm. An absorption spectrum gave an A410 nm value of 3.664.

f = 1 − 10^{-A}410 nm = 0.9998.

The reaction mixture was irradiated by purple LED (λmax = 410 nm) for 4 h. The yield of product 3a was determined by 1H NMR analysis using 1,3,5-trimethoxybenzene as an internal standard. The yield of 3a was determined to be 25% (1.25x10^{-4} mol).

The quantum yield (Φ) = moles of product formed/ (flux × f × t)

= 1.25 × 10^{-4} mol /(8.1219 × 10^{-8} ×0.9998 ×7200) = 0.21

Where the photon flux is 8.1219 × 10^{-8} einsteins s^{-1}, t is the reaction irradiation time (2 × 60 ×60 7200 s) and f is the fraction of incident light absorbed by the catalyst.

References:

S1. (a) Li, Y.; Xu, J.; Wang, Y.; Xu, R.; Zhao, Y.; Li, W. 2025. Multicomponent Synthesis of Alkyl BCP-Heteroaryls *via* Electron Donor–Acceptor Complex Photoactivation under Mild Conditions. *J. Org. Chem.* **2025**, *90*, 1683-1696. (b) Cismesiaa, M. A.; Yoon, T. P. Characterizing chain processes in visible light photoredox catalysis. *Chem. Sci.* **2015**, *6*, 5426-5434.

Copies of ^{1}H, ^{13}C and ^{19}F-NMR

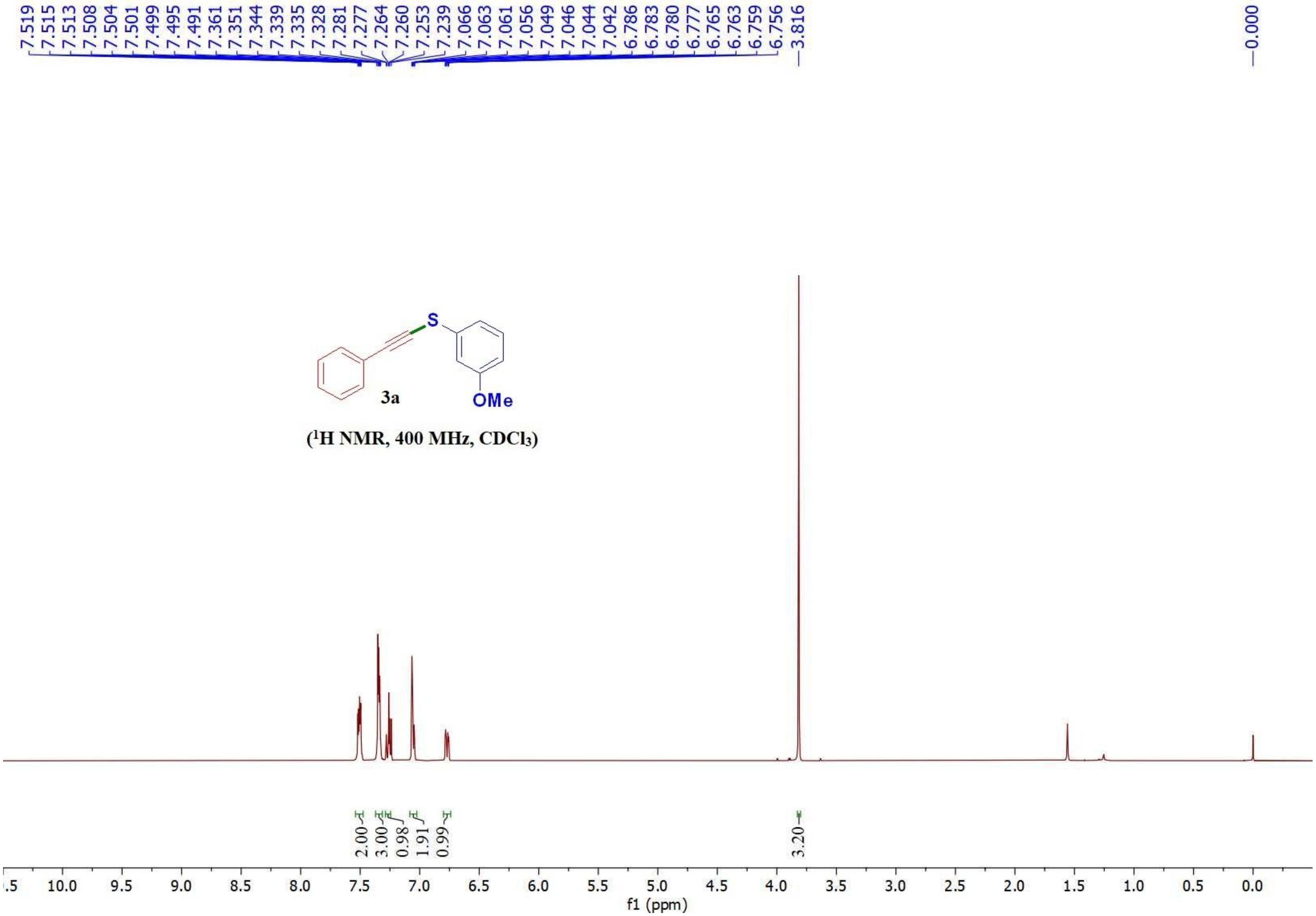
7.519
7.515
7.513
7.508
7.504
7.501
7.499
7.495
7.491
7.361
7.351
7.344
7.339
7.335
7.328
7.281
7.277
7.264
7.260
7.253
7.239
7.066
7.063
7.061
7.056
7.049
7.046
7.044
7.042
6.786
6.783
6.780
6.777
6.765
6.763
6.759
6.756
3.816
0.000
S
3a
OMe
(1H NMR, 400 MHz, CDCl3)
2.00
3.00
0.98
1.91
0.99
3.20
10.0
9.5
9.0
8.5
8.0
7.5
7.0
6.5
6.0
5.5
5.0
4.5
4.0
3.5
3.0
2.5
2.0
1.5
1.0
0.5
0.0
f1 (ppm)

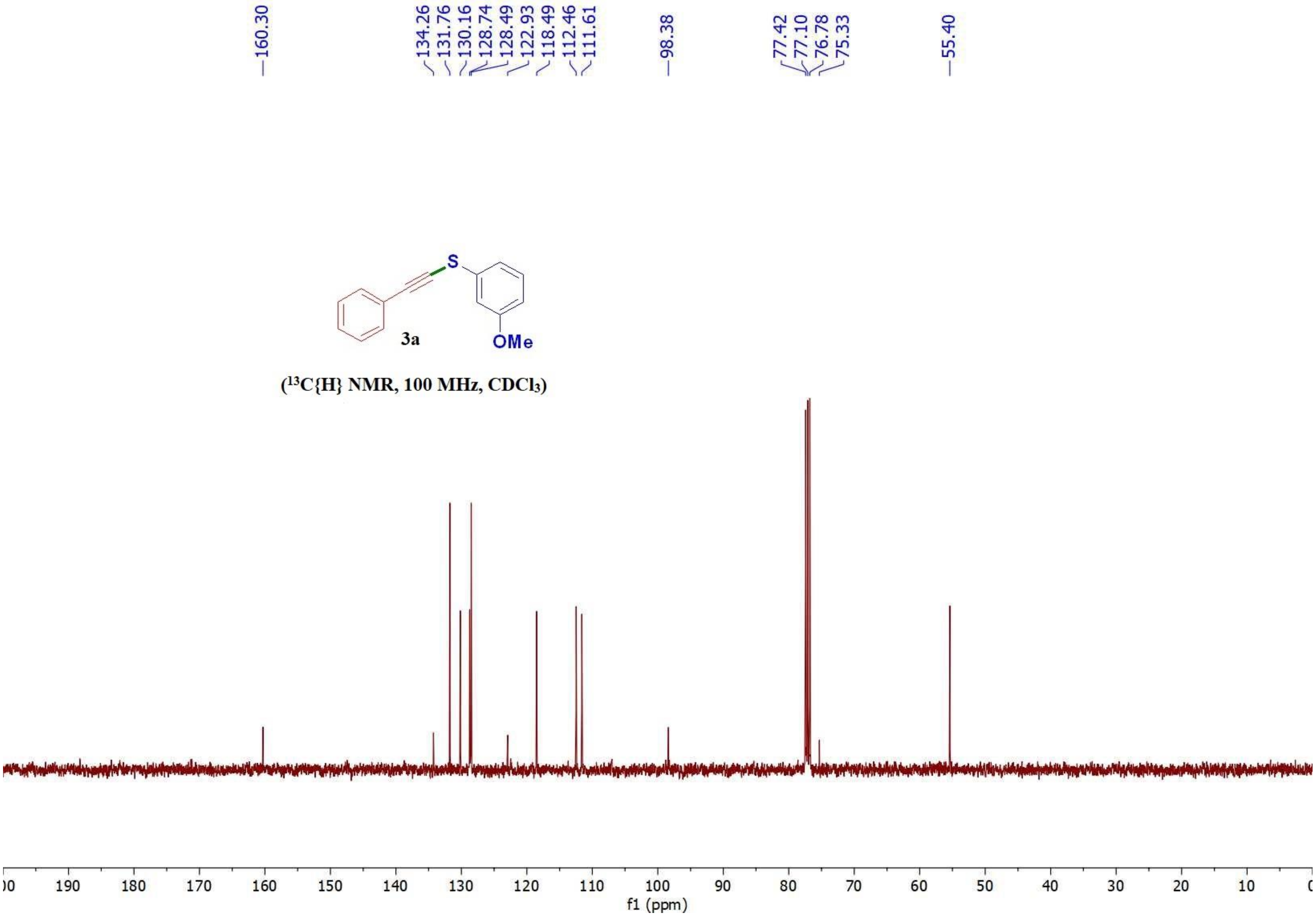

(^{13}C{H} NMR, 100 MHz, $CDCl_3$)

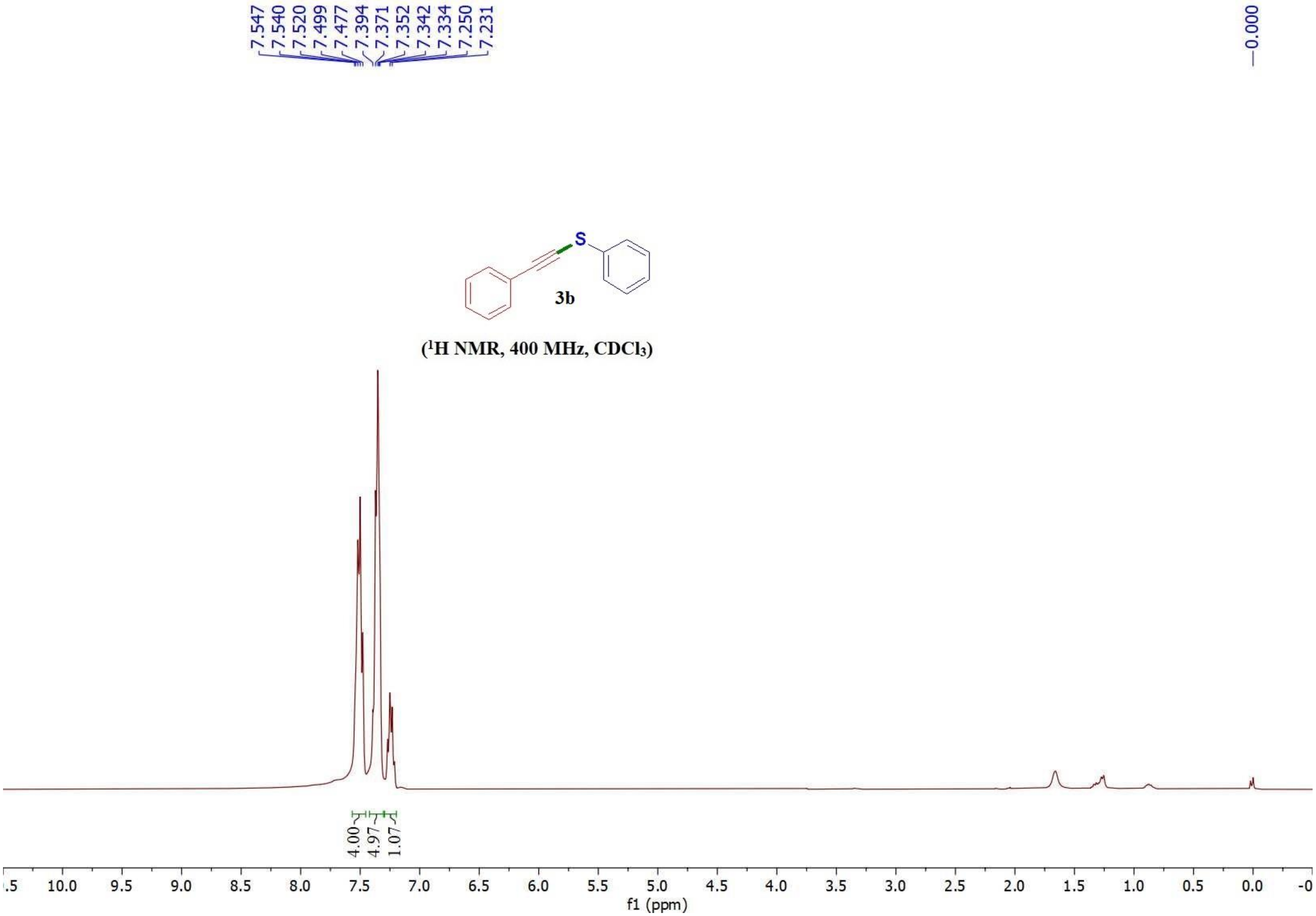
7.547
7.540
7.520
7.499
7.477
7.394
7.371
7.352
7.342
7.334
7.250
7.231
0.000
S
3b
(¹H NMR, 400 MHz, CDCl₃)
4.00
4.97
1.07
.5
10.0
9.5
9.0
8.5
8.0
7.5
7.0
6.5
6.0
5.5
5.0
4.5
4.0
3.5
3.0
2.5
2.0
1.5
1.0
0.5
0.0
-0
f1 (ppm)

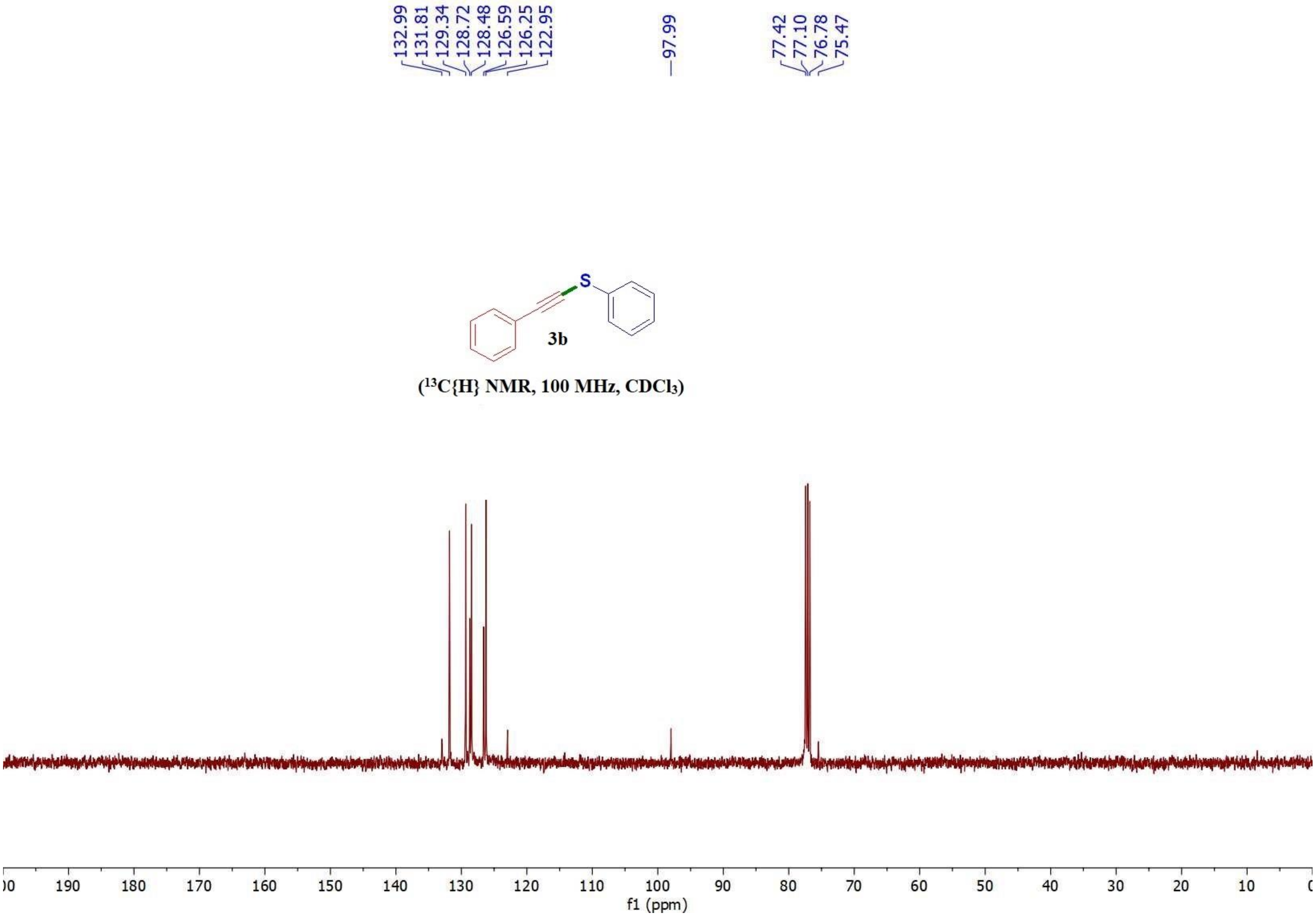
132.99
131.81
129.34
128.72
128.48
126.59
126.25
122.95
97.99
77.42
77.10
76.78
75.47
S
3b
(^{13}C{H} NMR, 100 MHz, $CDCl_3$)
f1 (ppm)

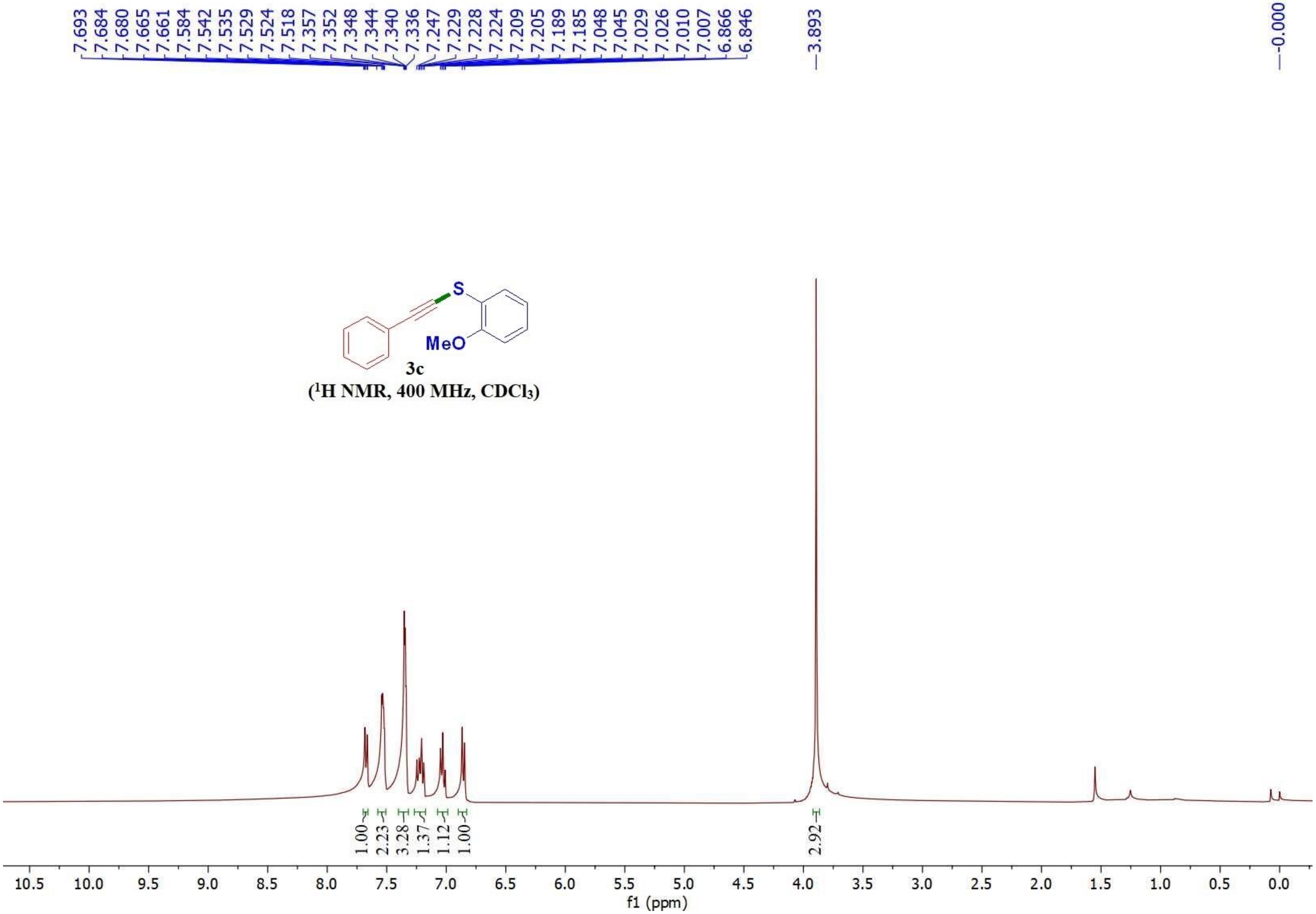

3c
(^{1}H NMR, 400 MHz, $CDCl_3$)

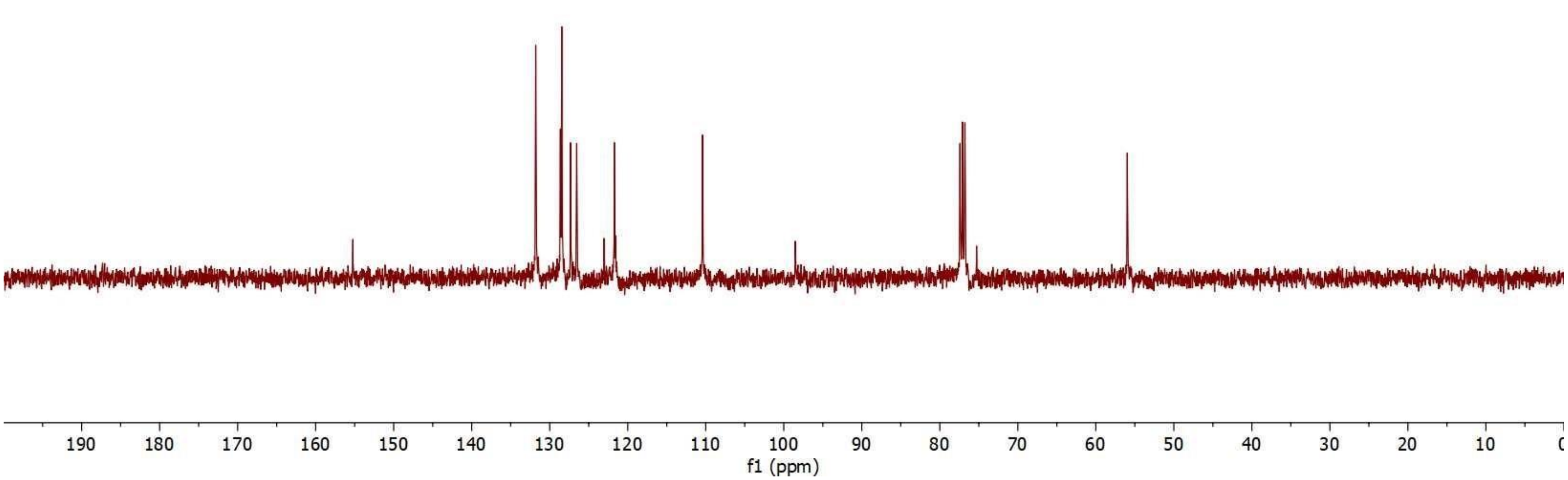

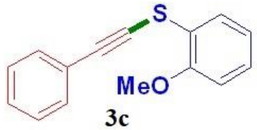

3c

(^{13}C{H} NMR, 100 MHz, CDCl$_3$)

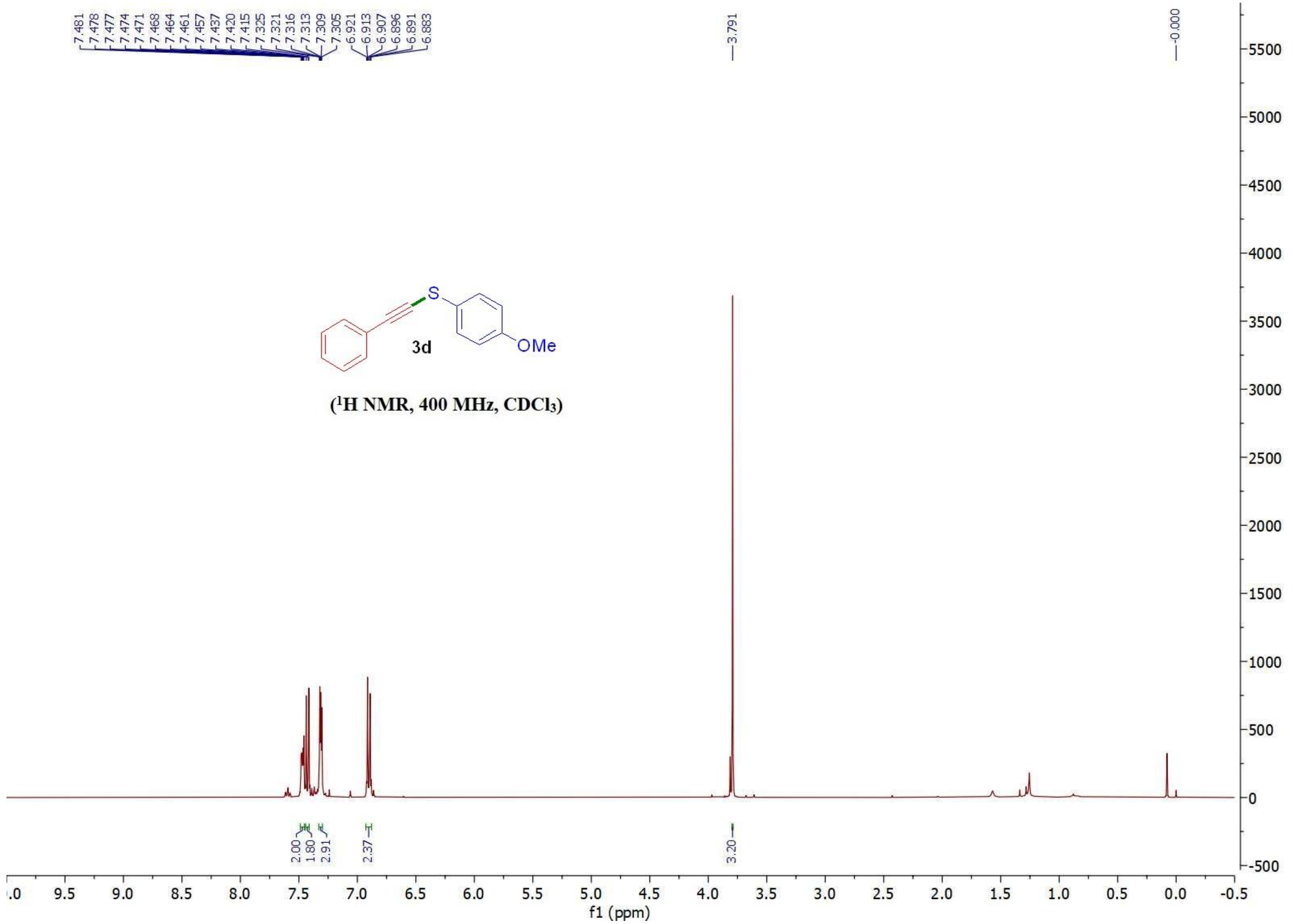

7.481
7.478
7.477
7.474
7.471
7.468
7.464
7.461
7.457
7.437
7.420
7.415
7.325
7.321
7.316
7.313
7.309
7.305
6.921
6.913
6.907
6.896
6.891
6.883
3.791
0.000
S
OMe
3d
(1H NMR, 400 MHz, CDCl3)
2.00
1.80
2.91
2.37
3.20
5500
5000
4500
4000
3500
3000
2500
2000
1500
1000
500
0
-500
9.5
9.0
8.5
8.0
7.5
7.0
6.5
6.0
5.5
5.0
4.5
4.0
3.5
3.0
2.5
2.0
1.5
1.0
0.5
0.0
-0.5
f1 (ppm)

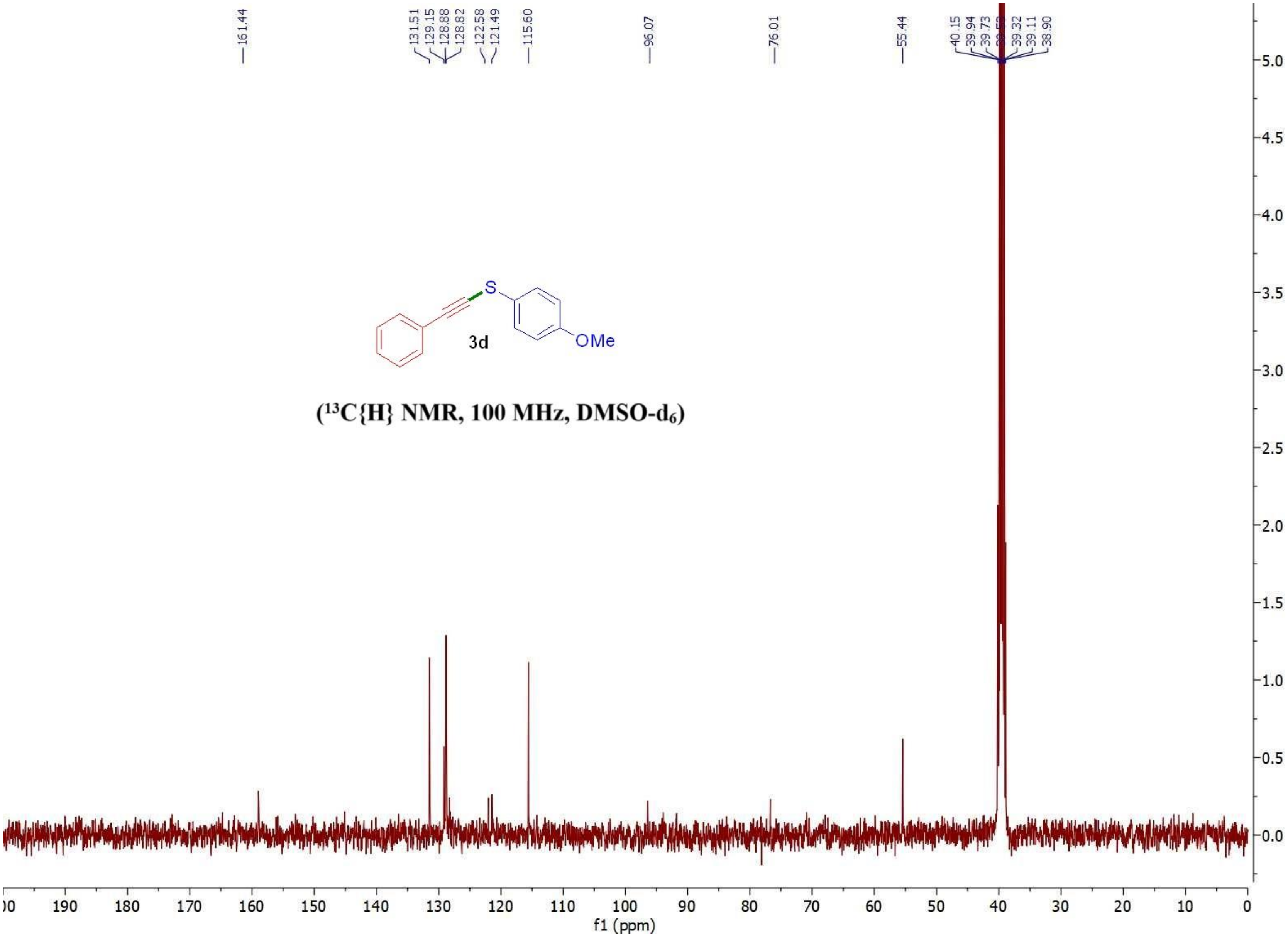
161.44
131.51
129.15
128.88
128.82
122.58
121.49
115.60
96.07
76.01
55.44
40.15
39.94
39.73
39.32
39.11
38.90
S
OMe
3d
(^{13}C{H} NMR, 100 MHz, DMSO-d$_6$)
f1 (ppm)

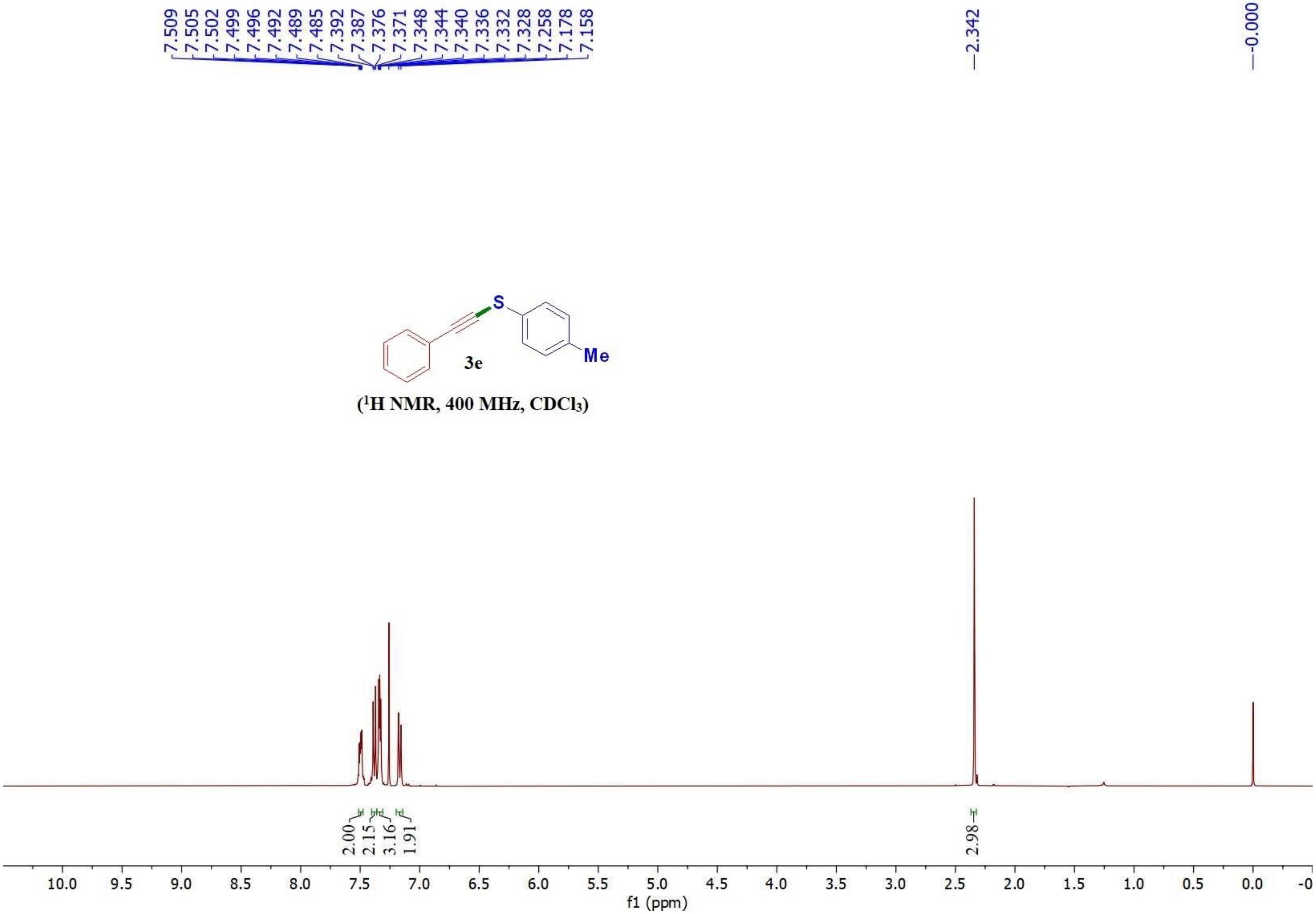
7.509
7.505
7.502
7.499
7.496
7.492
7.489
7.485
7.392
7.387
7.376
7.371
7.348
7.344
7.340
7.336
7.332
7.328
7.258
7.178
7.158
2.342
-0.000
S
Me
3e
(¹H NMR, 400 MHz, CDCl₃)
2.00
2.15
3.16
1.91
2.98
10.0
9.5
9.0
8.5
8.0
7.5
7.0
6.5
6.0
5.5
5.0
4.5
4.0
3.5
3.0
2.5
2.0
1.5
1.0
0.5
0.0
-0
f1 (ppm)

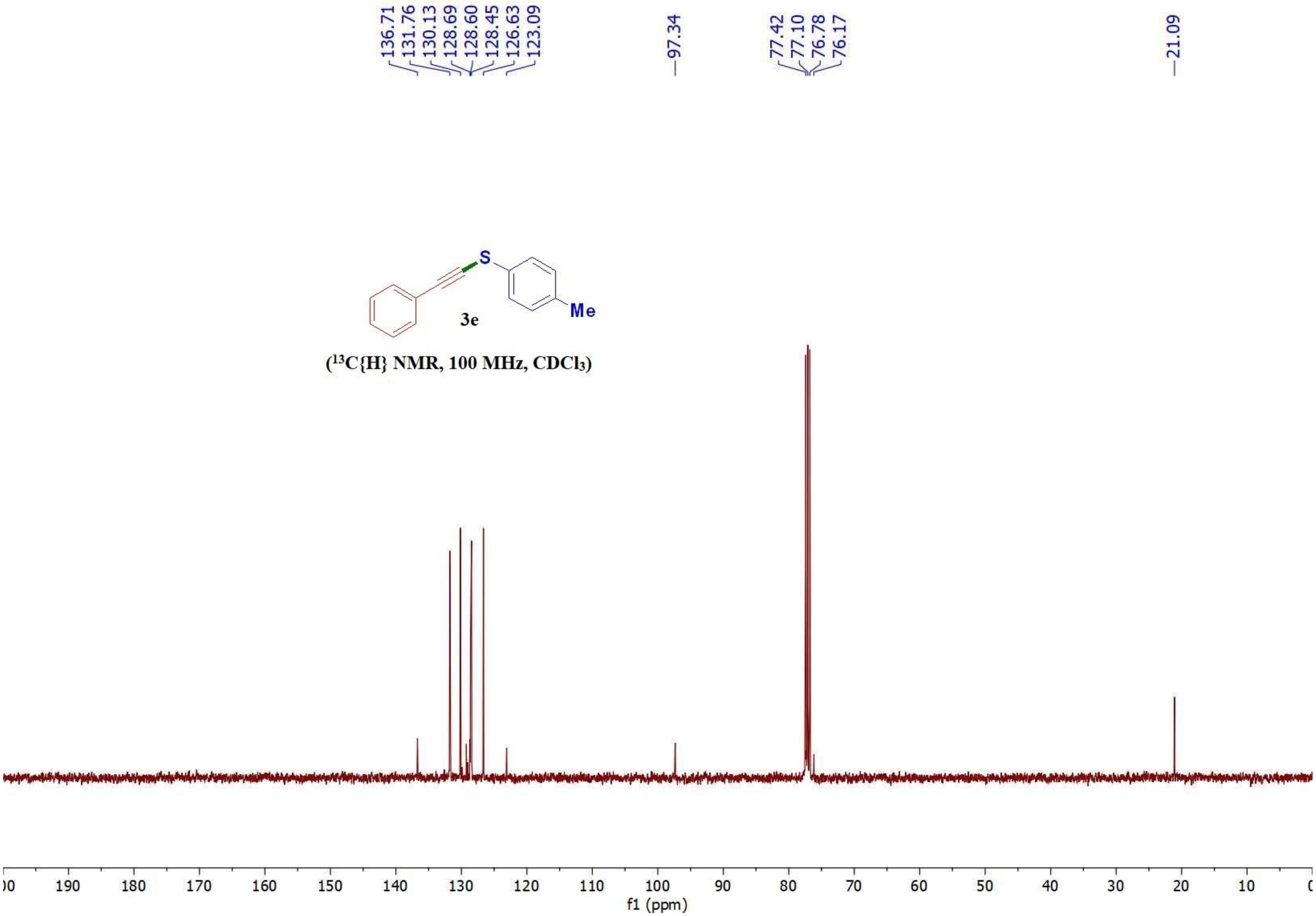
136.71
131.76
130.13
128.69
128.60
128.45
126.63
123.09
97.34
77.42
77.10
76.78
76.17
21.09
S
Me
3e
(^{13}C{H} NMR, 100 MHz, CDCl$_3$)
190
180
170
160
150
140
130
120
110
100
90
80
70
60
50
40
30
20
10
f1 (ppm)

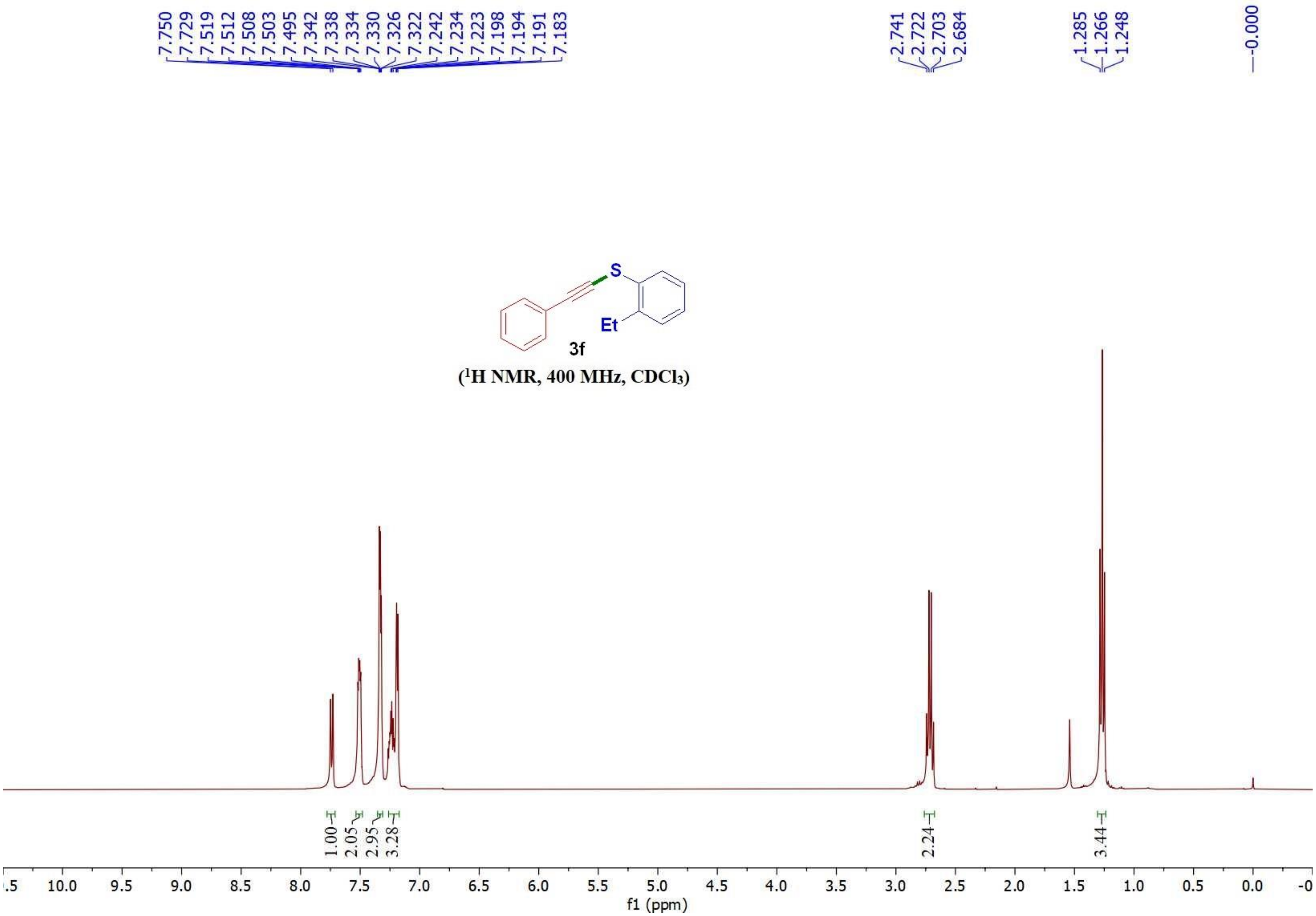

7.750
7.729
7.519
7.512
7.508
7.503
7.495
7.342
7.338
7.334
7.330
7.326
7.322
7.242
7.234
7.223
7.198
7.194
7.191
7.183
2.741
2.722
2.703
2.684
1.285
1.266
1.248
-0.000
S
Et
3f
(¹H NMR, 400 MHz, CDCl₃)
1.00
2.05
2.95
3.28
2.24
3.44
f1 (ppm)

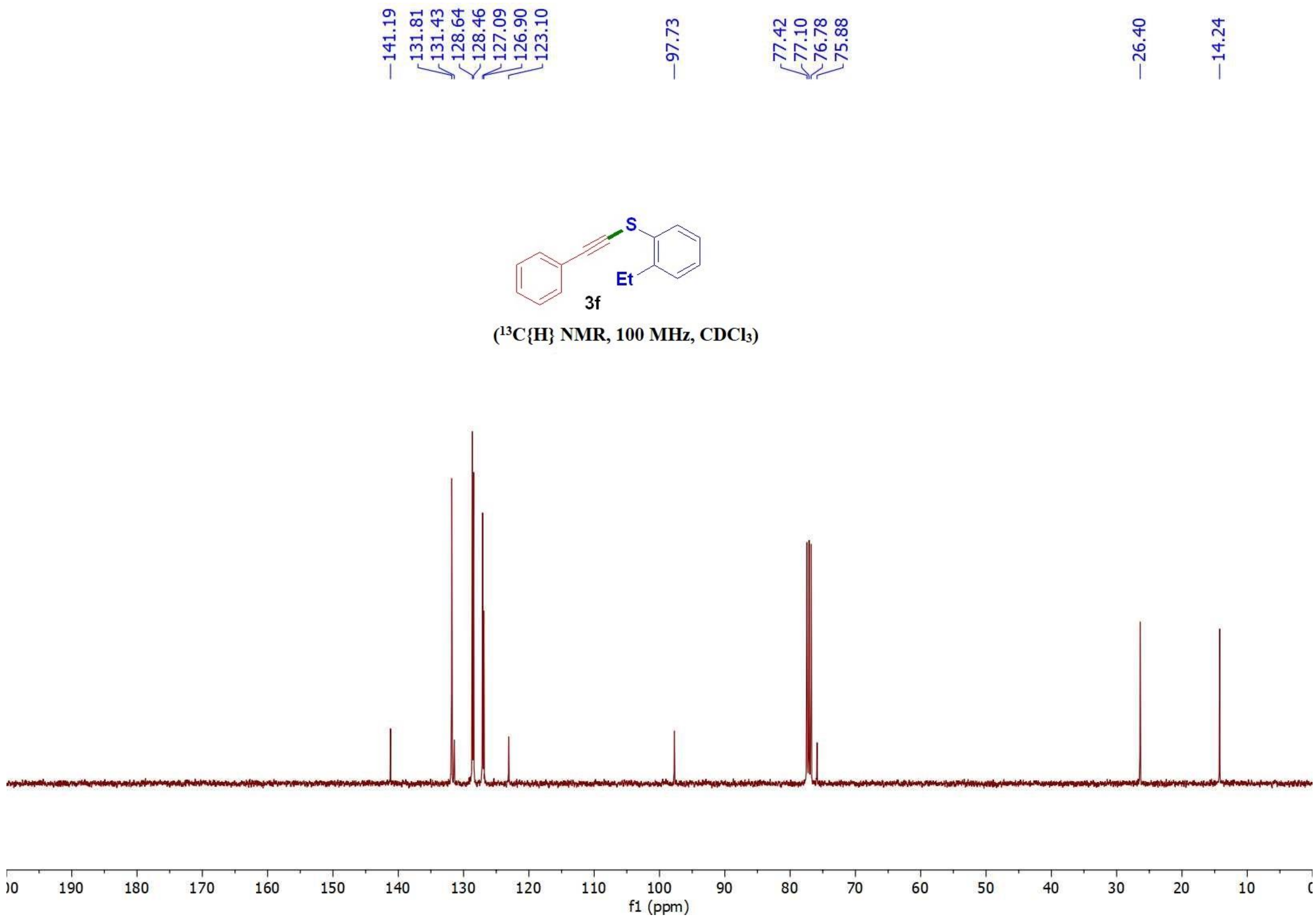
141.19
131.81
131.43
128.64
128.46
127.09
126.90
123.10
97.73
77.42
77.10
76.78
75.88
26.40
14.24
S
Et
3f
(13C{H} NMR, 100 MHz, CDCl3)
190
180
170
160
150
140
130
120
110
100
90
80
70
60
50
40
30
20
10
f1 (ppm)

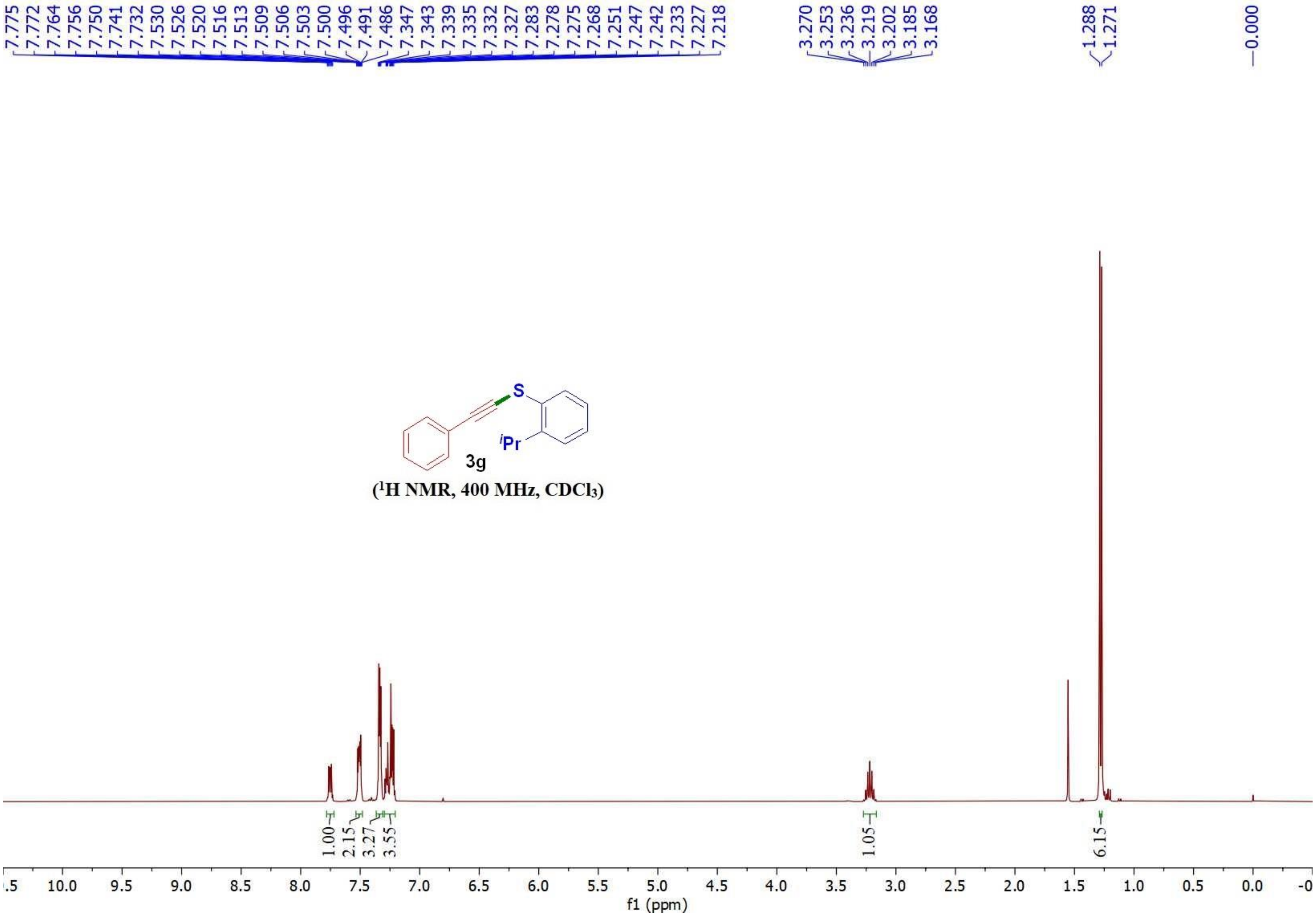
7.775 7.772 7.764 7.756 7.750 7.741 7.732 7.530 7.526 7.520 7.516 7.513 7.509 7.506 7.503 7.500 7.496 7.491 7.486 7.347 7.343 7.339 7.335 7.332 7.327 7.283 7.278 7.275 7.268 7.251 7.247 7.242 7.233 7.227 7.218
3.270 3.253 3.236 3.219 3.202 3.185 3.168
1.288 1.271
0.000
S
iPr
3g
(1H NMR, 400 MHz, CDCl3)
1.00 2.15 3.27 3.55
1.05
6.15
.5 10.0 9.5 9.0 8.5 8.0 7.5 7.0 6.5 6.0 5.5 5.0 4.5 4.0 3.5 3.0 2.5 2.0 1.5 1.0 0.5 0.0 -0
f1 (ppm)

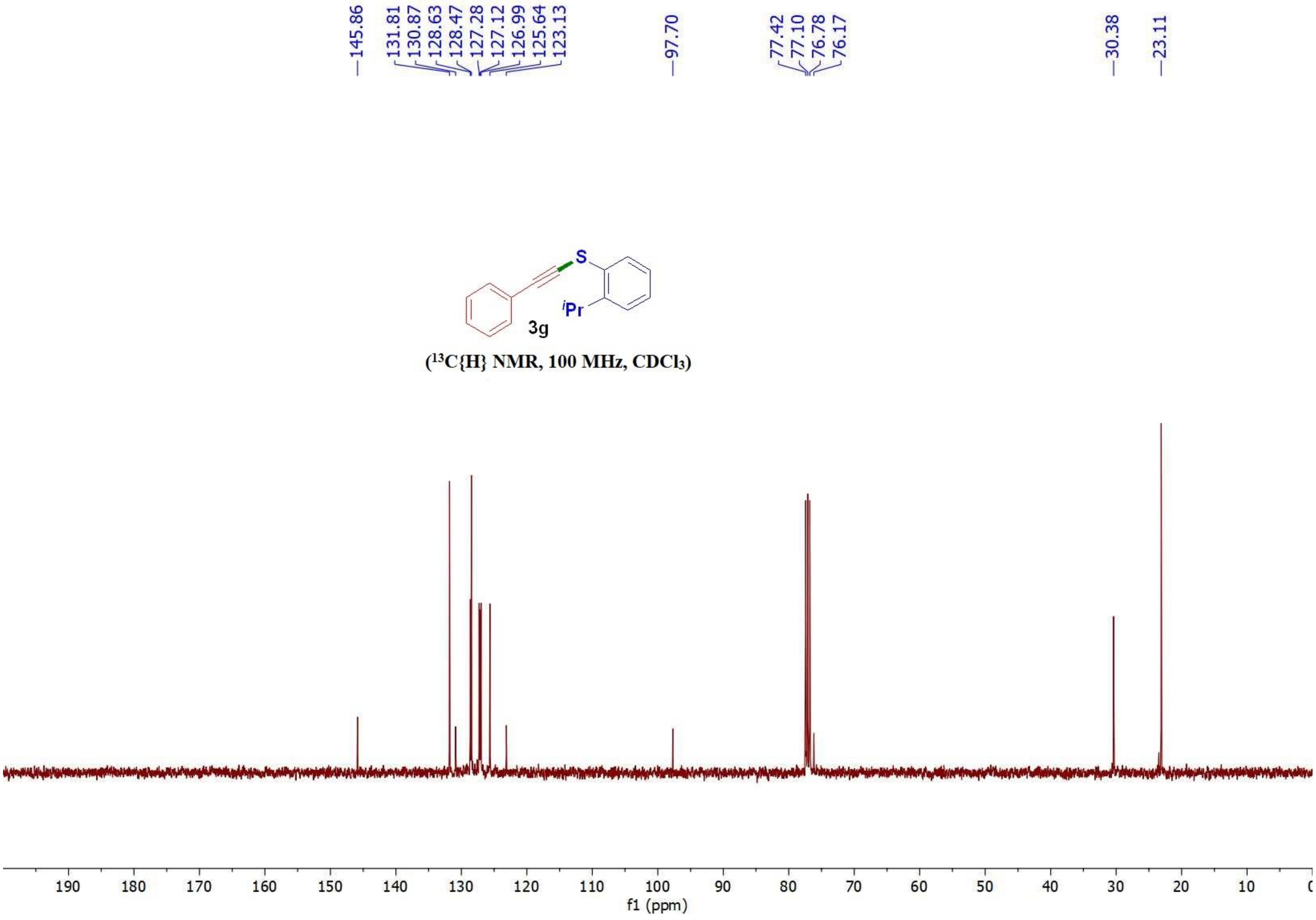

($^{13}C\{H\}$ NMR, 100 MHz, $CDCl_3$)

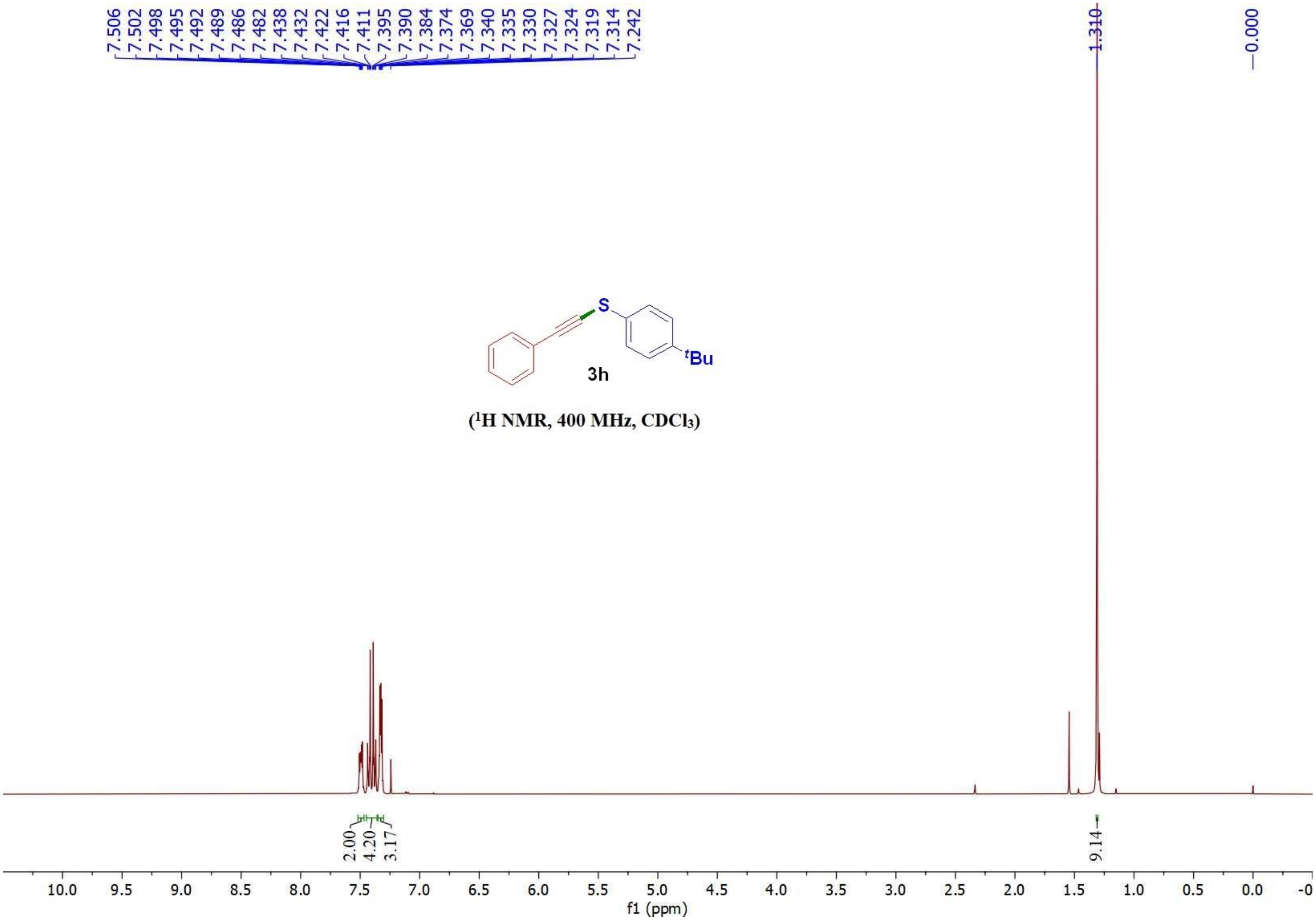
7.506
7.502
7.498
7.495
7.492
7.489
7.486
7.482
7.438
7.432
7.422
7.416
7.411
7.395
7.390
7.384
7.374
7.369
7.340
7.335
7.330
7.327
7.324
7.319
7.314
7.242
1.310
0.000
S
tBu
3h
(1H NMR, 400 MHz, CDCl3)
2.00
4.20
3.17
9.14
f1 (ppm)

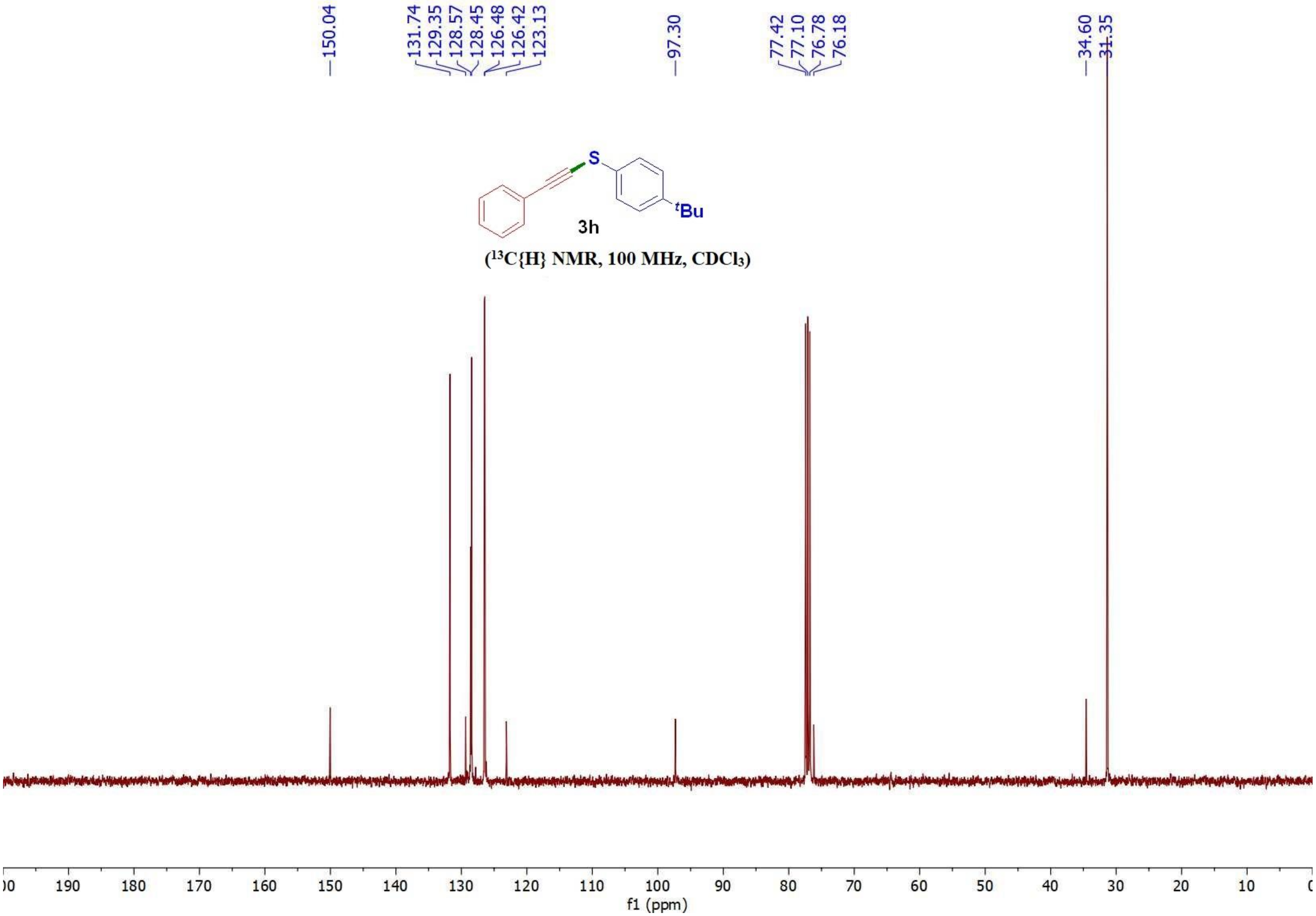
150.04
131.74
129.35
128.57
128.45
126.48
126.42
123.13
97.30
77.42
77.10
76.78
76.18
34.60
31.35
S
tBu
3h
(13C{H} NMR, 100 MHz, CDCl3)
f1 (ppm)

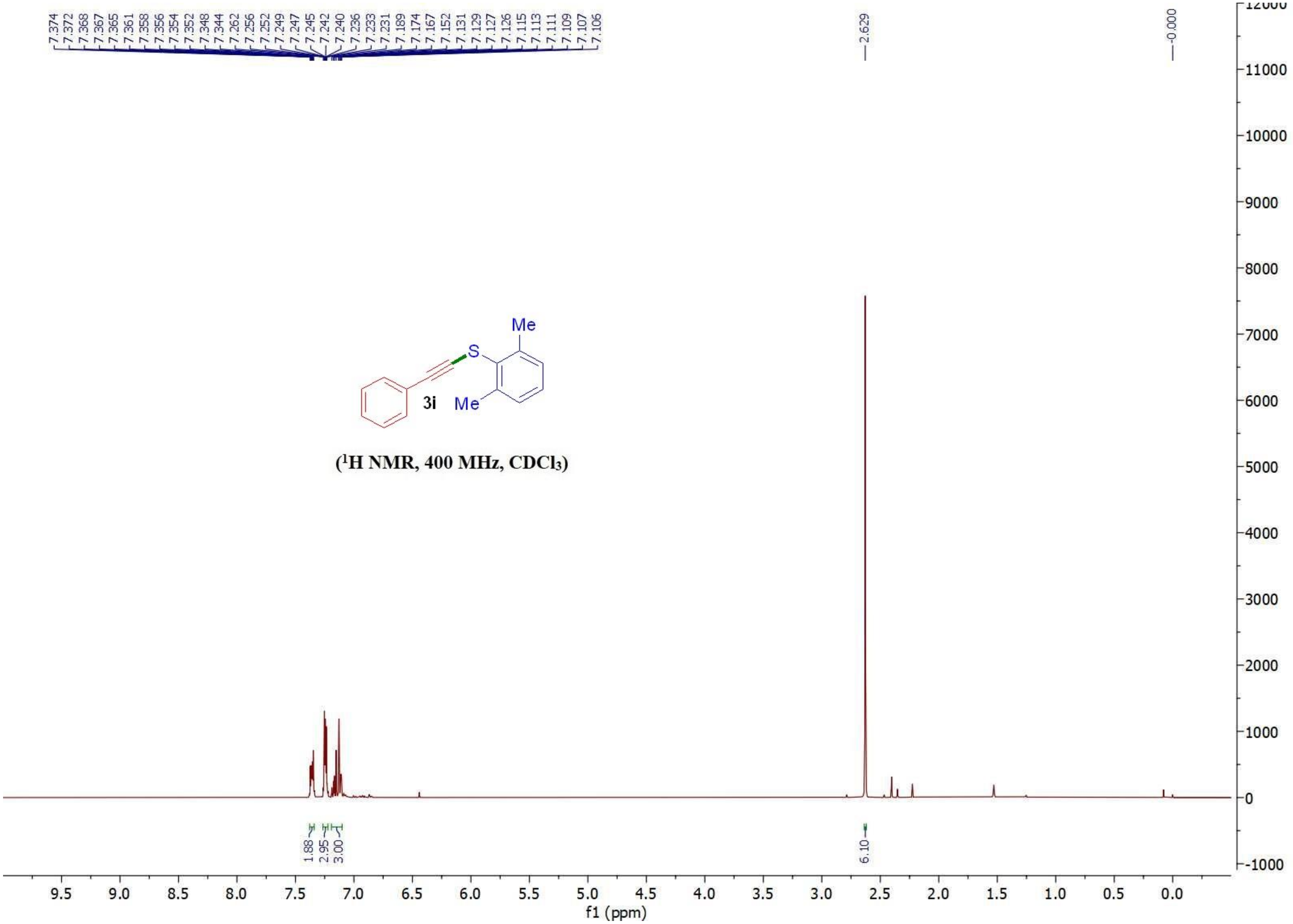
7.374
7.372
7.368
7.367
7.365
7.361
7.358
7.356
7.354
7.352
7.348
7.344
7.262
7.256
7.252
7.249
7.247
7.245
7.242
7.240
7.236
7.233
7.231
7.189
7.174
7.167
7.152
7.131
7.129
7.127
7.126
7.115
7.113
7.111
7.109
7.107
7.106
2.629
-0.000
Me
S
3i
Me
(¹H NMR, 400 MHz, CDCl₃)
1.88
2.95
3.00
6.10
f1 (ppm)

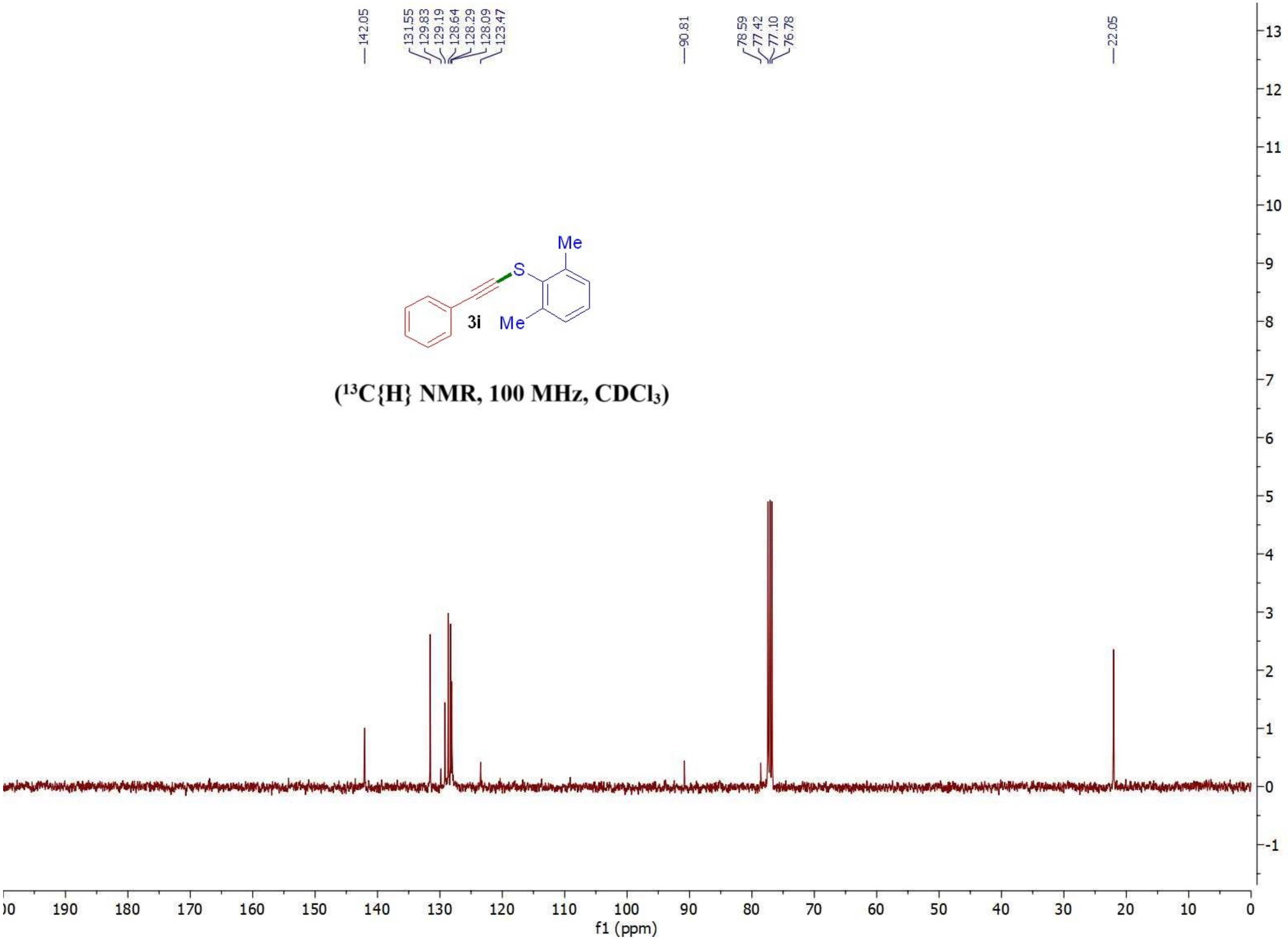

142.05
131.55
129.83
129.19
128.64
128.29
128.09
123.47
90.81
78.59
77.42
77.10
76.78
22.05
S
Me
Me
3i
(13C{H} NMR, 100 MHz, CDCl3)
f1 (ppm)

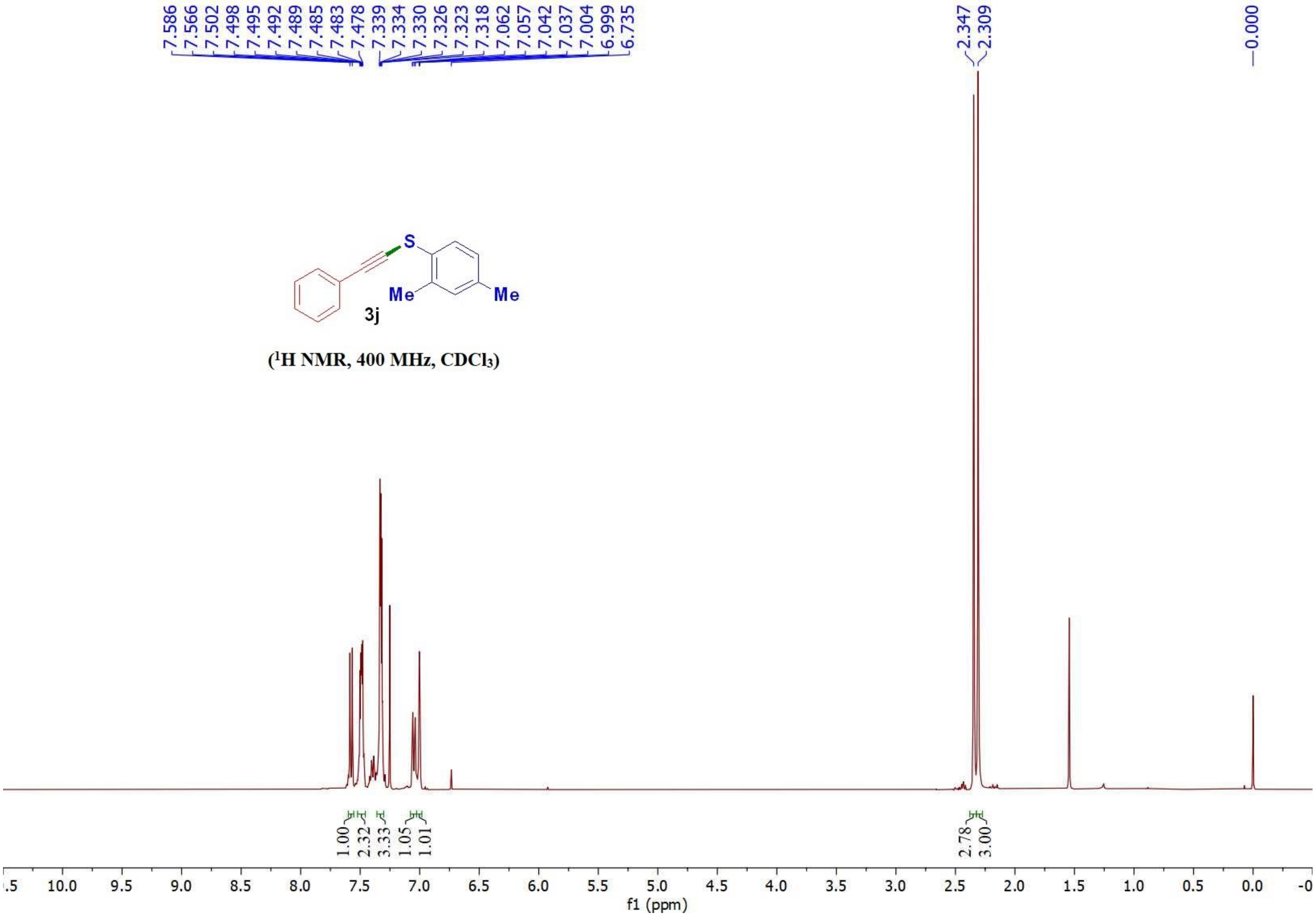

(^{1}H NMR, 400 MHz, $CDCl_3$)

3j

($^{13}C\{H\}$ NMR, 100 MHz, $CDCl_3$)

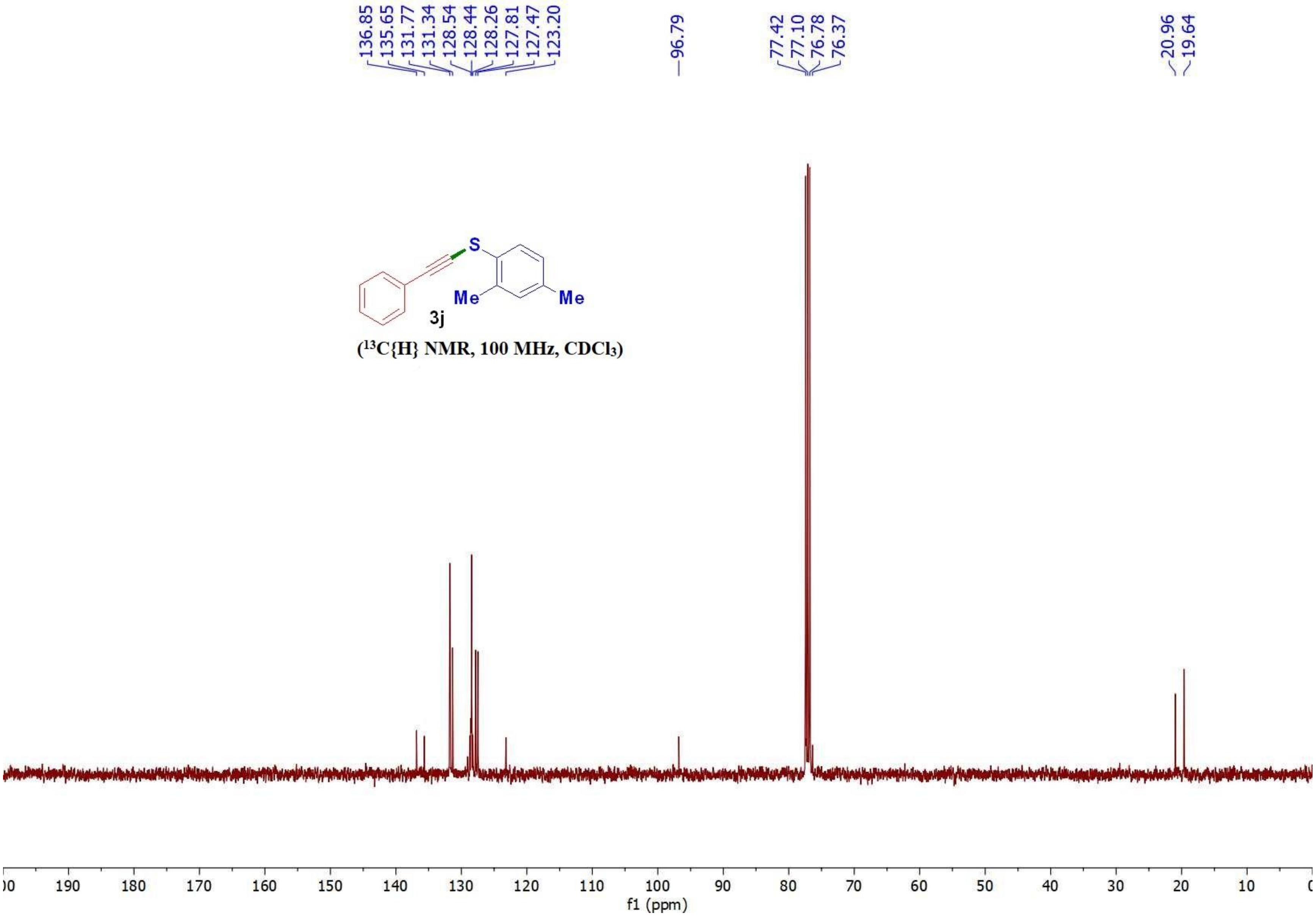

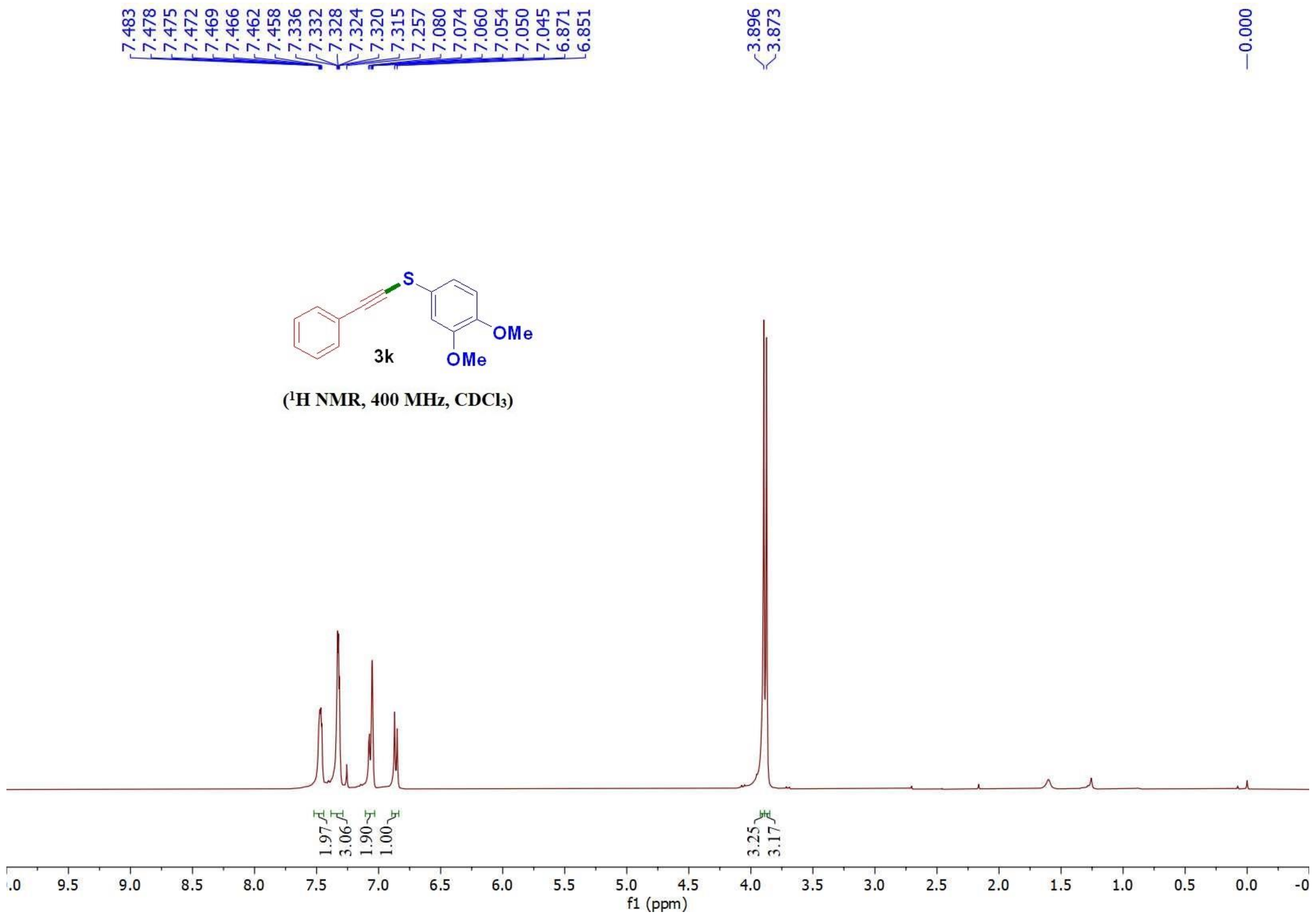
7.483
7.478
7.475
7.472
7.469
7.466
7.462
7.458
7.336
7.332
7.328
7.324
7.320
7.315
7.257
7.080
7.074
7.060
7.054
7.050
7.045
6.871
6.851
3.896
3.873
0.000
S
OMe
OMe
3k
(1H NMR, 400 MHz, CDCl3)
1.97
3.06
1.90
1.00
3.25
3.17
9.5
9.0
8.5
8.0
7.5
7.0
6.5
6.0
5.5
5.0
4.5
4.0
3.5
3.0
2.5
2.0
1.5
1.0
0.5
0.0
f1 (ppm)

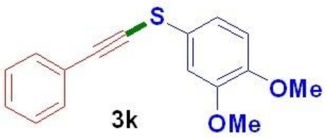

3k

($^{13}C\{H\}$ NMR, 100 MHz, $CDCl_3$)

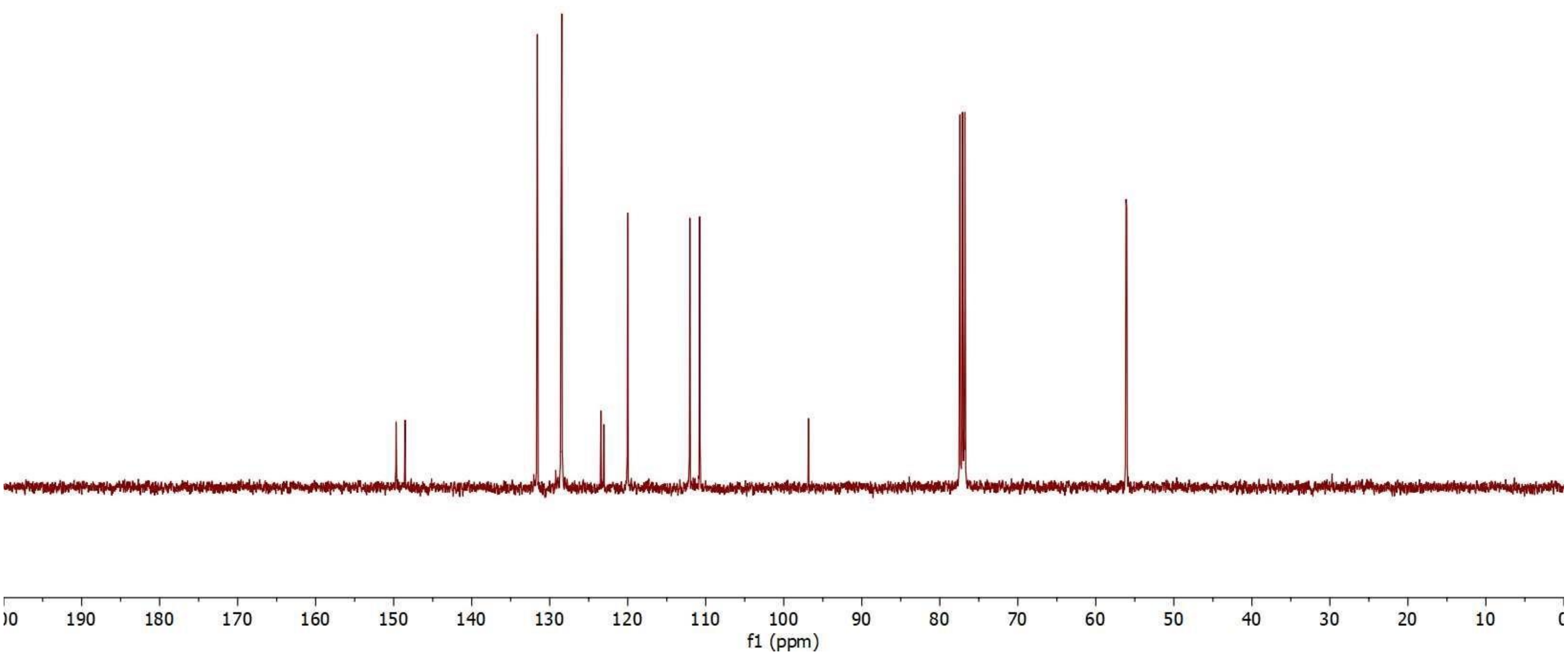

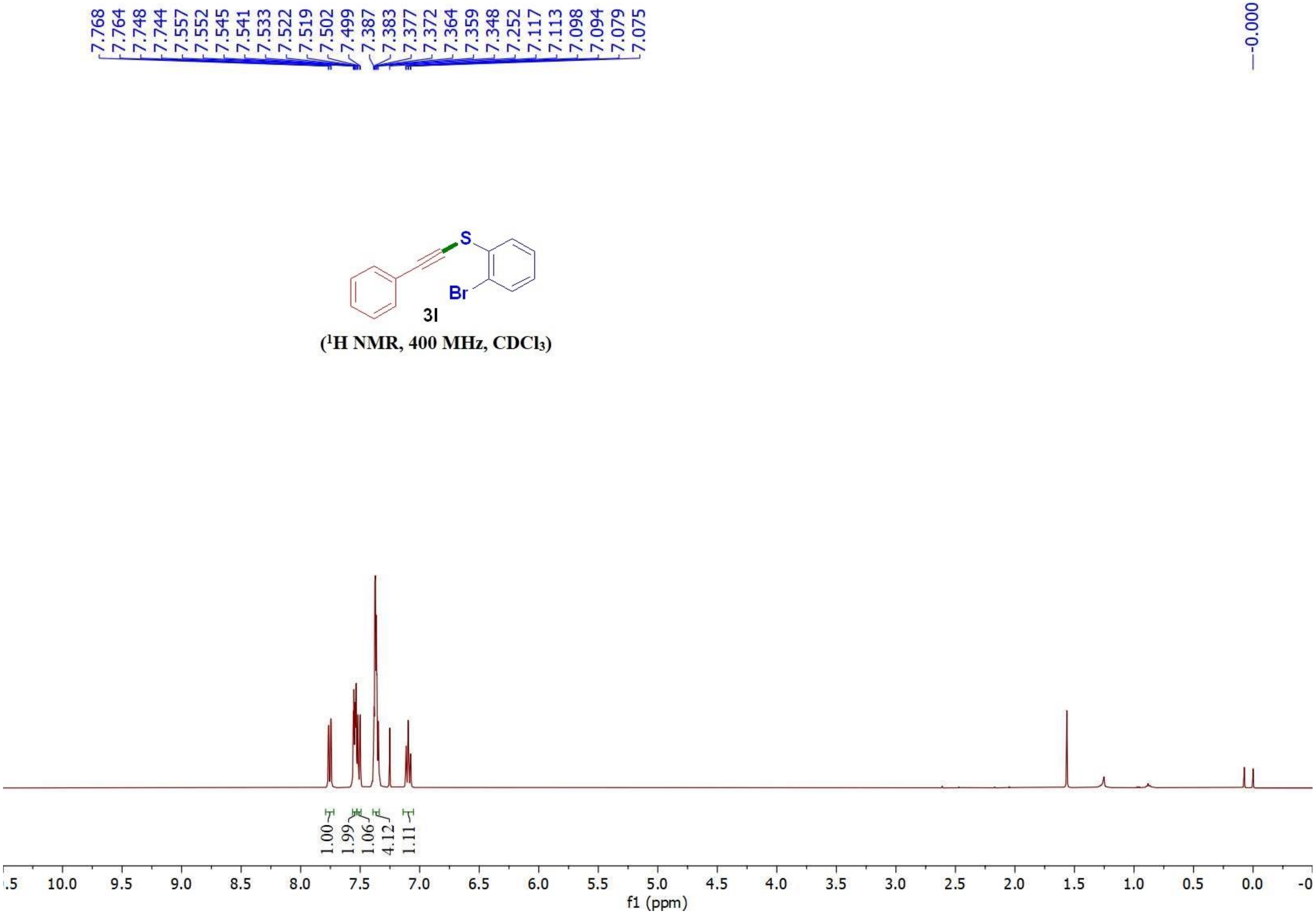
7.768
7.764
7.748
7.744
7.557
7.552
7.545
7.541
7.533
7.522
7.519
7.502
7.499
7.387
7.383
7.377
7.372
7.364
7.359
7.348
7.252
7.117
7.113
7.098
7.094
7.079
7.075
-0.000
S
Br
3l
(^{1}H NMR, 400 MHz, $CDCl_3$)
1.00
1.99
1.06
4.12
1.11
10.0
9.5
9.0
8.5
8.0
7.5
7.0
6.5
6.0
5.5
5.0
4.5
4.0
3.5
3.0
2.5
2.0
1.5
1.0
0.5
0.0
f1 (ppm)

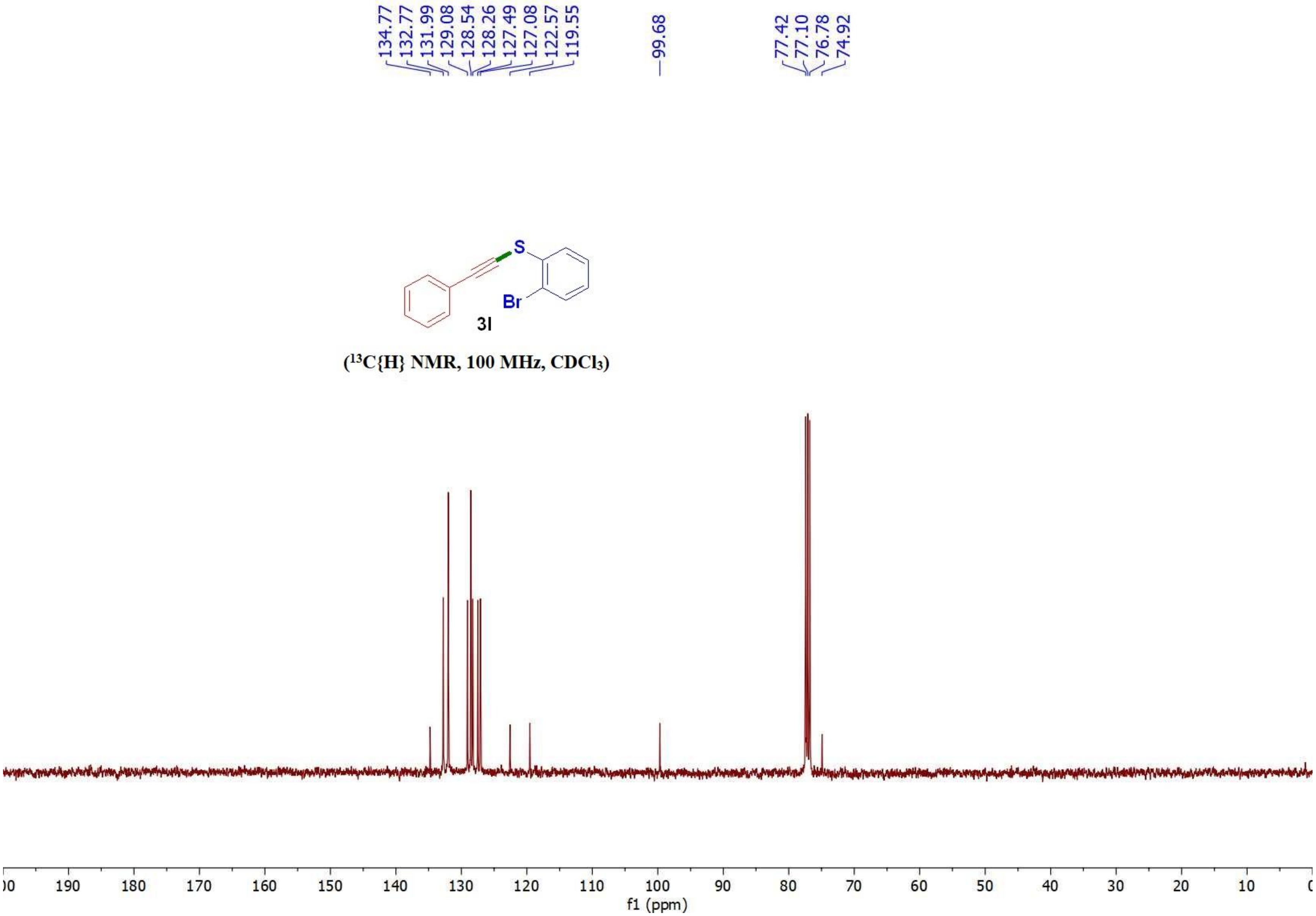

($^{13}C\{H\}$ NMR, 100 MHz, $CDCl_3$)

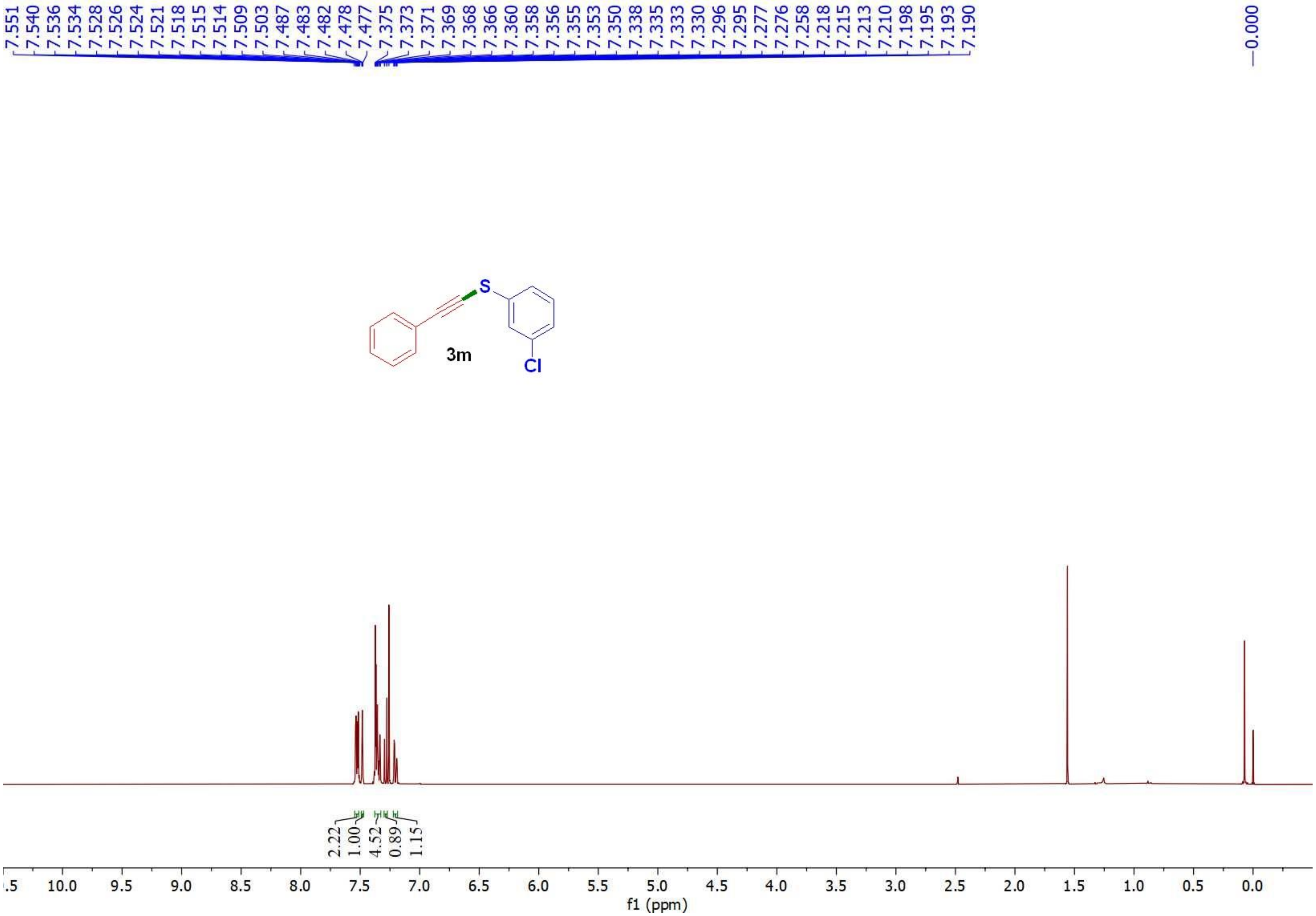
7.551 7.540 7.536 7.534 7.528 7.526 7.524 7.521 7.518 7.515 7.514 7.509 7.503 7.487 7.483 7.482 7.478 7.477 7.375 7.373 7.371 7.369 7.368 7.366 7.360 7.358 7.356 7.355 7.353 7.350 7.338 7.335 7.333 7.330 7.296 7.295 7.277 7.276 7.258 7.218 7.215 7.213 7.210 7.198 7.195 7.193 7.190
0.000
S
Cl
3m
2.22
1.00
4.52
0.89
1.15
f1 (ppm)

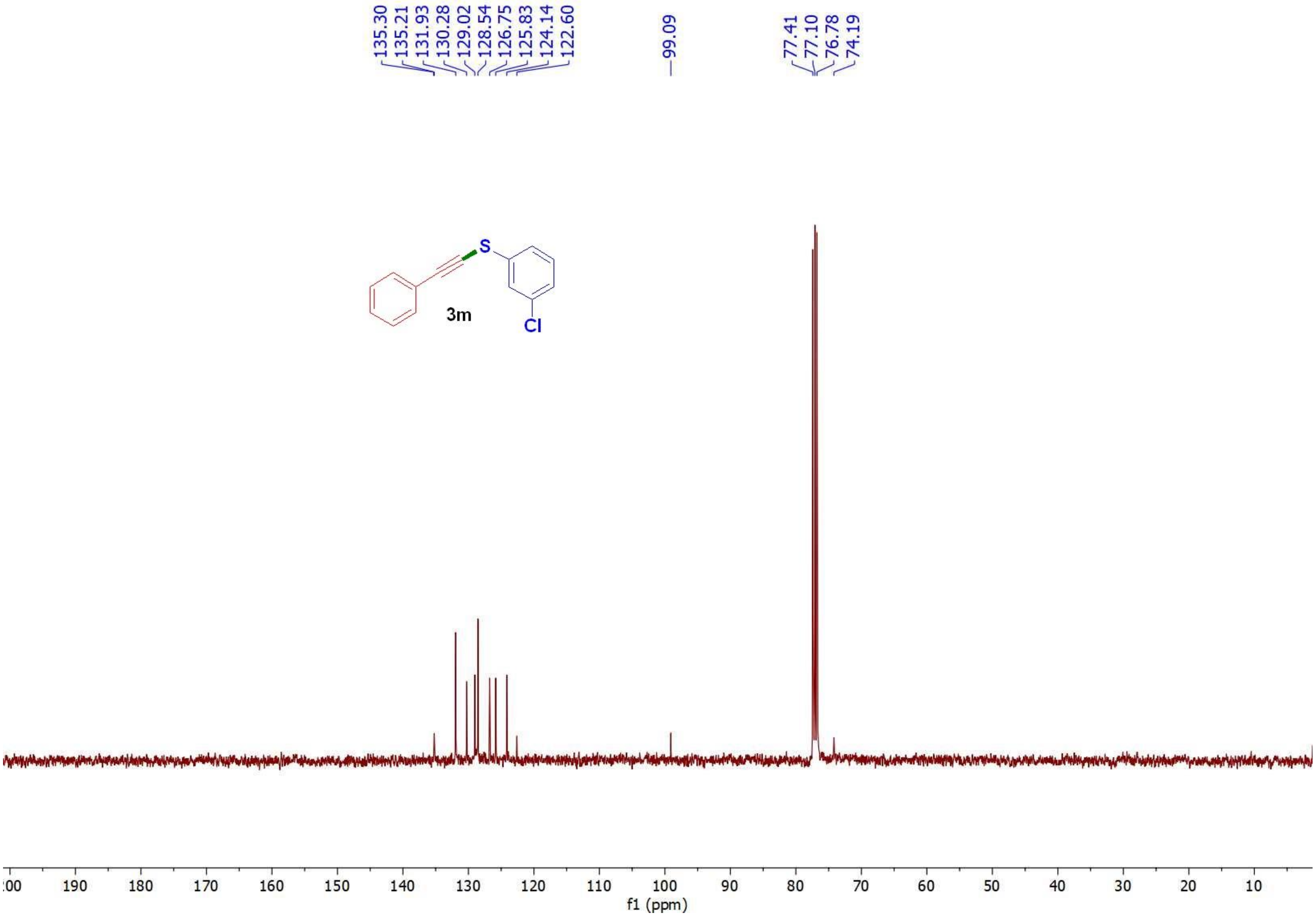

135.30
135.21
131.93
130.28
129.02
128.54
126.75
125.83
124.14
122.60
99.09
77.41
77.10
76.78
74.19
S
3m
Cl
200
190
180
170
160
150
140
130
120
110
100
90
80
70
60
50
40
30
20
10
f1 (ppm)

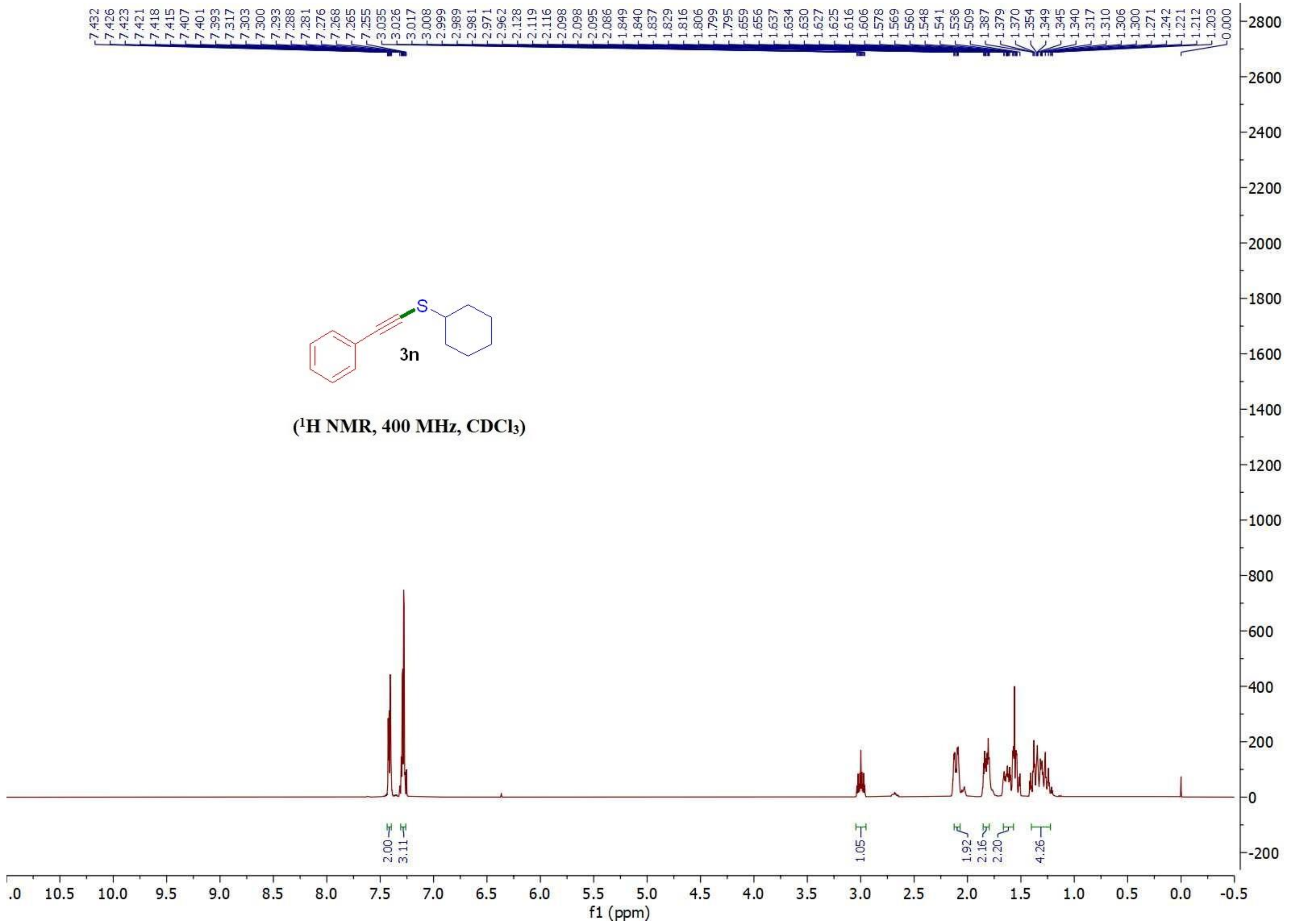
3n
(¹H NMR, 400 MHz, CDCl₃)
f1 (ppm)

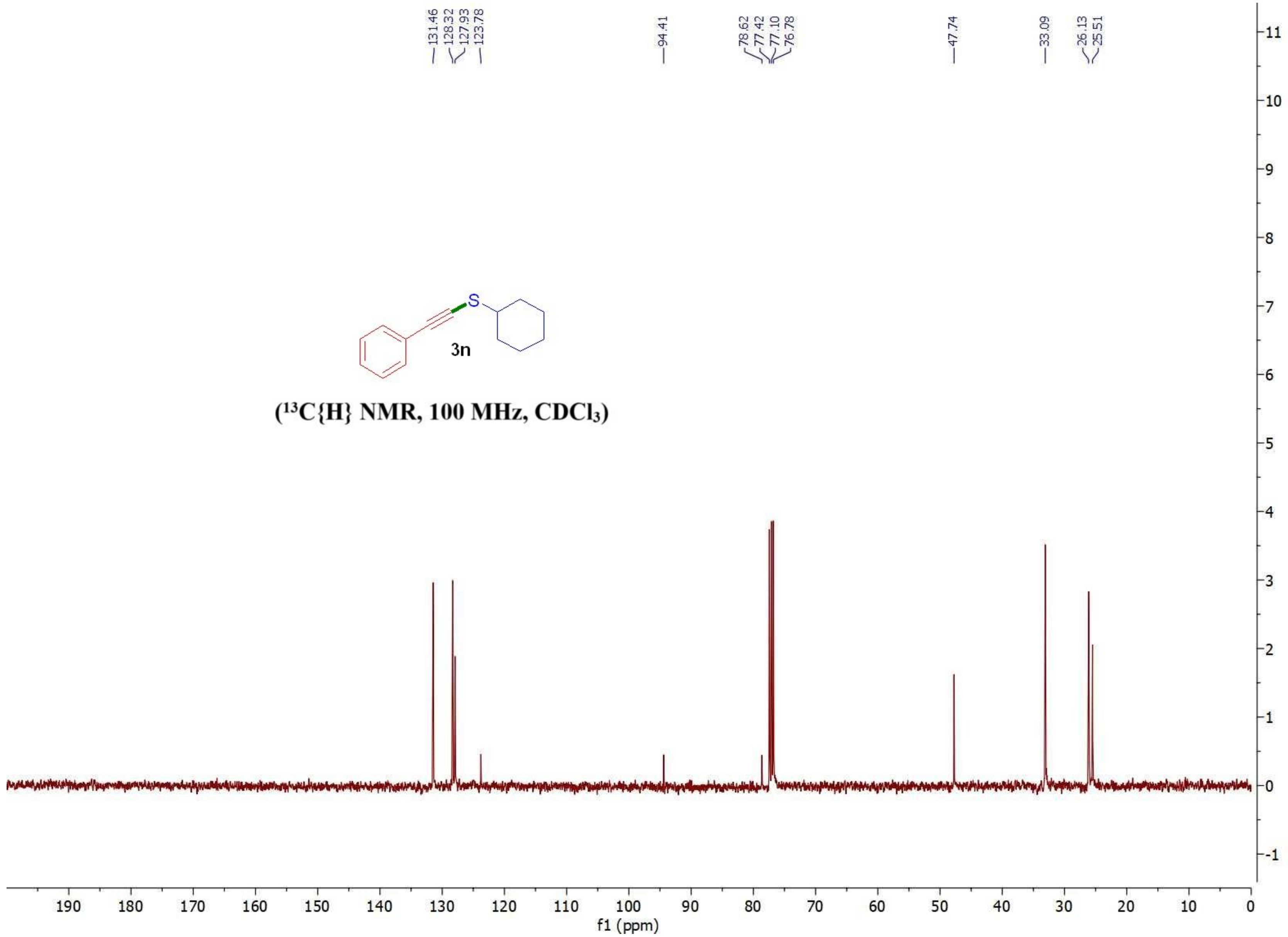

131.46
128.32
127.93
123.78
94.41
78.62
77.42
77.10
76.78
47.74
33.09
26.13
25.51
S
3n
(13C{H} NMR, 100 MHz, CDCl3)
f1 (ppm)

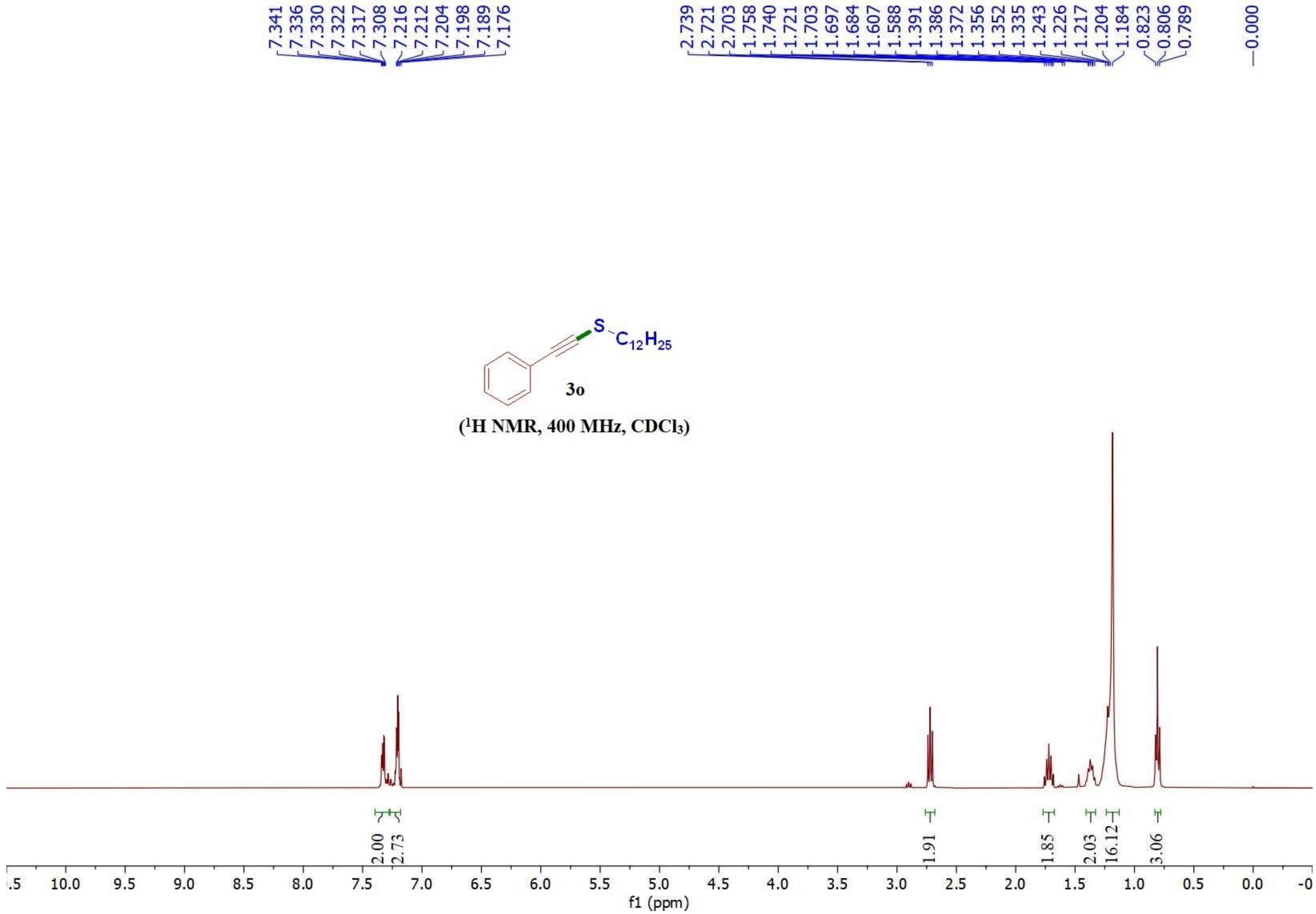

3o

(^{1}H NMR, 400 MHz, $CDCl_3$)

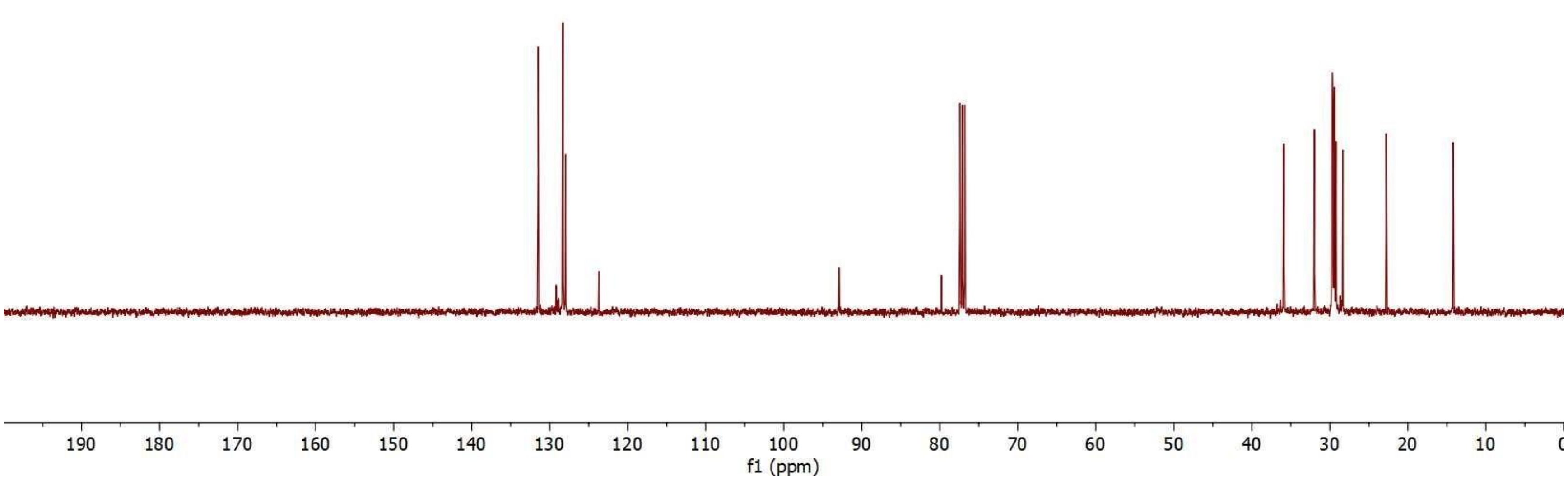

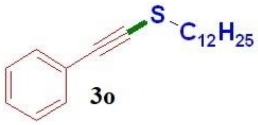

3o

($^{13}C\{H\}$ NMR, 100 MHz, $CDCl_3$)

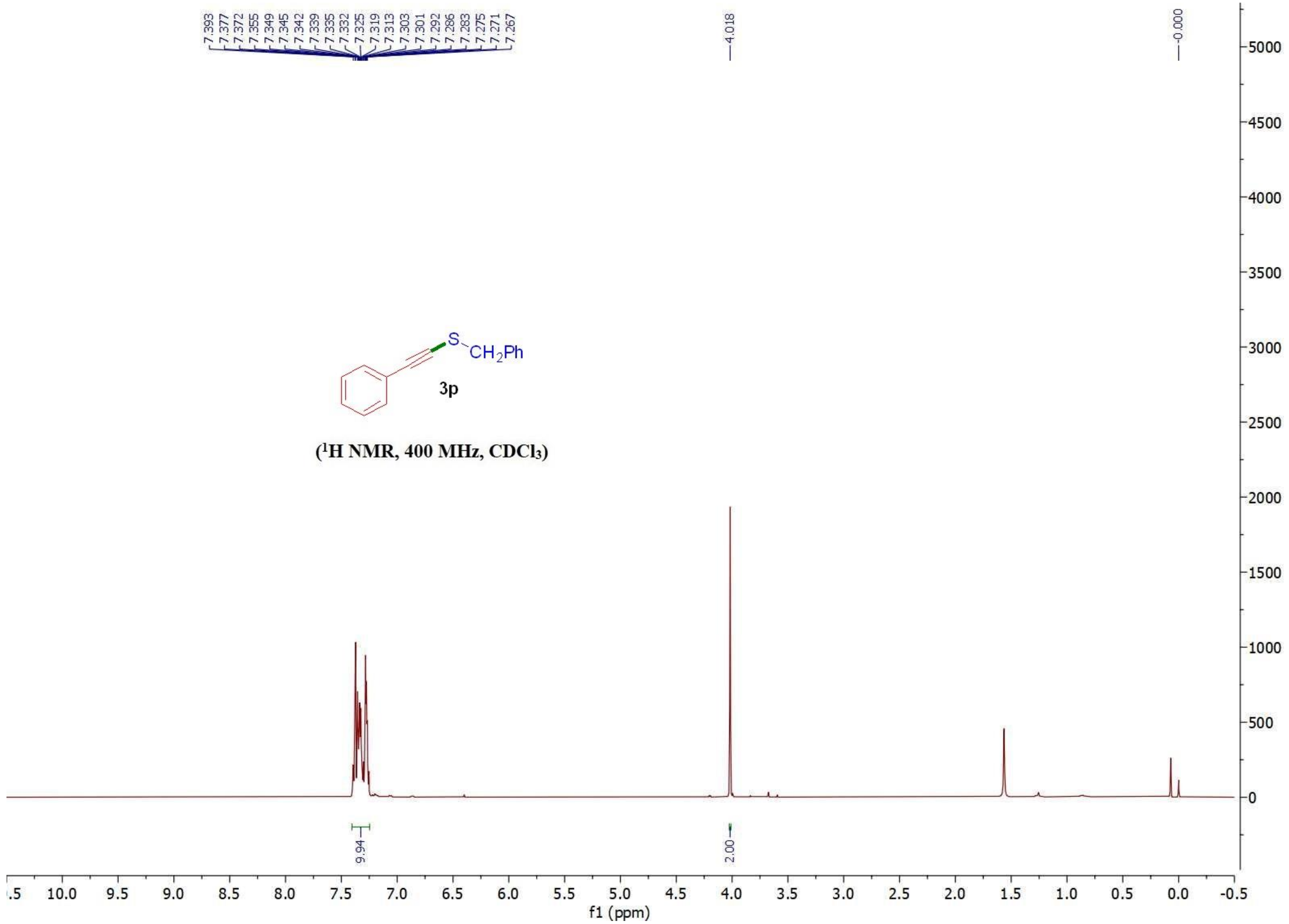
7.393
7.377
7.372
7.355
7.349
7.345
7.342
7.339
7.335
7.332
7.325
7.319
7.313
7.303
7.301
7.292
7.286
7.283
7.275
7.271
7.267
4.018
0.000
S
CH2Ph
3p
(1H NMR, 400 MHz, CDCl3)
9.94
2.00
f1 (ppm)

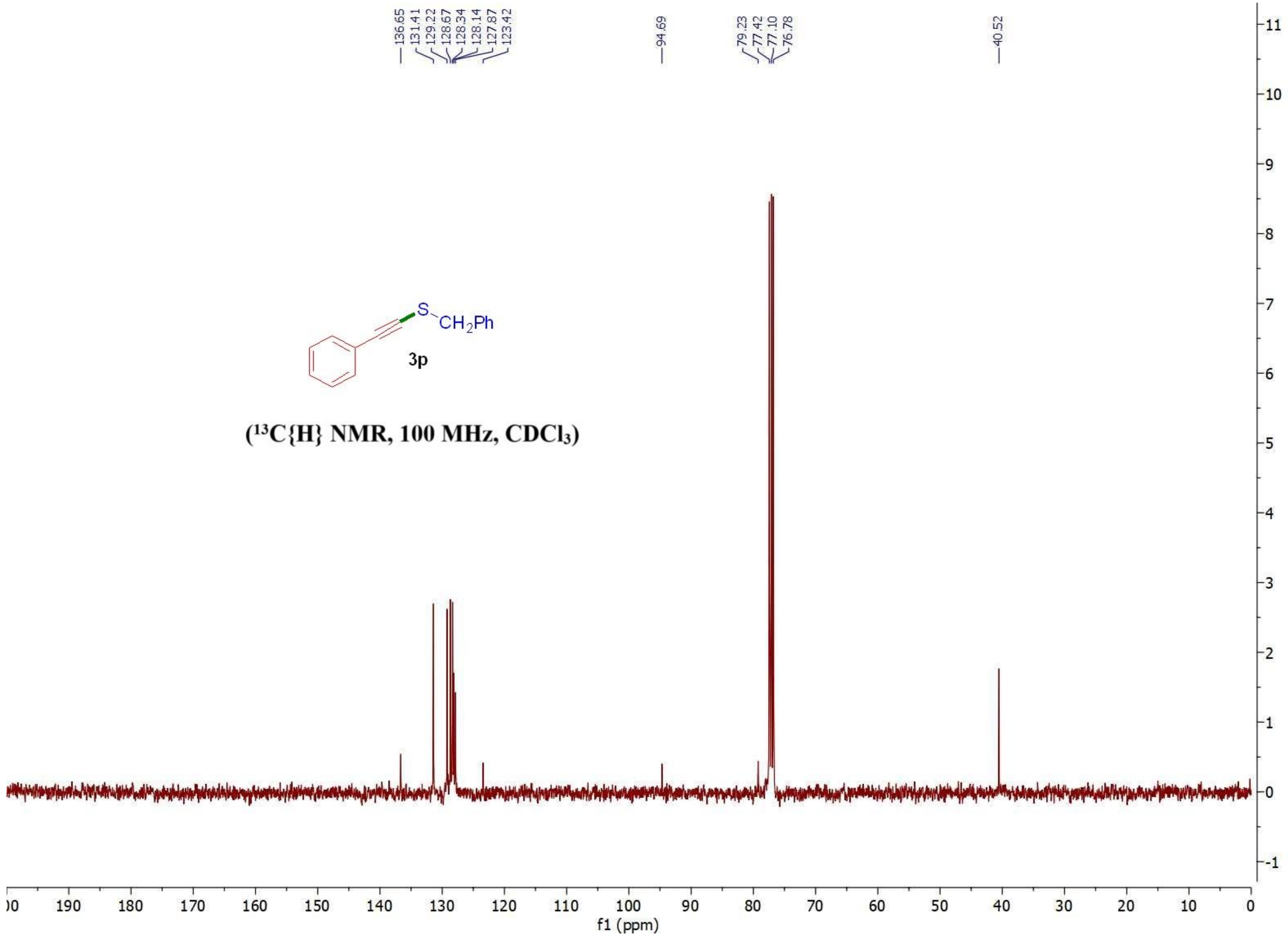
136.65
131.41
129.22
128.67
128.34
128.14
127.87
123.42
94.69
79.23
77.42
77.10
76.78
40.52
S
CH2Ph
3p
(13C{H} NMR, 100 MHz, CDCl3)
f1 (ppm)

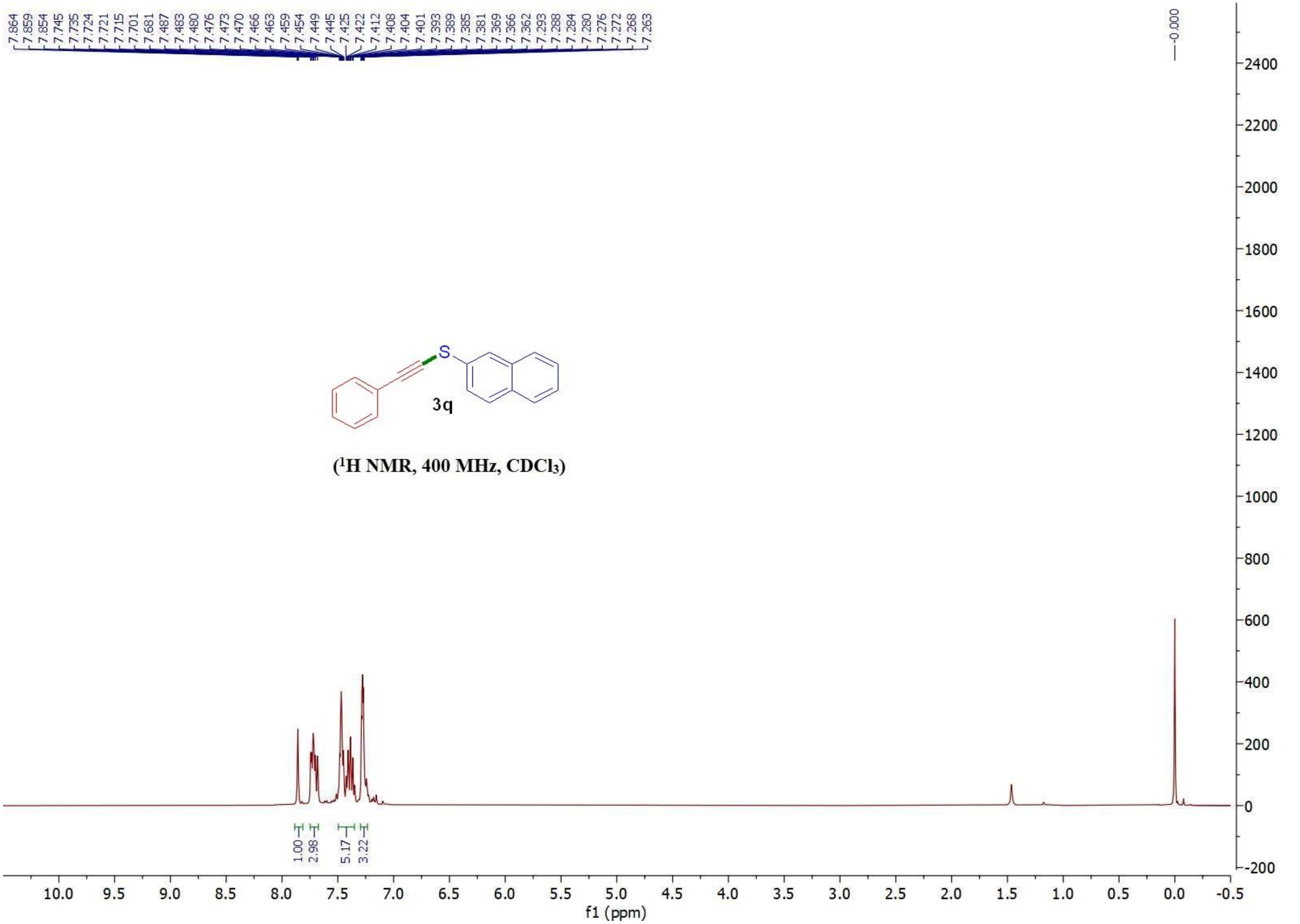

(1H NMR, 400 MHz, CDCl3)

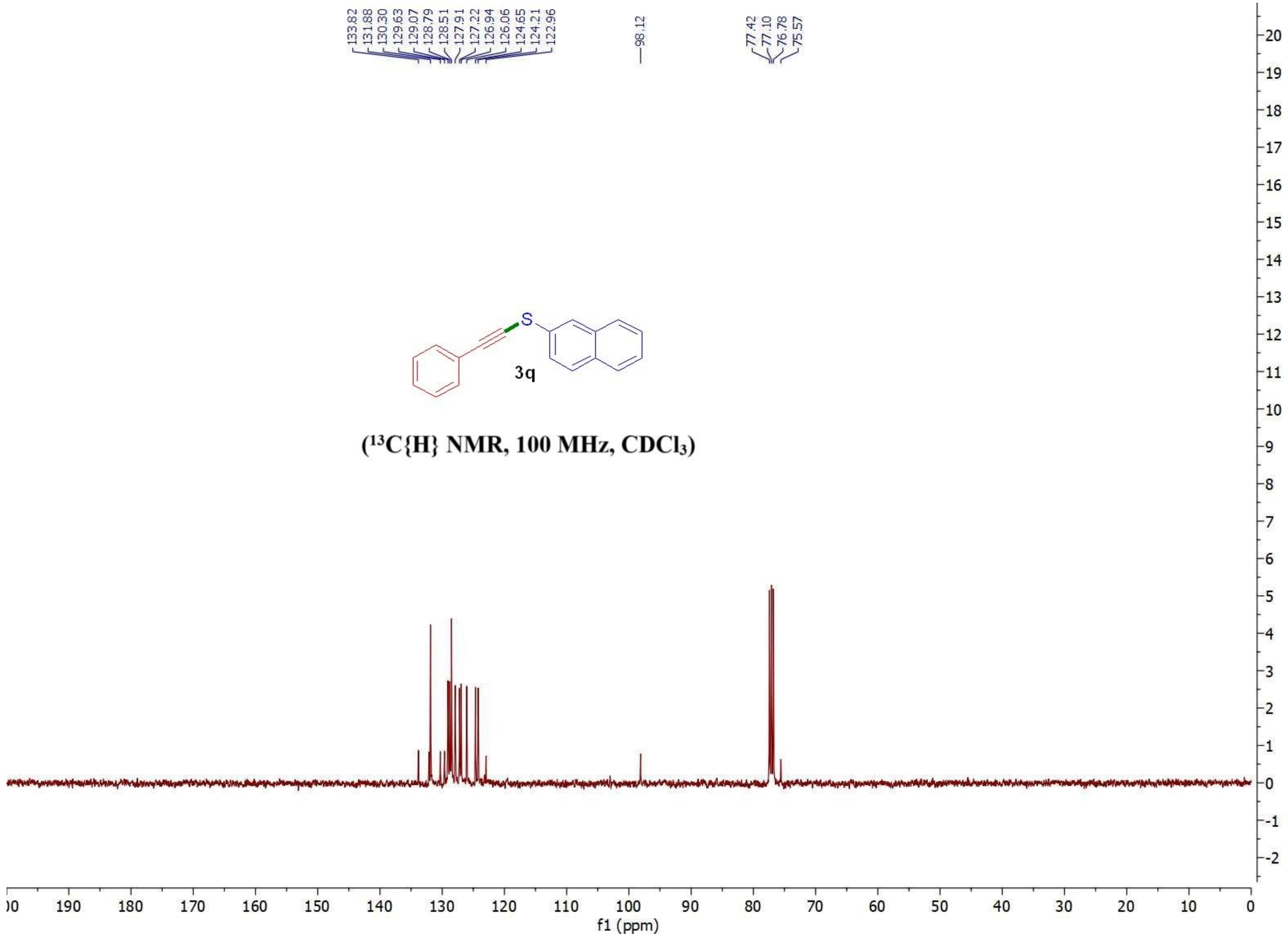

133.82
131.88
130.30
129.63
129.07
128.79
128.51
127.91
127.22
126.94
126.06
124.65
124.21
122.96
98.12
77.42
77.10
76.78
75.57
S
3q
(13C{H} NMR, 100 MHz, CDCl3)
f1 (ppm)

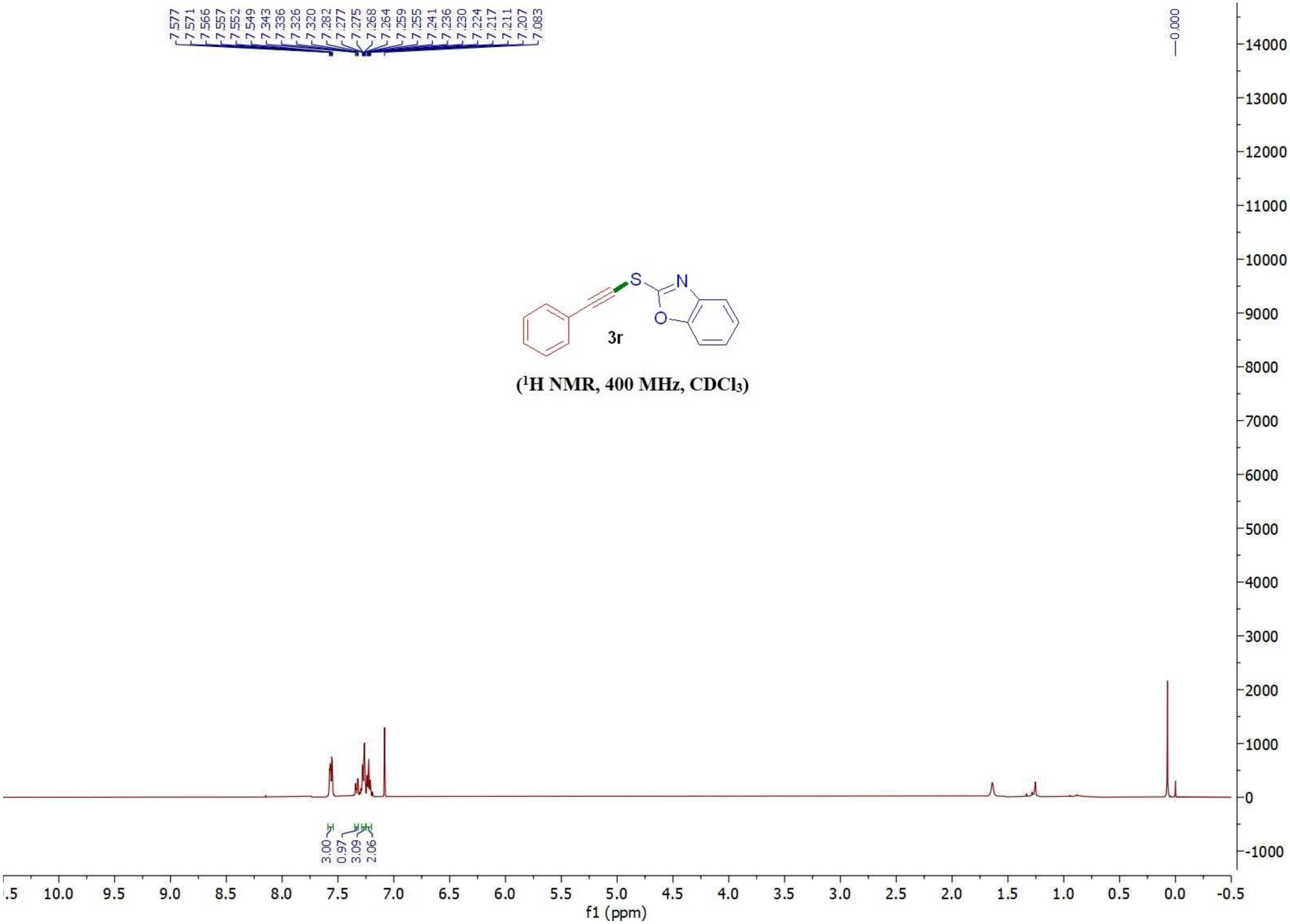
7.577 7.571 7.566 7.557 7.552 7.549 7.343 7.336 7.326 7.320 7.282 7.277 7.275 7.268 7.264 7.259 7.255 7.241 7.236 7.230 7.224 7.217 7.211 7.207 7.083
0.000
S
N
O
3r
(1H NMR, 400 MHz, CDCl3)
3.00 0.97 3.09 2.06
f1 (ppm)

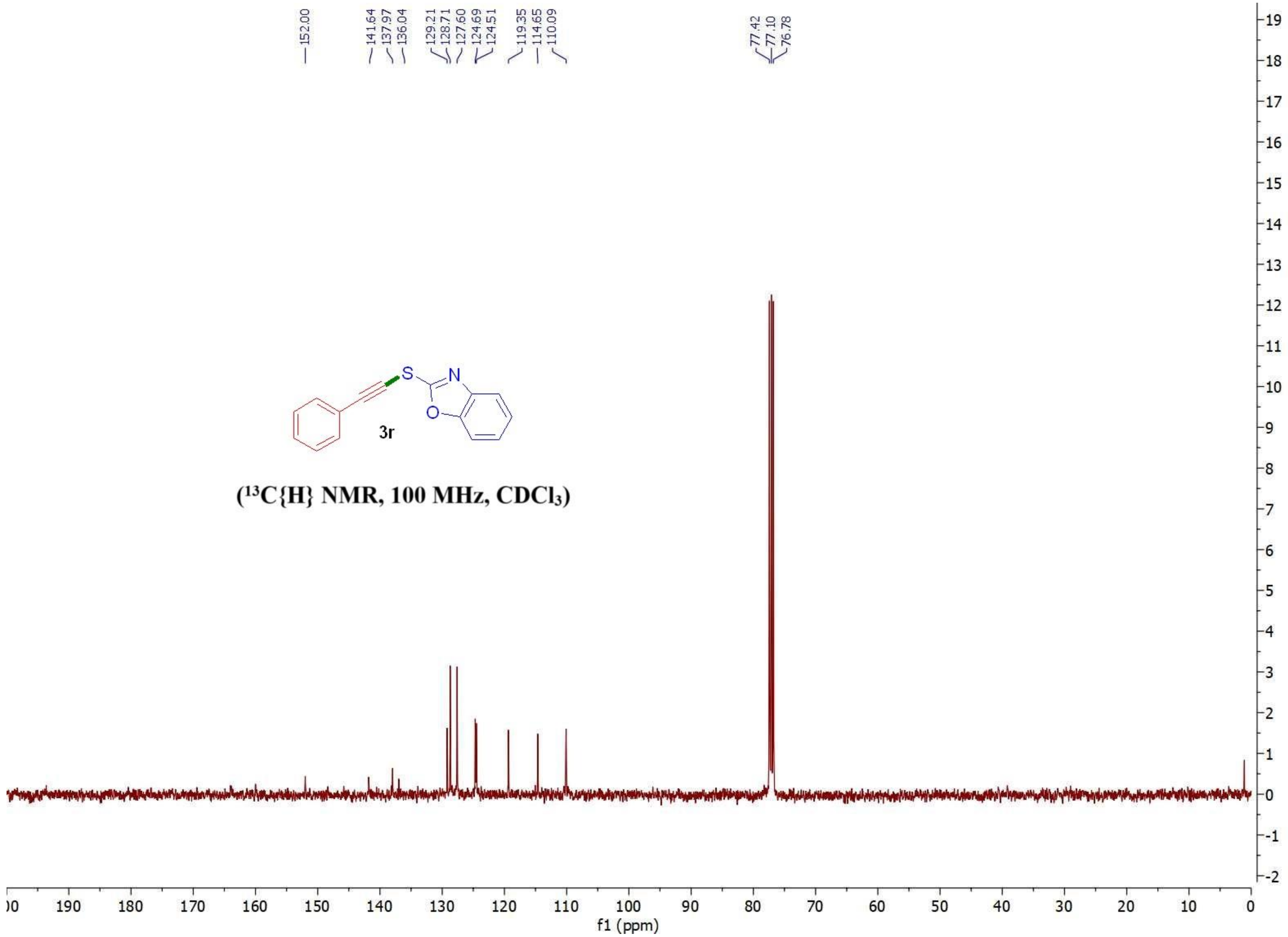

(¹³C{H} NMR, 100 MHz, $CDCl_3$)

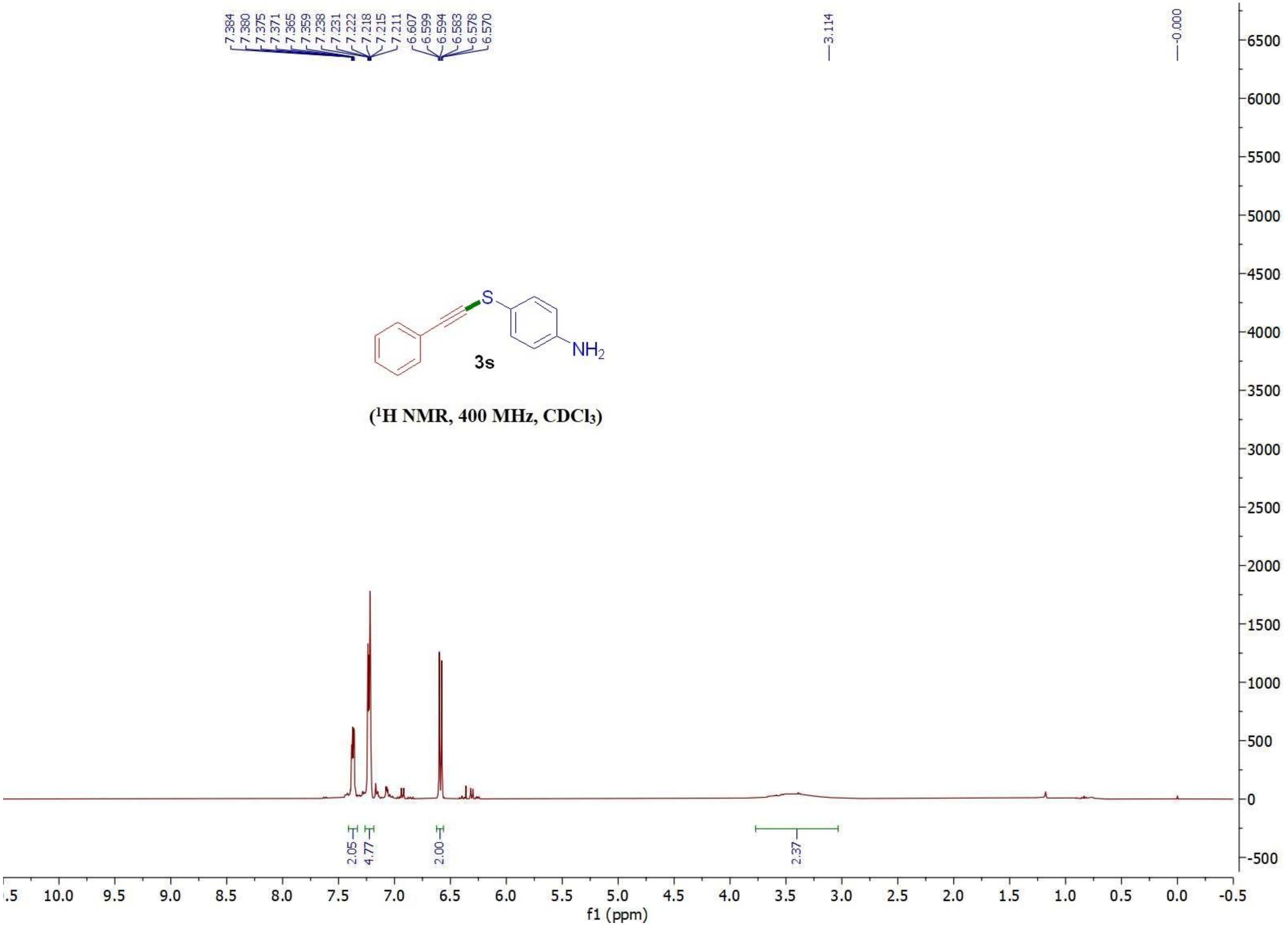
7.384
7.380
7.375
7.371
7.365
7.359
7.238
7.231
7.222
7.218
7.215
7.211
6.607
6.599
6.594
6.583
6.578
6.570
3.114
0.000
S
NH2
3s
(1H NMR, 400 MHz, CDCl3)
2.05
4.77
2.00
2.37
f1 (ppm)

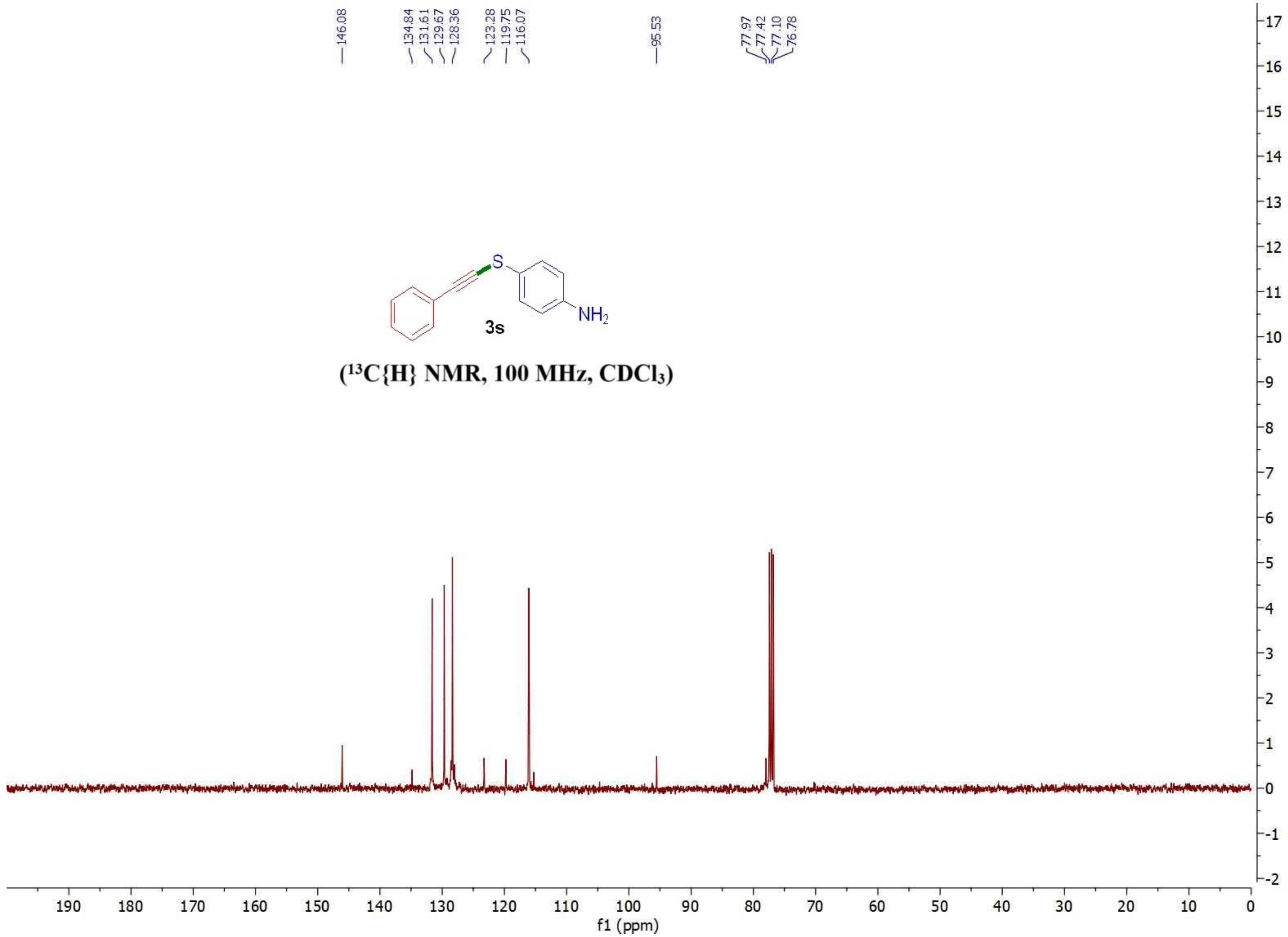
146.08
134.84
131.61
129.67
128.36
123.28
119.75
116.07
95.53
77.97
77.42
77.10
76.78
S
NH2
3s
(13C{H} NMR, 100 MHz, CDCl3)
f1 (ppm)

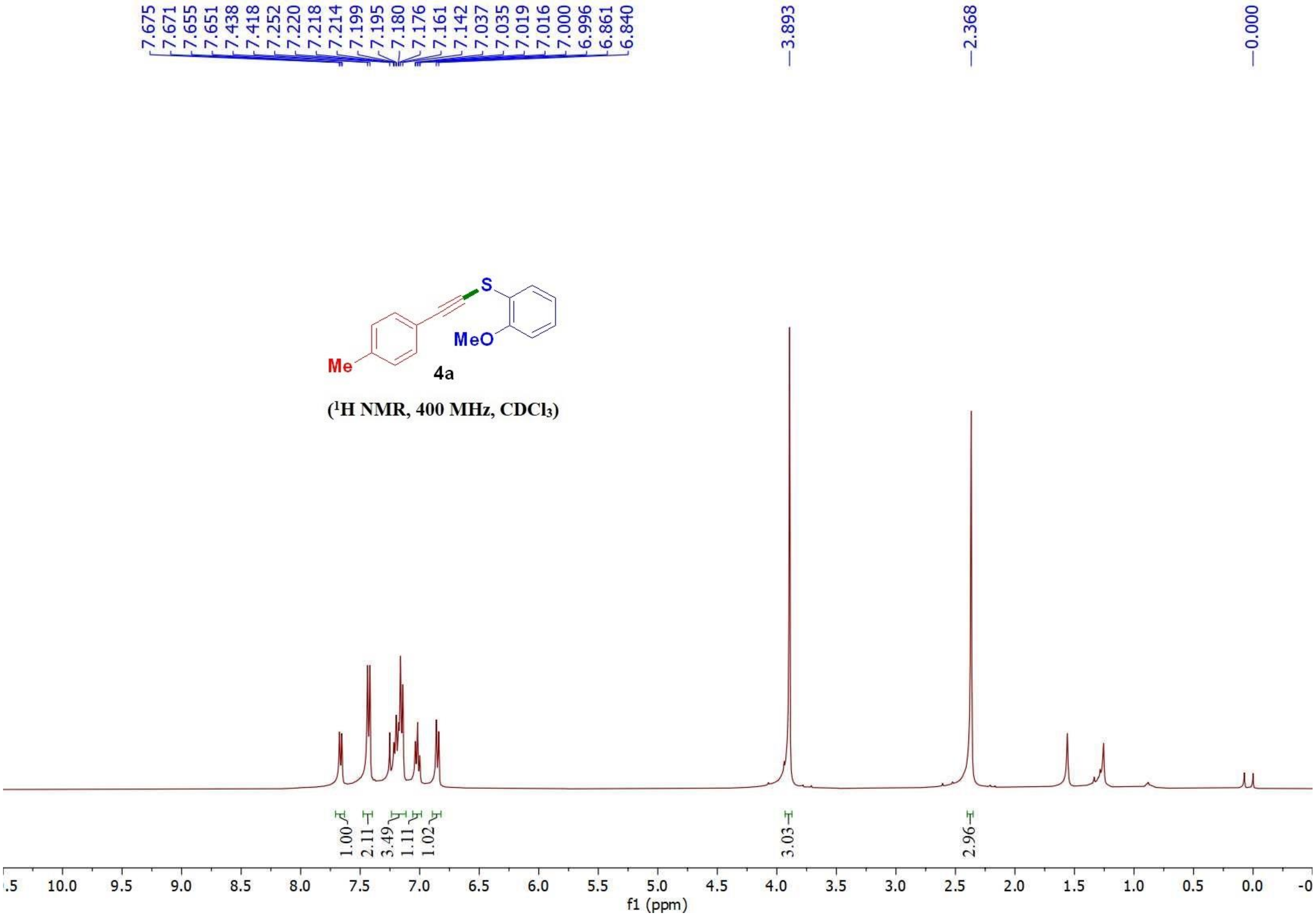
7.675
7.671
7.655
7.651
7.438
7.418
7.252
7.220
7.218
7.214
7.199
7.195
7.180
7.176
7.161
7.142
7.037
7.035
7.019
7.016
7.000
6.996
6.861
6.840
3.893
2.368
0.000
S
MeO
Me
4a
(1H NMR, 400 MHz, CDCl3)
1.00
2.11
3.49
1.11
1.02
3.03
2.96
f1 (ppm)

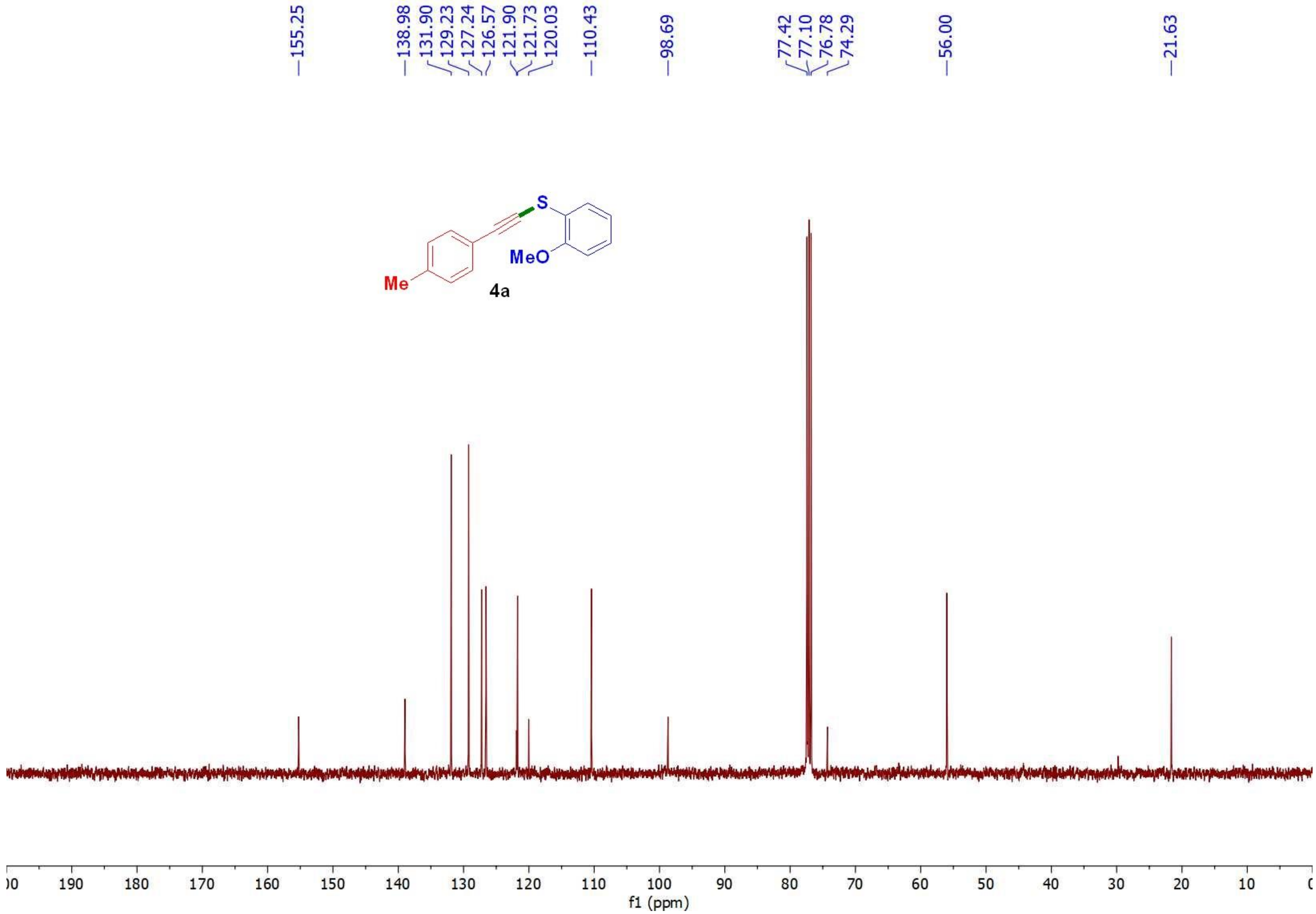

155.25
138.98
131.90
129.23
127.24
126.57
121.90
121.73
120.03
110.43
98.69
77.42
77.10
76.78
74.29
56.00
21.63
S
MeO
Me
4a
f1 (ppm)

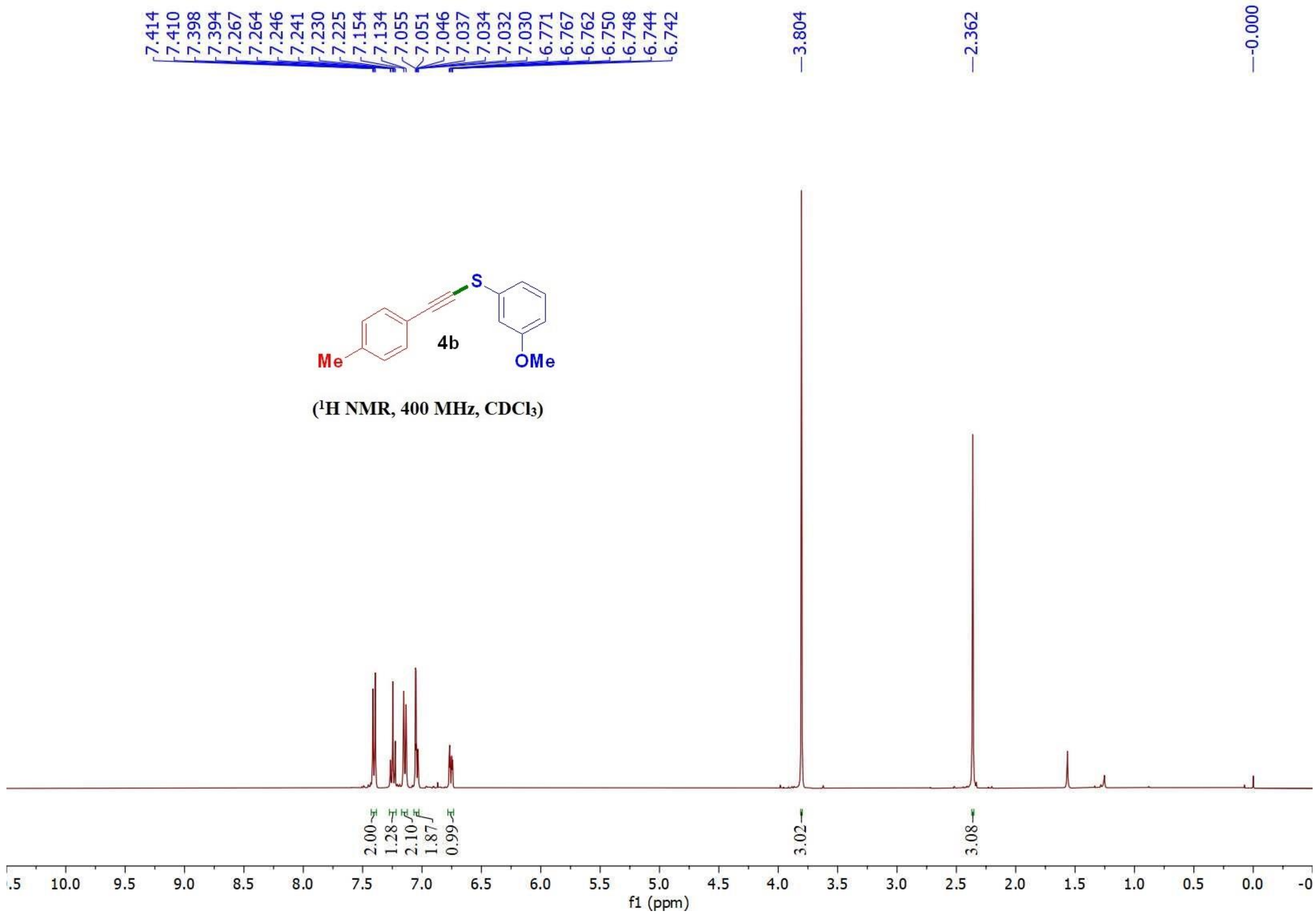
7.414
7.410
7.398
7.394
7.267
7.264
7.246
7.241
7.230
7.225
7.154
7.134
7.055
7.051
7.046
7.037
7.034
7.032
7.030
6.771
6.767
6.762
6.750
6.748
6.744
6.742
3.804
2.362
-0.000
Me
S
OMe
4b
(¹H NMR, 400 MHz, CDCl₃)
2.00
1.28
2.10
1.87
0.99
3.02
3.08
f1 (ppm)

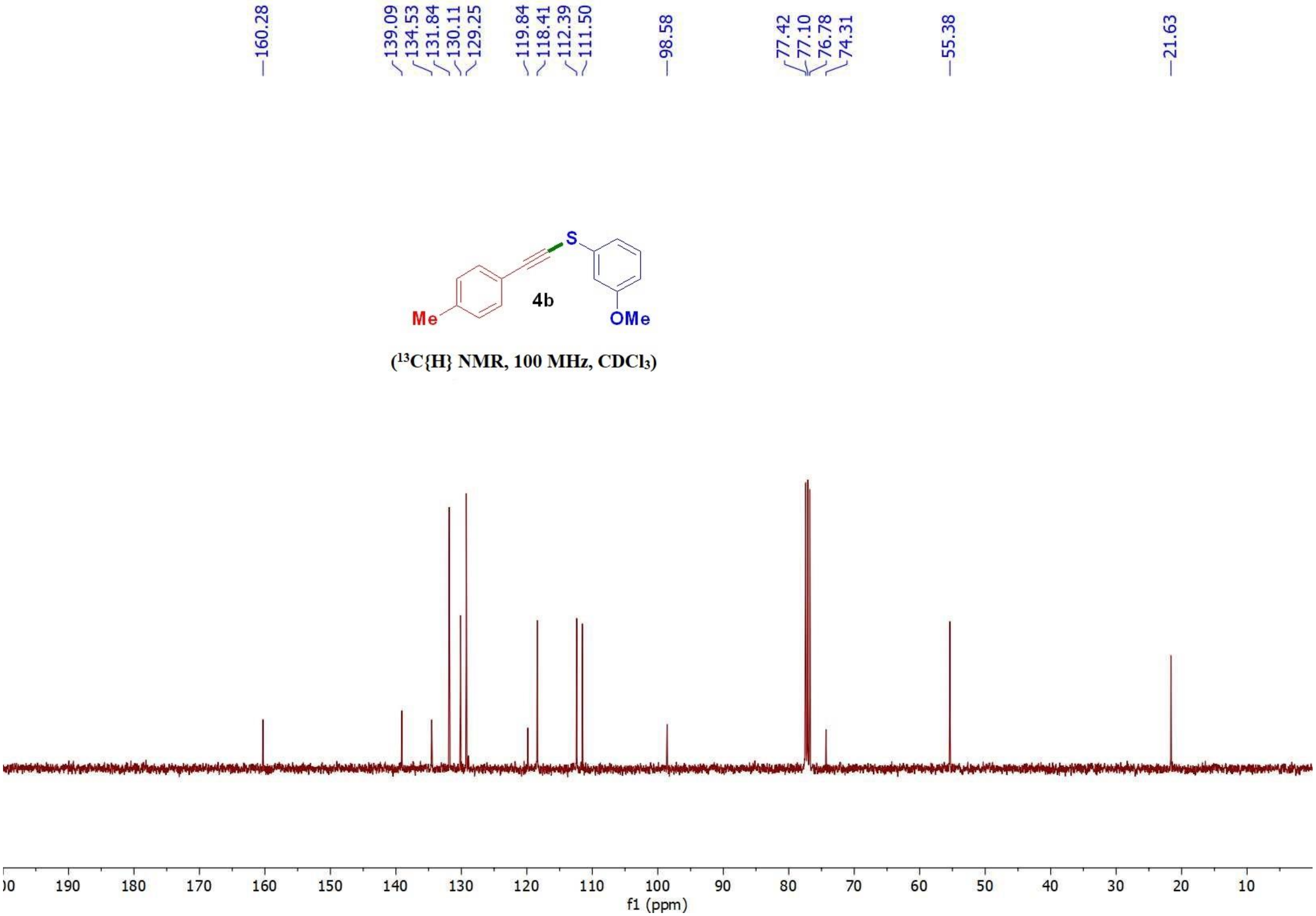

(${}^{13}C\{H\}$ NMR, 100 MHz, $CDCl_3$)

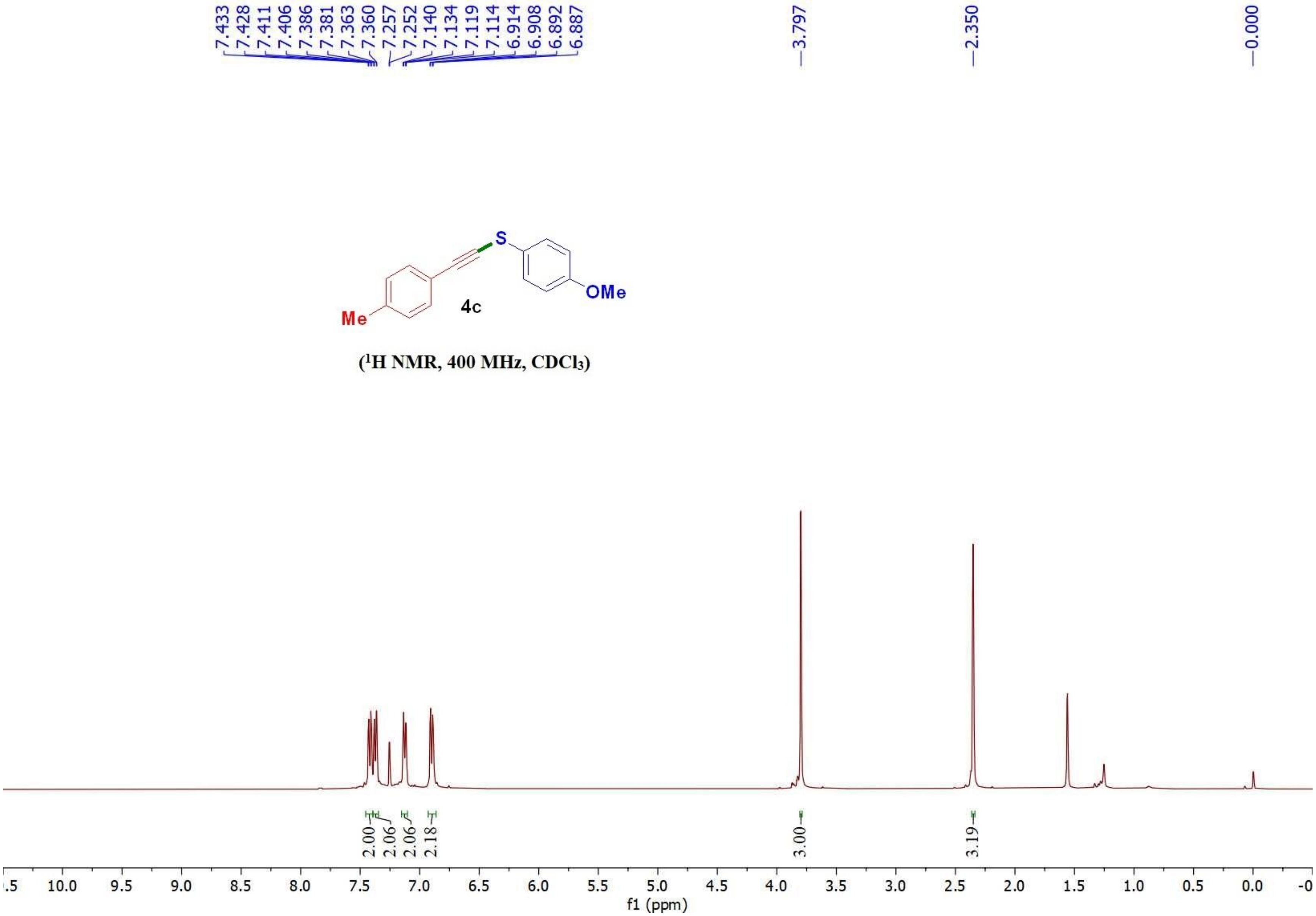
7.433
7.428
7.411
7.406
7.386
7.381
7.363
7.360
7.257
7.252
7.140
7.134
7.119
7.114
6.914
6.908
6.892
6.887
3.797
2.350
0.000
S
OMe
Me
4c
(¹H NMR, 400 MHz, CDCl₃)
2.00
2.06
2.06
2.18
3.00
3.19
f1 (ppm)

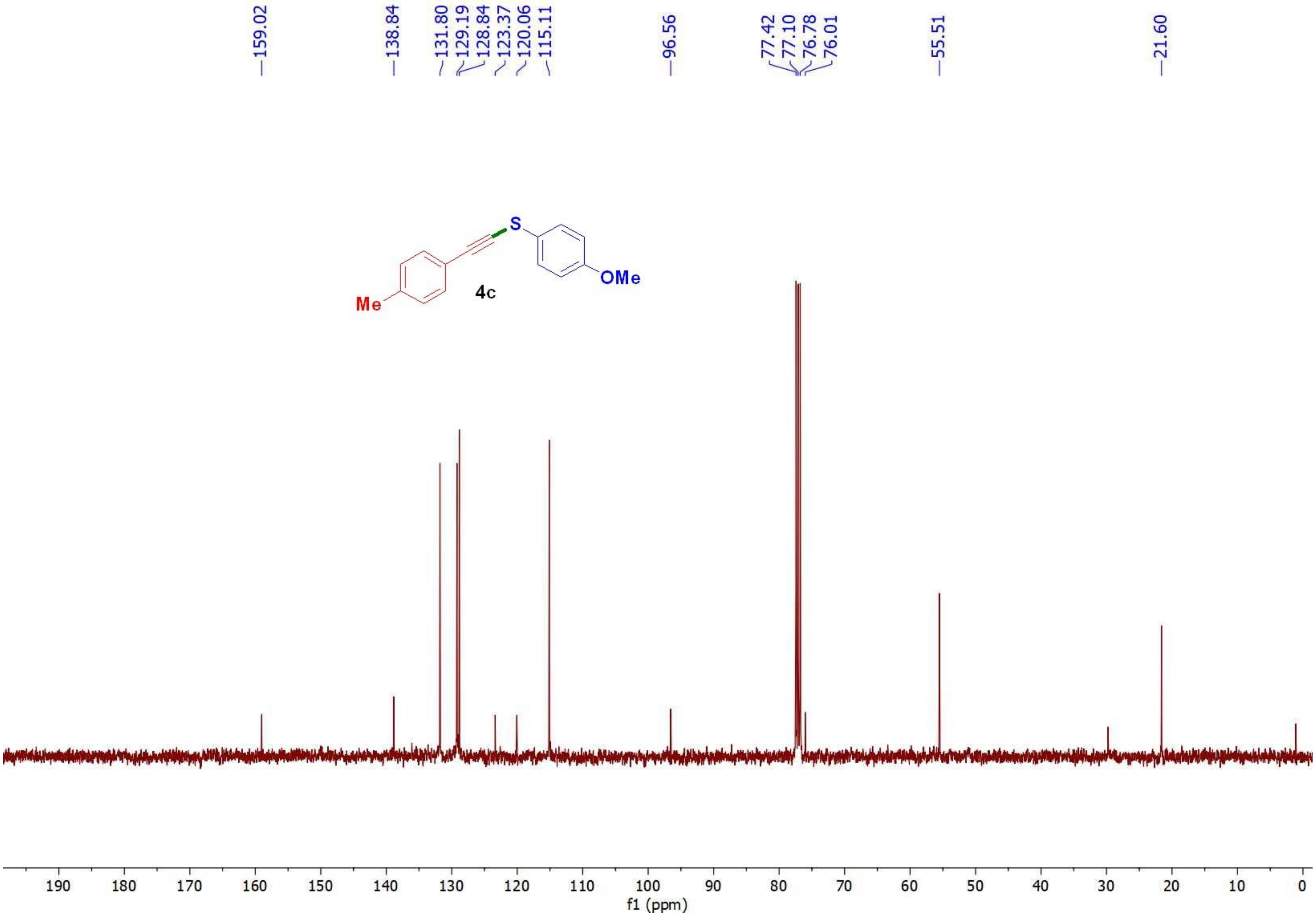
159.02
138.84
131.80
129.19
128.84
123.37
120.06
115.11
96.56
77.42
77.10
76.78
76.01
55.51
21.60
S
OMe
Me
4c
190 180 170 160 150 140 130 120 110 100 90 80 70 60 50 40 30 20 10 0
f1 (ppm)

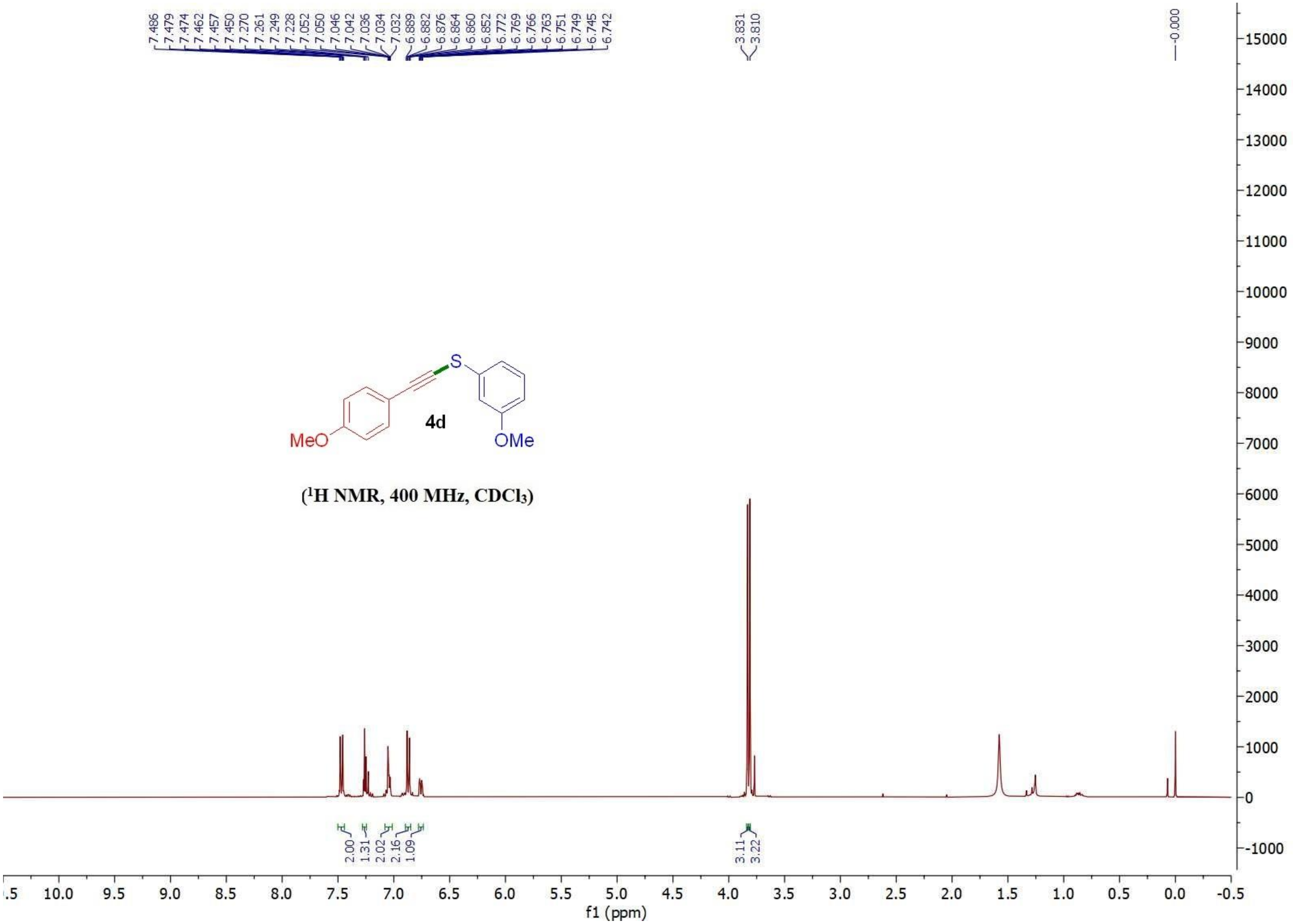

7.486
7.479
7.474
7.462
7.457
7.450
7.270
7.261
7.249
7.228
7.052
7.050
7.046
7.042
7.036
7.034
7.032
6.889
6.882
6.876
6.864
6.860
6.852
6.772
6.769
6.766
6.763
6.751
6.749
6.745
6.742
3.831
3.810
0.000
MeO
S
OMe
4d
(¹H NMR, 400 MHz, CDCl₃)
2.00
1.31
2.02
2.16
1.09
3.11
3.22
f1 (ppm)

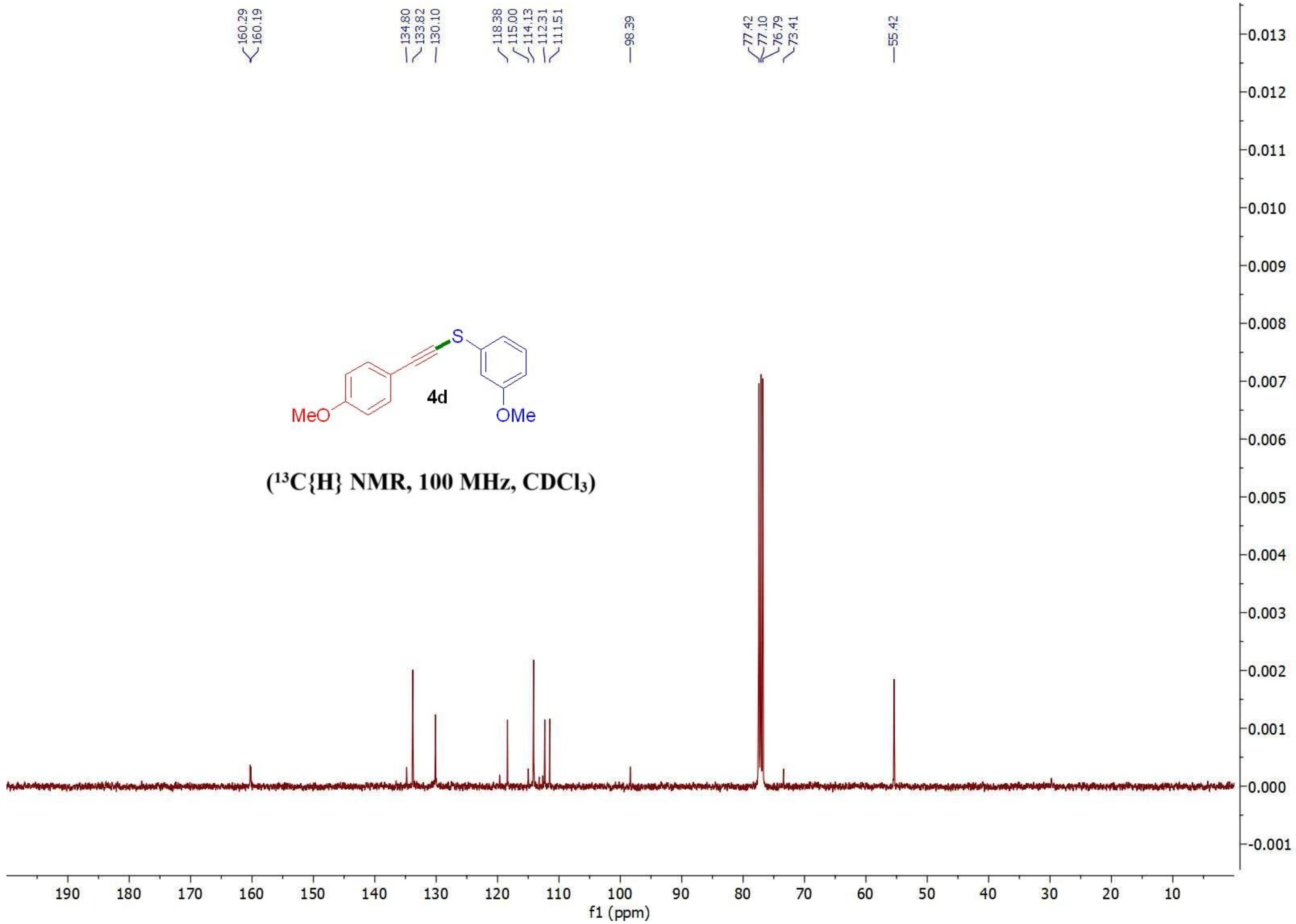
160.29
160.19
134.80
133.82
130.10
118.38
115.00
114.13
112.31
111.51
98.39
77.42
77.10
76.79
73.41
55.42
MeO
S
OMe
4d
(13C{H} NMR, 100 MHz, CDCl3)
f1 (ppm)

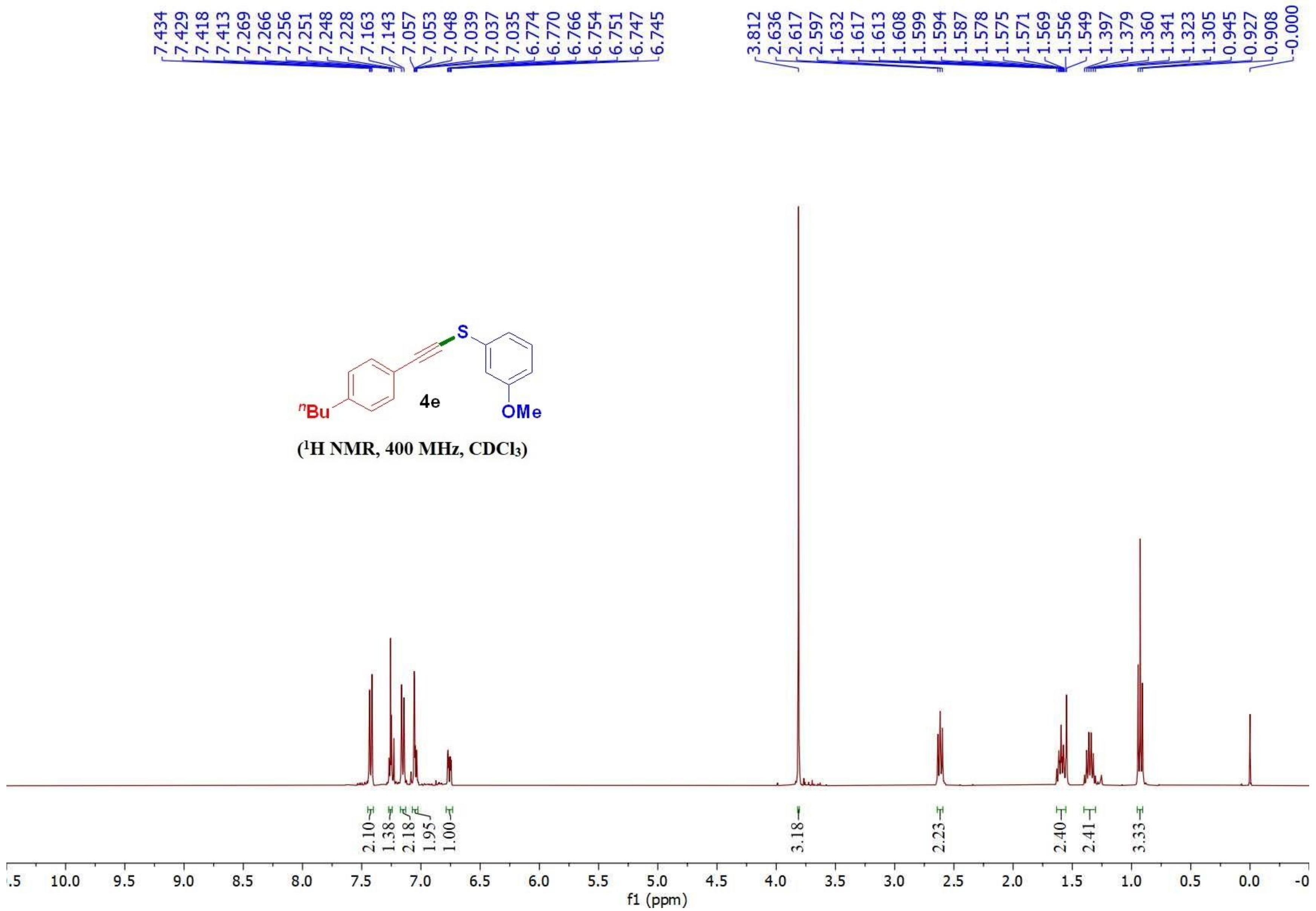
7.434 7.429 7.418 7.413 7.269 7.266 7.256 7.251 7.248 7.228 7.163 7.143 7.057 7.053 7.048 7.039 7.037 7.035 6.774 6.770 6.766 6.754 6.751 6.747 6.745
3.812 2.636 2.617 2.597 1.632 1.617 1.613 1.608 1.599 1.594 1.587 1.578 1.575 1.571 1.569 1.556 1.549 1.397 1.379 1.360 1.341 1.323 1.305 0.945 0.927 0.908 -0.000
S
^{n}Bu
4e
OMe
(^{1}H NMR, 400 MHz, $CDCl_3$)
2.10 1.38 2.18 1.95 1.00 3.18 2.23 2.40 2.41 3.33
.5 10.0 9.5 9.0 8.5 8.0 7.5 7.0 6.5 6.0 5.5 5.0 4.5 4.0 3.5 3.0 2.5 2.0 1.5 1.0 0.5 0.0 -0
f1 (ppm)

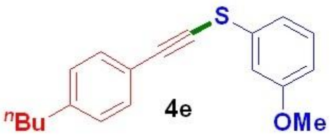

4e

(^{13}C{H} NMR, 100 MHz, CDCl$_3$)

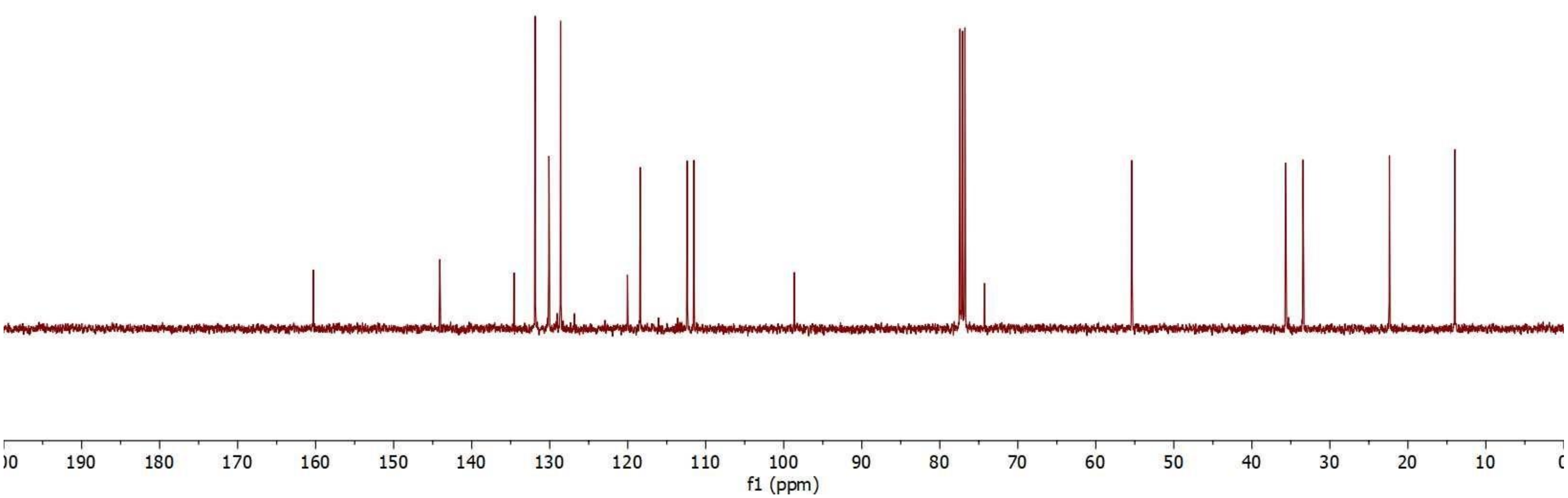

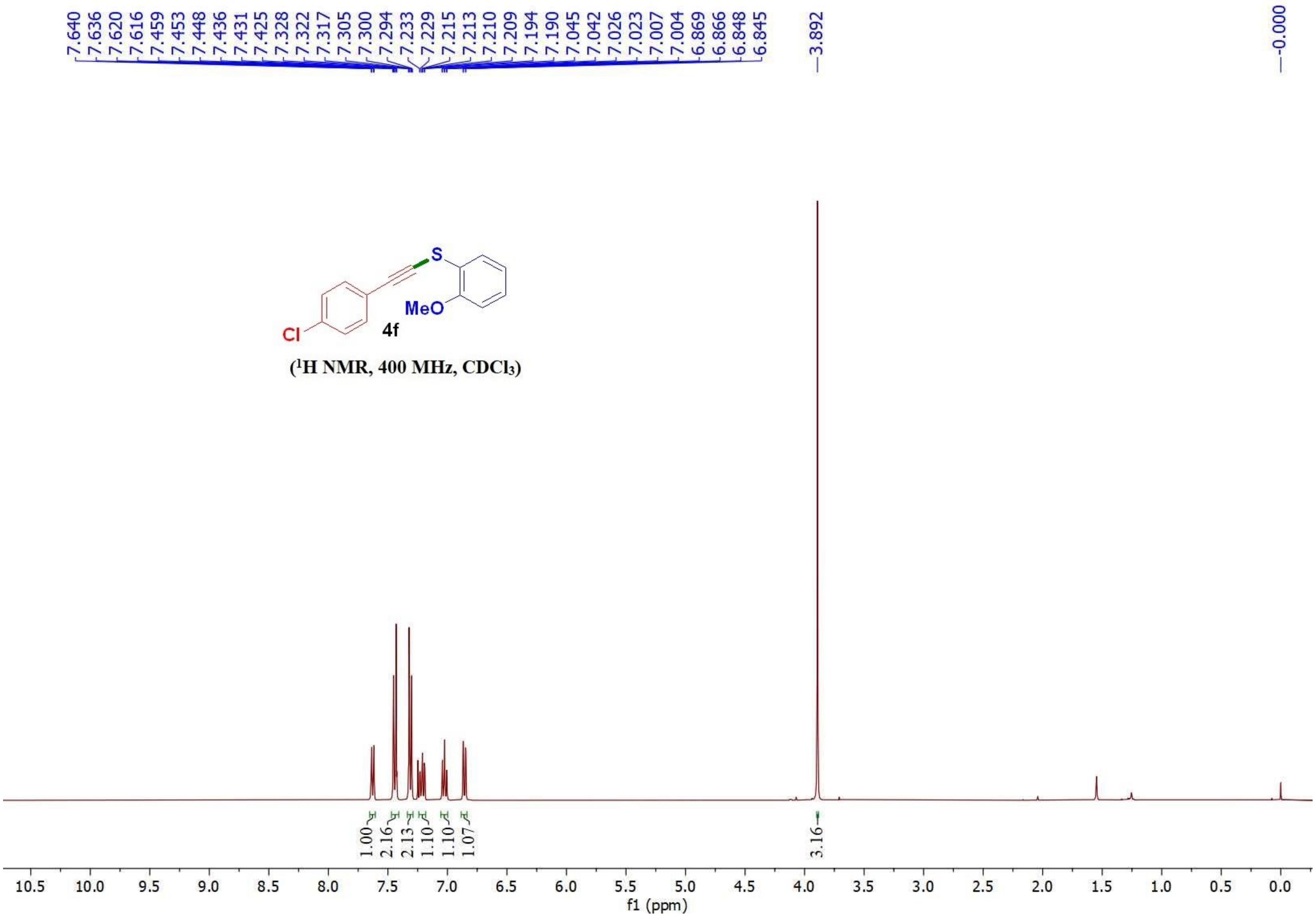

7.640
7.636
7.620
7.616
7.459
7.453
7.448
7.436
7.431
7.425
7.328
7.322
7.317
7.305
7.300
7.294
7.233
7.229
7.215
7.213
7.210
7.209
7.194
7.190
7.045
7.042
7.026
7.023
7.007
7.004
6.869
6.866
6.848
6.845
3.892
0.000
S
MeO
Cl
4f
(1H NMR, 400 MHz, CDCl3)
1.00
2.16
2.13
1.10
1.10
1.07
3.16
10.5
10.0
9.5
9.0
8.5
8.0
7.5
7.0
6.5
6.0
5.5
5.0
4.5
4.0
3.5
3.0
2.5
2.0
1.5
1.0
0.5
0.0
f1 (ppm)

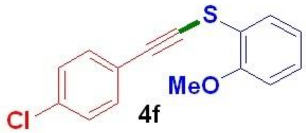
4f

(13C{H} NMR, 100 MHz, $CDCl_3$)

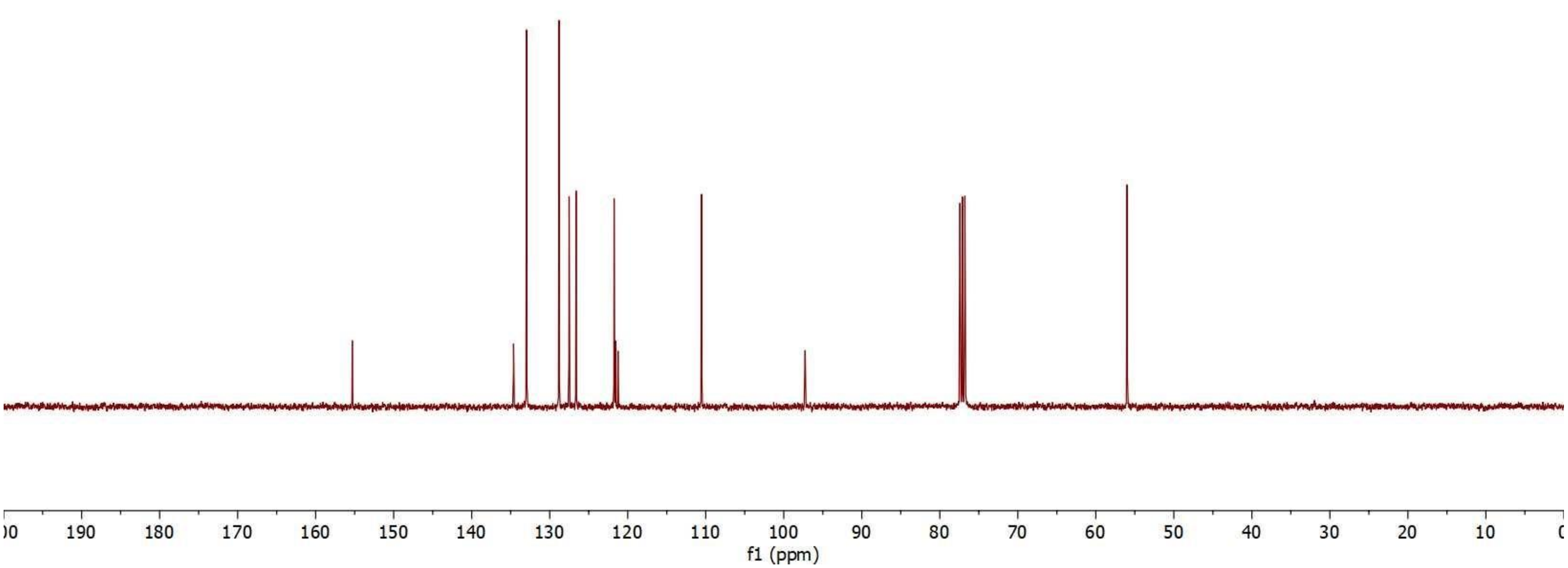

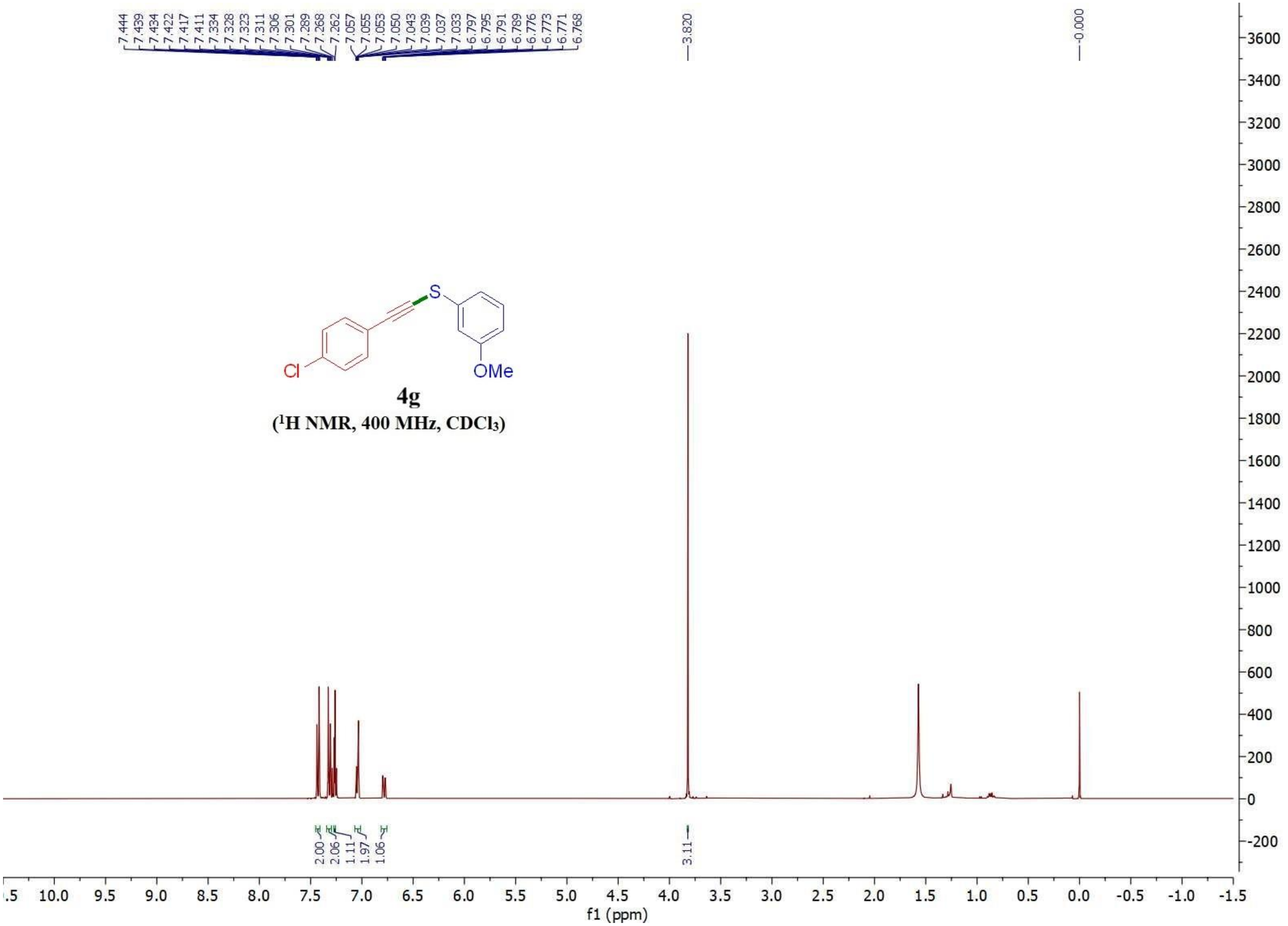

4g

(^{1}H NMR, 400 MHz, $CDCl_3$)

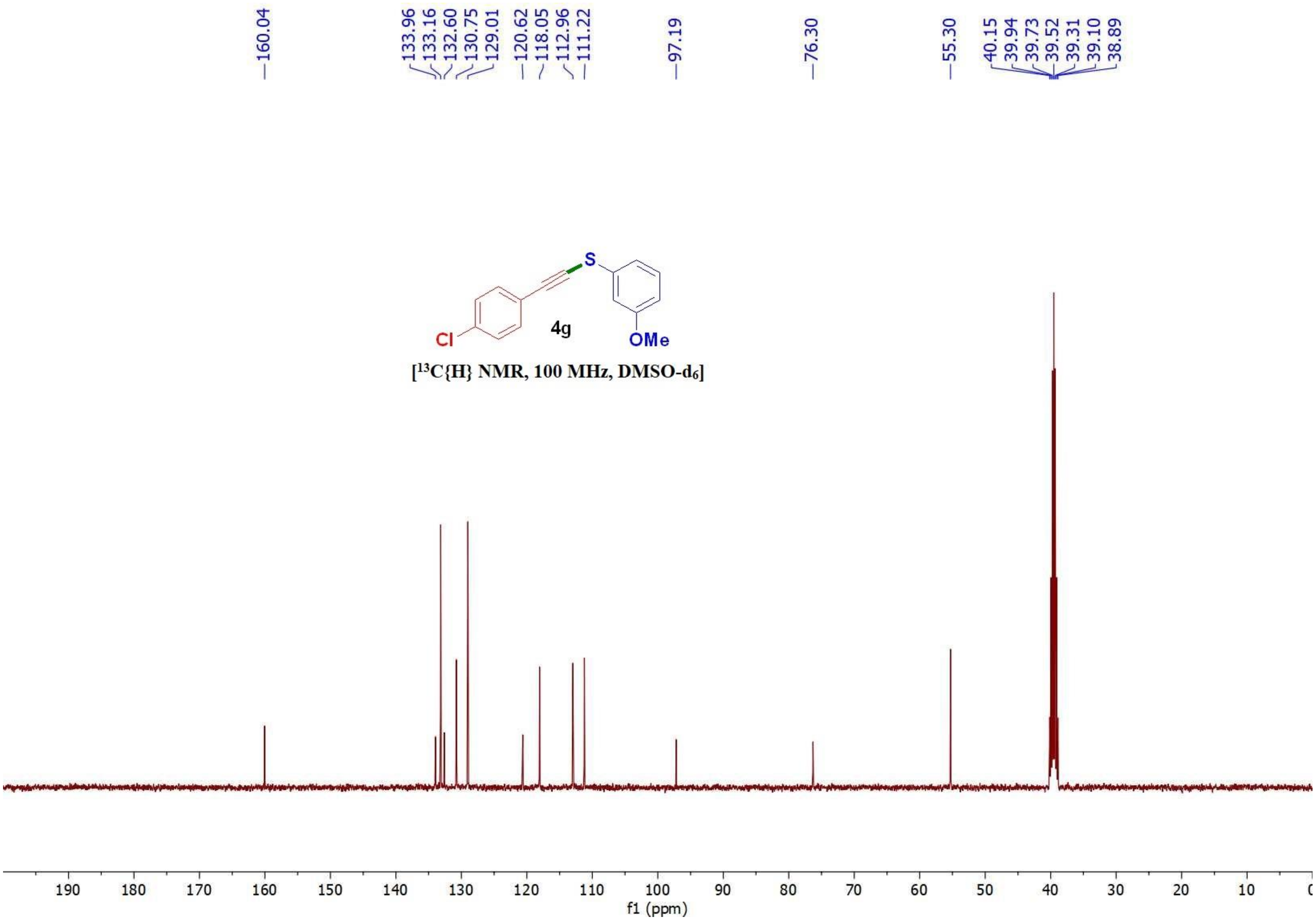
160.04
133.96
133.16
132.60
130.75
129.01
120.62
118.05
112.96
111.22
97.19
76.30
55.30
40.15
39.94
39.73
39.52
39.31
39.10
38.89
S
Cl
4g
OMe
[13C{H} NMR, 100 MHz, DMSO-d6]
190 180 170 160 150 140 130 120 110 100 90 80 70 60 50 40 30 20 10
f1 (ppm)

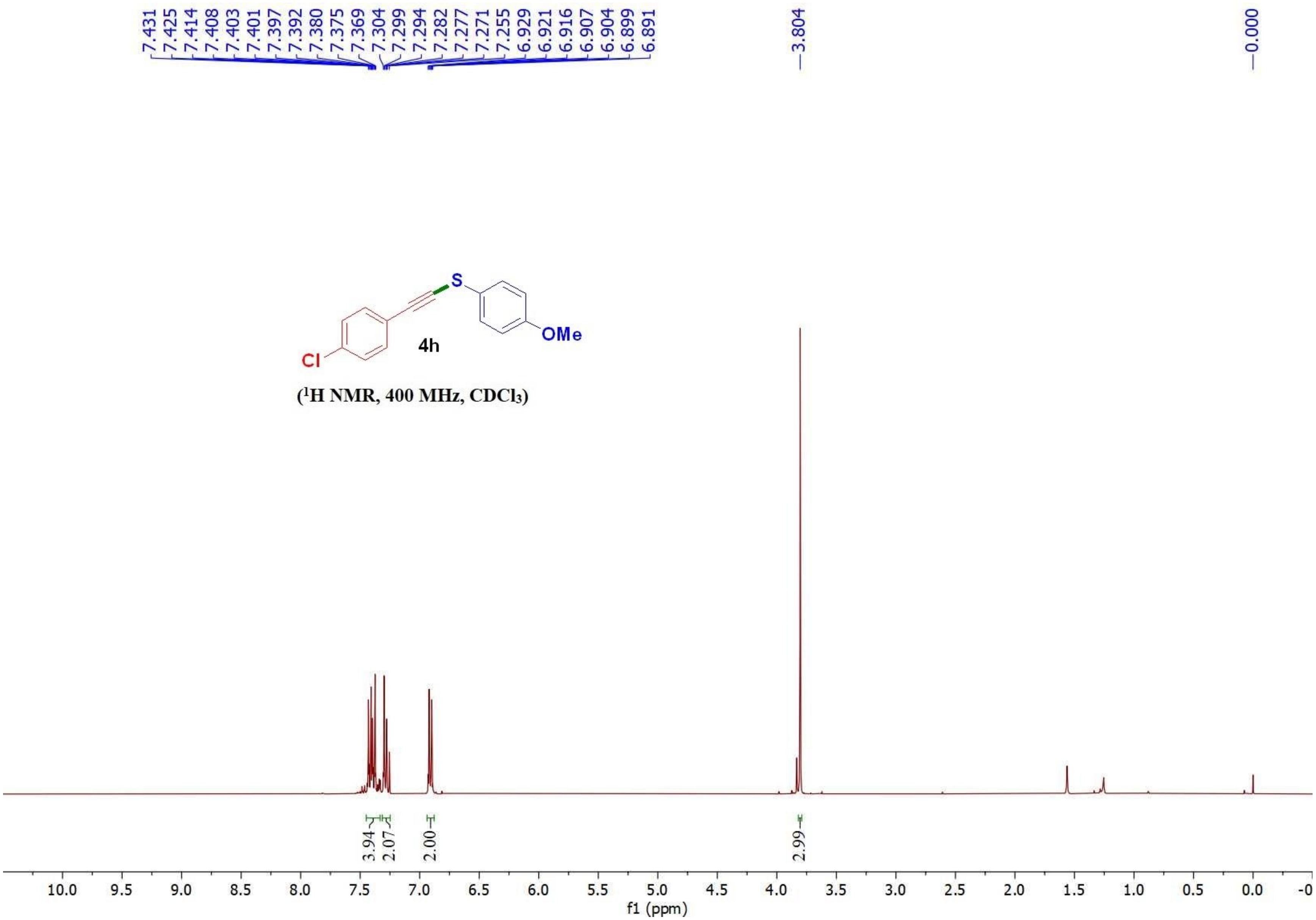

7.431
7.425
7.414
7.408
7.403
7.401
7.397
7.392
7.380
7.375
7.369
7.304
7.299
7.294
7.282
7.277
7.271
7.255
6.929
6.921
6.916
6.907
6.904
6.899
6.891
3.804
0.000
S
OMe
Cl
4h
(¹H NMR, 400 MHz, CDCl₃)
3.94
2.07
2.00
2.99
10.0
9.5
9.0
8.5
8.0
7.5
7.0
6.5
6.0
5.5
5.0
4.5
4.0
3.5
3.0
2.5
2.0
1.5
1.0
0.5
0.0
-0
f1 (ppm)

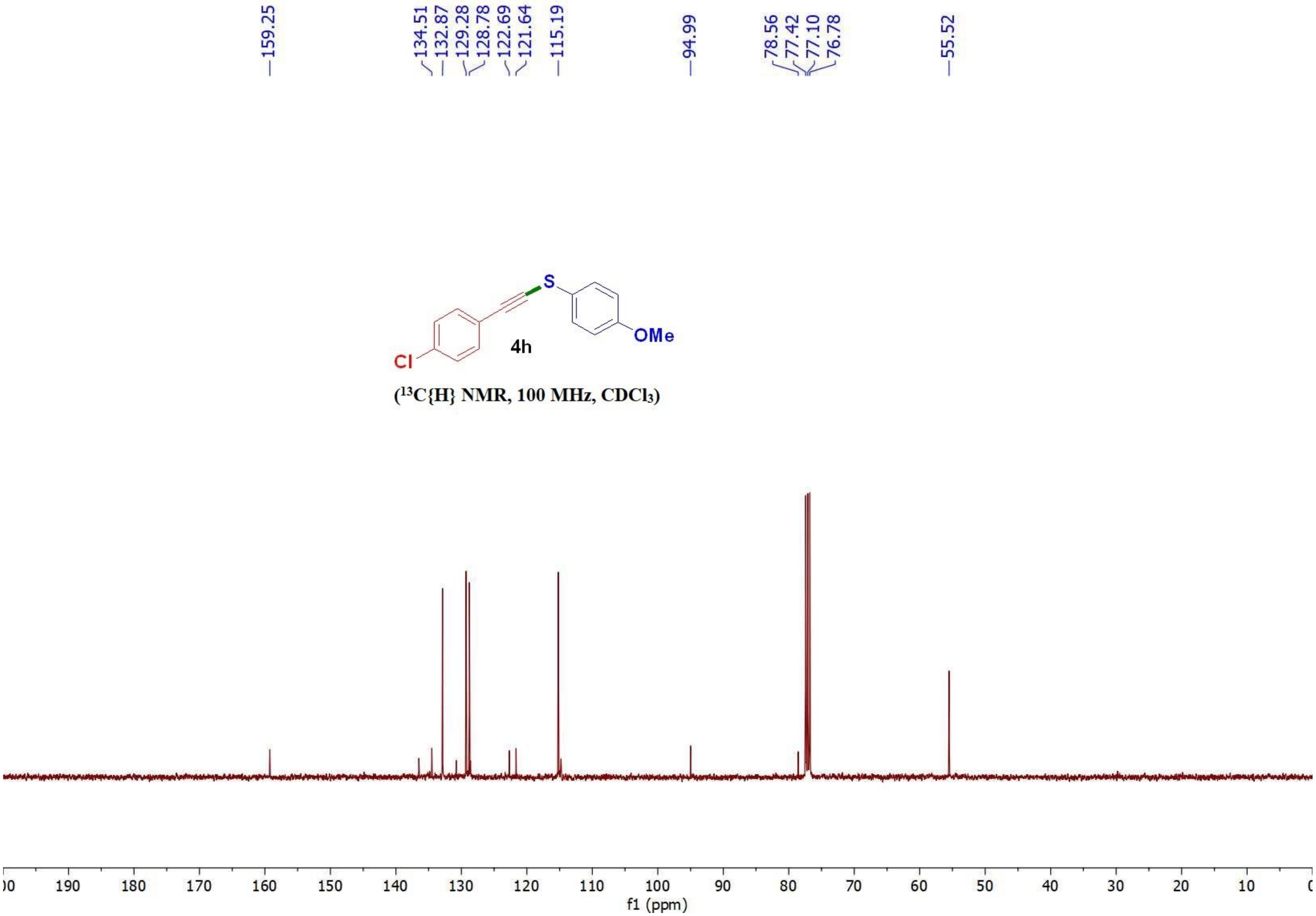

($^{13}C\{H\}$ NMR, 100 MHz, $CDCl_3$)

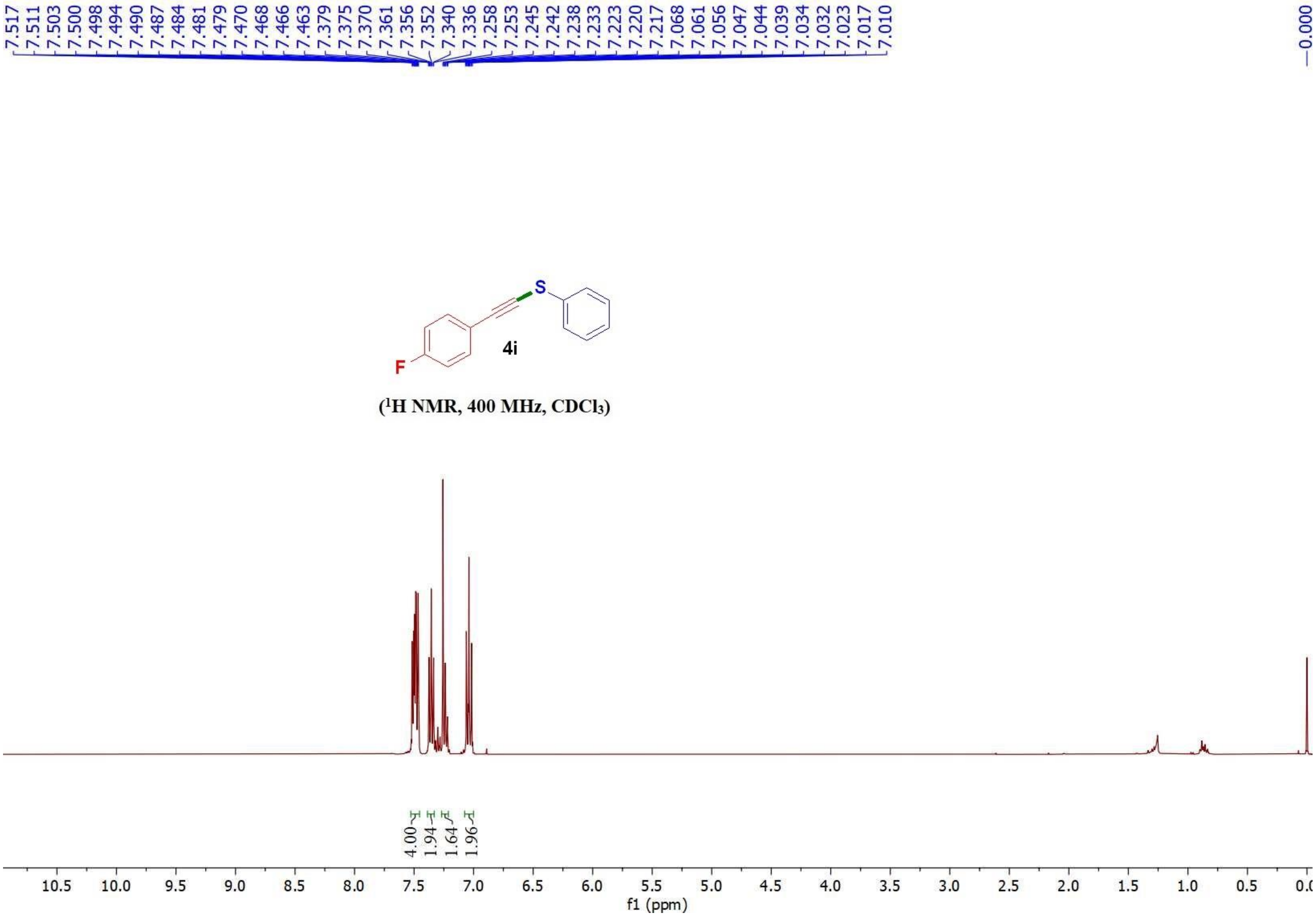
7.517 7.511 7.503 7.500 7.498 7.494 7.490 7.487 7.484 7.481 7.479 7.470 7.468 7.466 7.463 7.379 7.375 7.370 7.361 7.356 7.352 7.340 7.336 7.258 7.253 7.245 7.242 7.238 7.233 7.223 7.220 7.217 7.068 7.061 7.056 7.047 7.044 7.039 7.034 7.032 7.023 7.017 7.010
0.000
S
F
4i
(¹H NMR, 400 MHz, CDCl₃)
4.00
1.94
1.64
1.96
10.5 10.0 9.5 9.0 8.5 8.0 7.5 7.0 6.5 6.0 5.5 5.0 4.5 4.0 3.5 3.0 2.5 2.0 1.5 1.0 0.5
f1 (ppm)

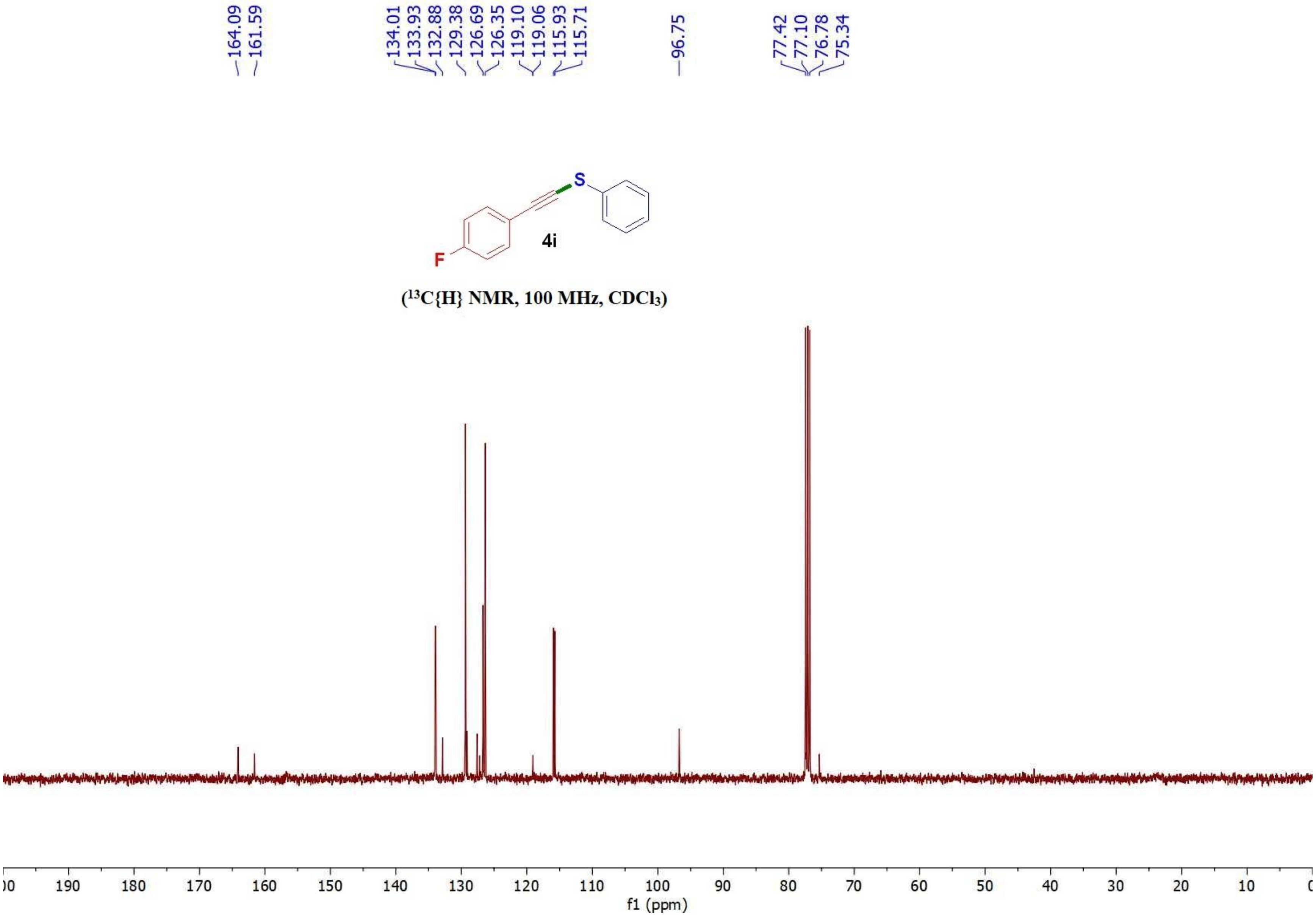
164.09
161.59
134.01
133.93
132.88
129.38
126.69
126.35
119.10
119.06
115.93
115.71
96.75
77.42
77.10
76.78
75.34
S
F
4i
(^{13}C{H} NMR, 100 MHz, CDCl$_3$)
f1 (ppm)

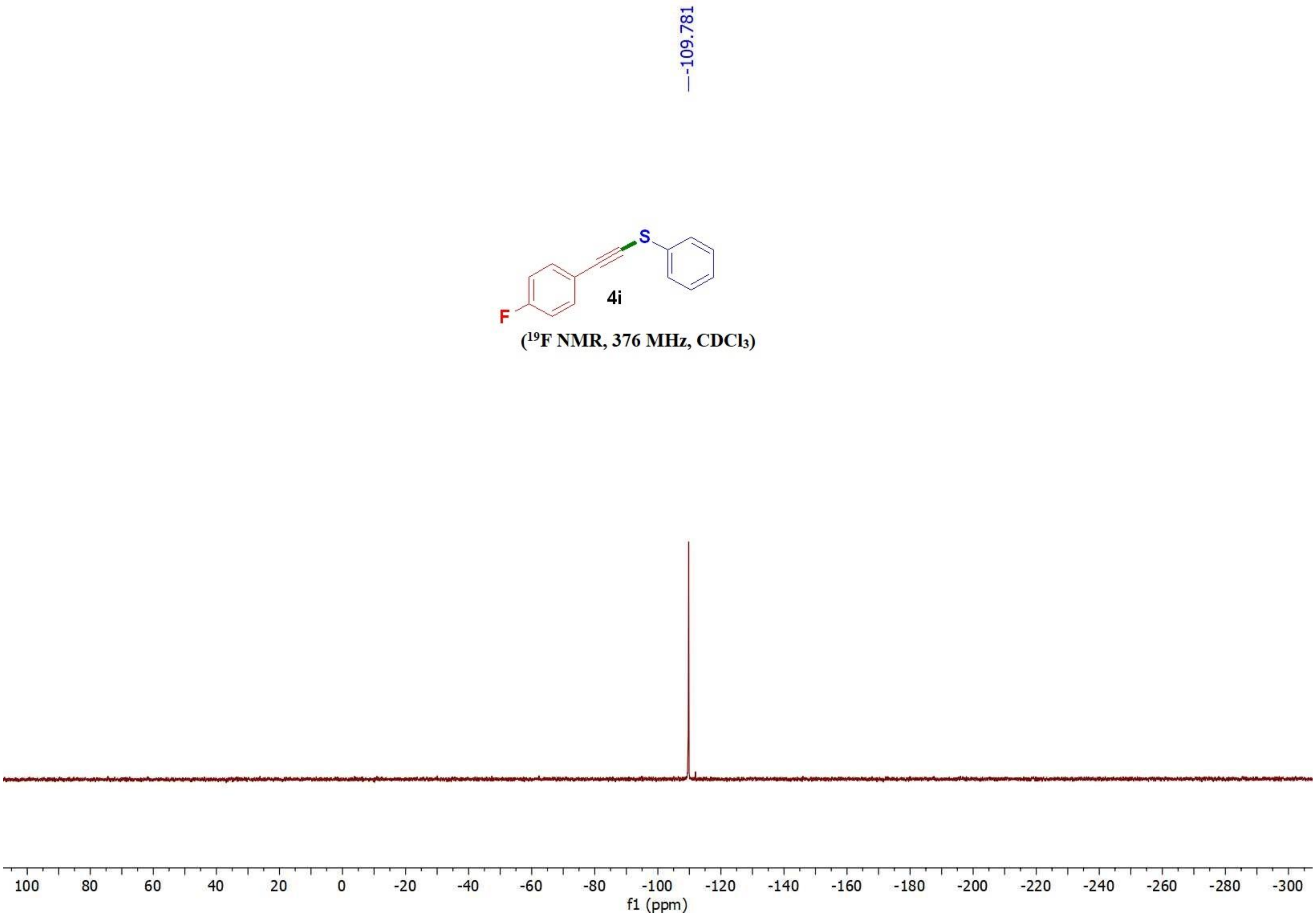

(^{19}F NMR, 376 MHz, $CDCl_3$)

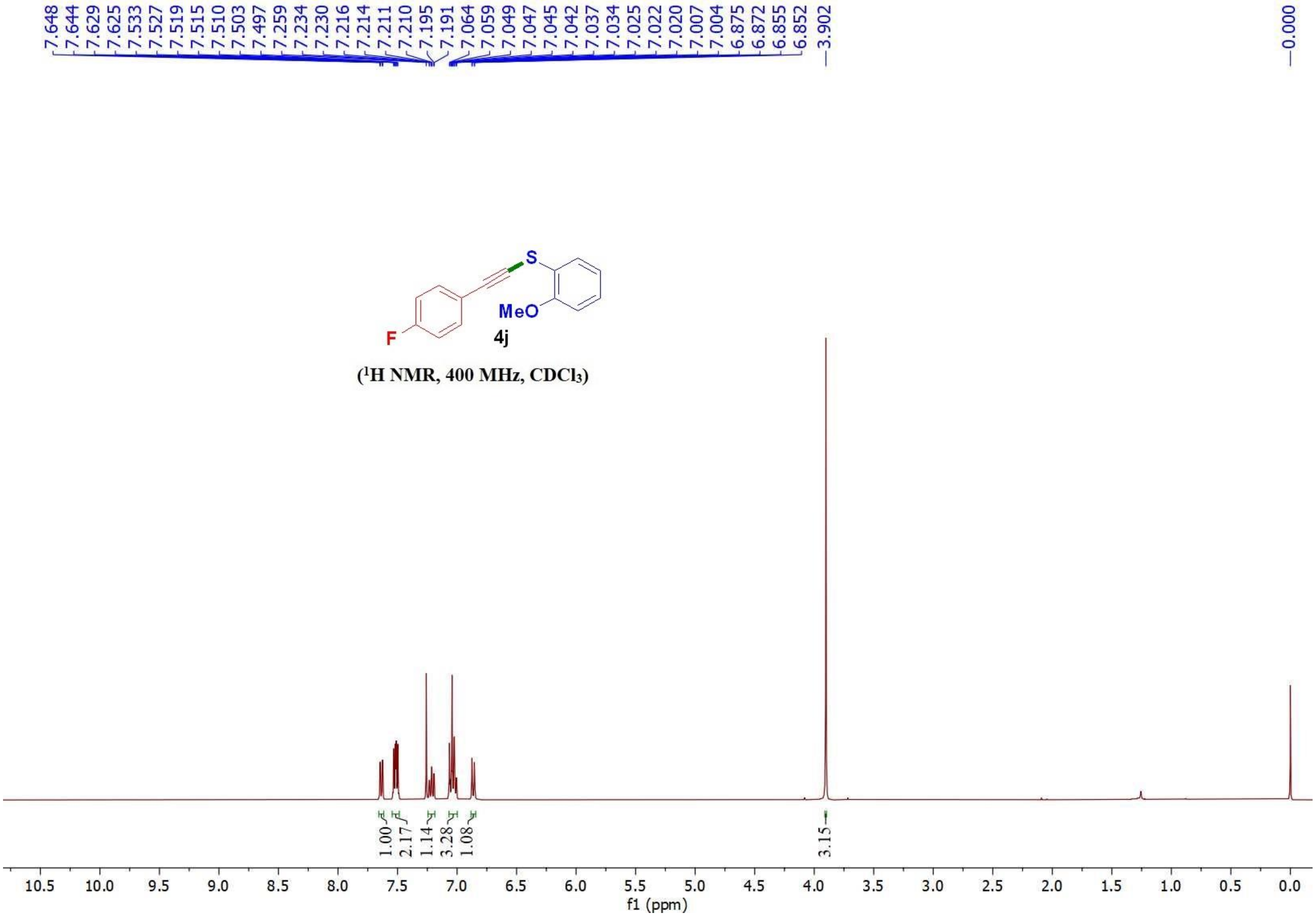
7.648
7.644
7.629
7.625
7.533
7.527
7.519
7.515
7.510
7.503
7.497
7.259
7.234
7.230
7.216
7.214
7.211
7.210
7.195
7.191
7.064
7.059
7.049
7.047
7.045
7.042
7.037
7.034
7.025
7.022
7.020
7.007
7.004
6.875
6.872
6.855
6.852
3.902
0.000
S
MeO
F
4j
(1H NMR, 400 MHz, CDCl3)
1.00
2.17
1.14
3.28
1.08
3.15
10.5 10.0 9.5 9.0 8.5 8.0 7.5 7.0 6.5 6.0 5.5 5.0 4.5 4.0 3.5 3.0 2.5 2.0 1.5 1.0 0.5 0.0
f1 (ppm)

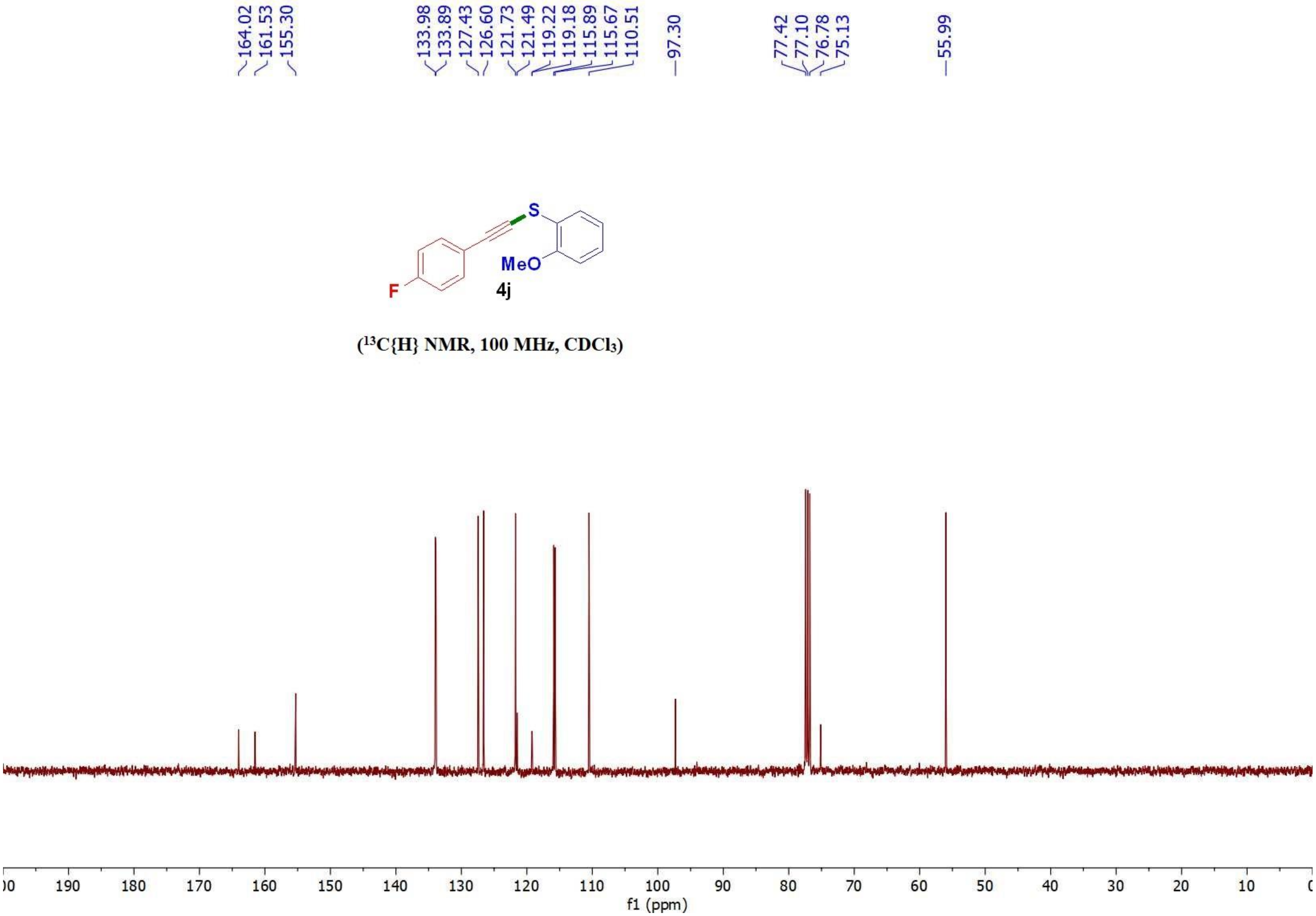

($^{13}C\{H\}$ NMR, 100 MHz, $CDCl_3$)

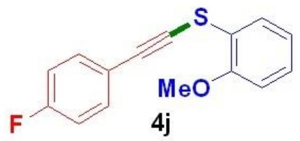

4j

(^{19}F NMR, 376 MHz, $CDCl_3$)

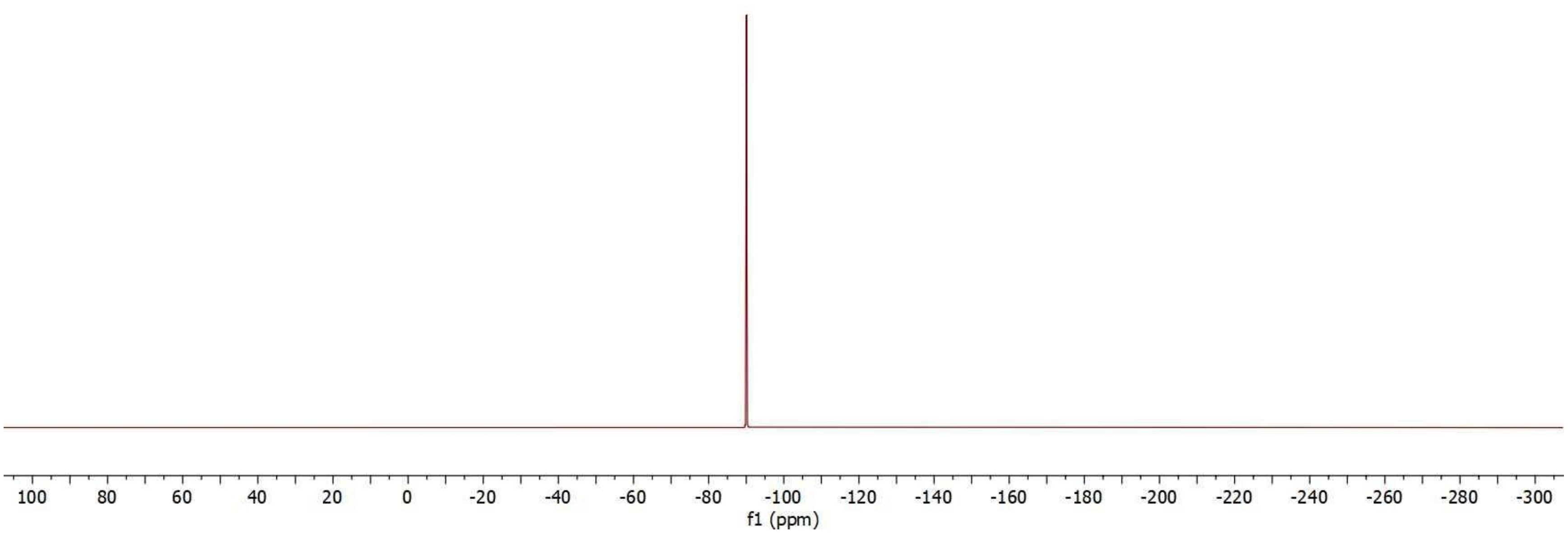

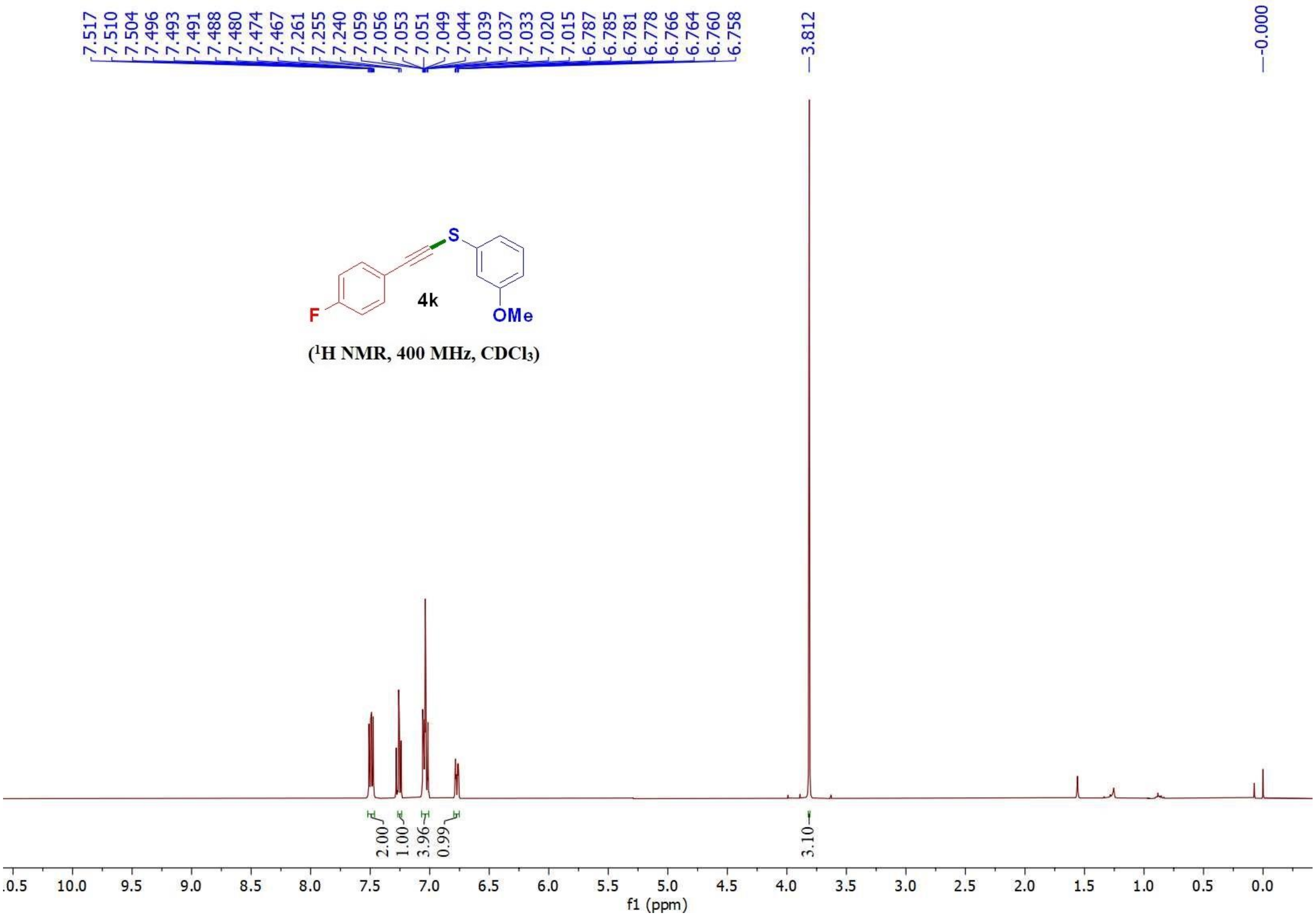
7.517
7.510
7.504
7.496
7.493
7.491
7.488
7.480
7.474
7.467
7.261
7.255
7.240
7.059
7.056
7.053
7.051
7.049
7.044
7.039
7.037
7.033
7.020
7.015
6.787
6.785
6.781
6.778
6.766
6.764
6.760
6.758
3.812
-0.000
S
F
4k
OMe
(1H NMR, 400 MHz, CDCl3)
2.00
1.00
3.96
0.99
3.10
f1 (ppm)

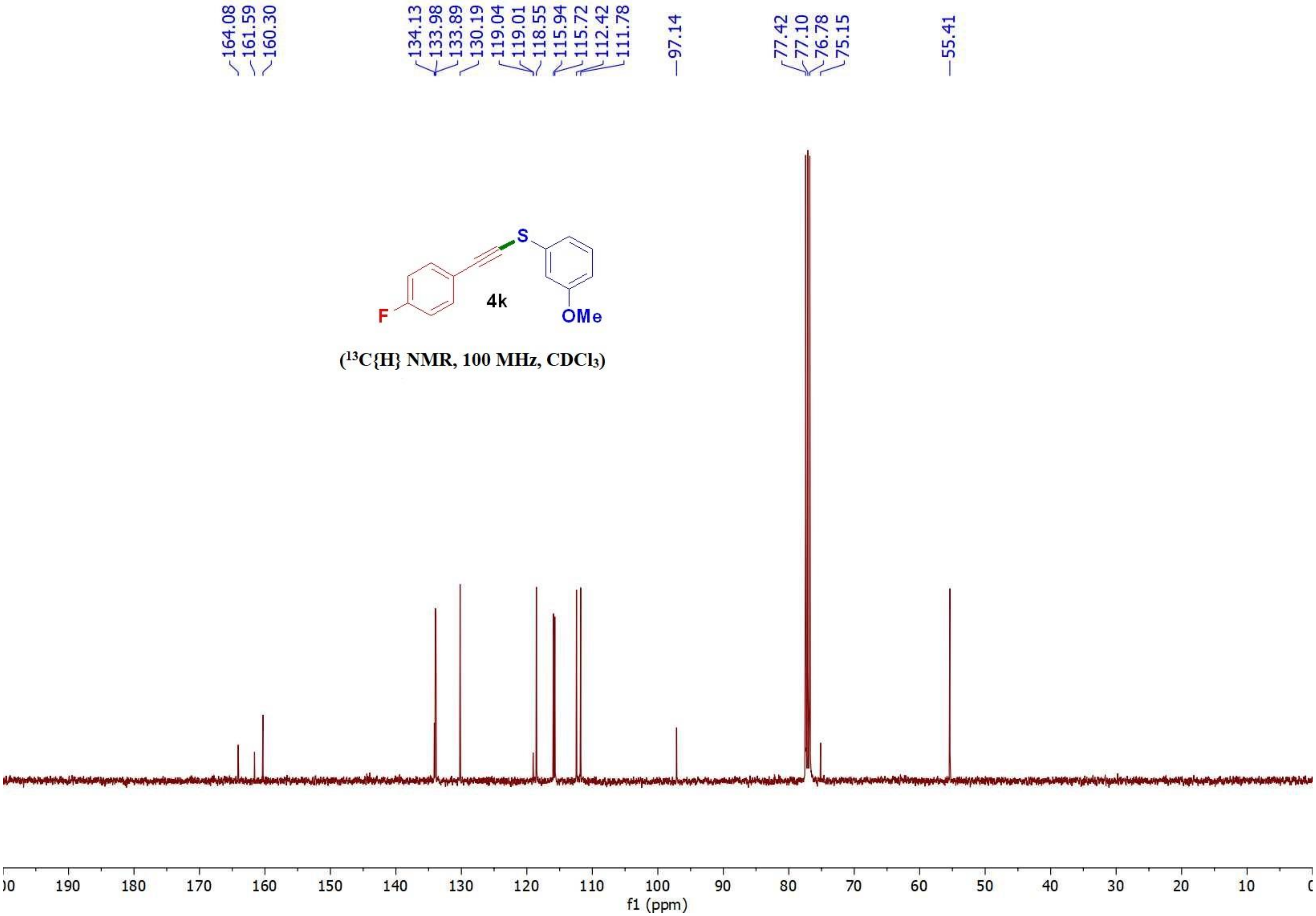
164.08
161.59
160.30
134.13
133.98
133.89
130.19
119.04
119.01
118.55
115.94
115.72
112.42
111.78
97.14
77.42
77.10
76.78
75.15
55.41
S
F
4k
OMe
(^{13}C{H} NMR, 100 MHz, CDCl$_3$)
190
180
170
160
150
140
130
120
110
100
90
80
70
60
50
40
30
20
10
f1 (ppm)

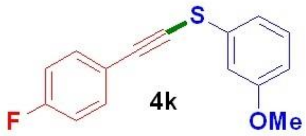

4k

(${}^{19}F$ NMR, 376 MHz, $CDCl_3$)

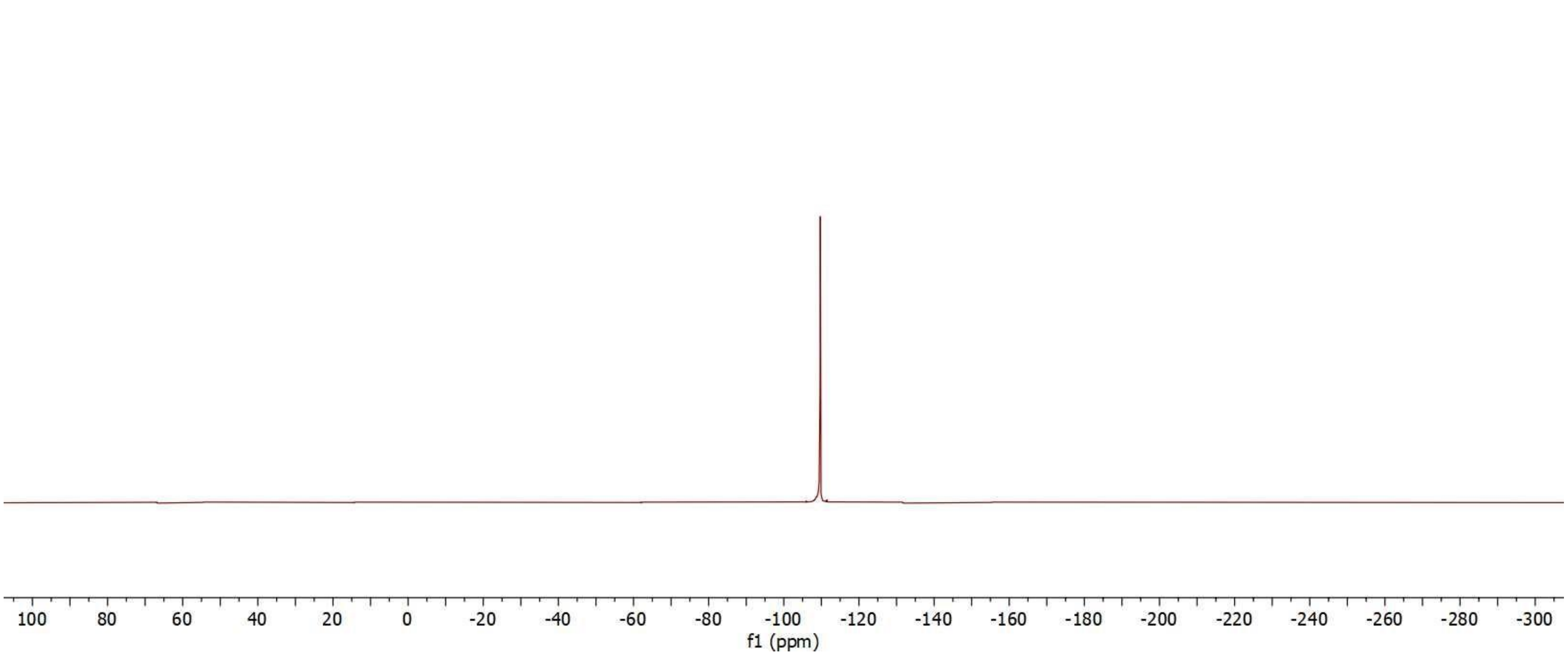

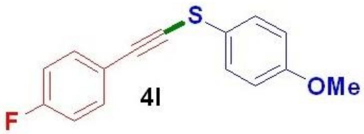

4l

(¹H NMR, 400 MHz, CDCl₃)

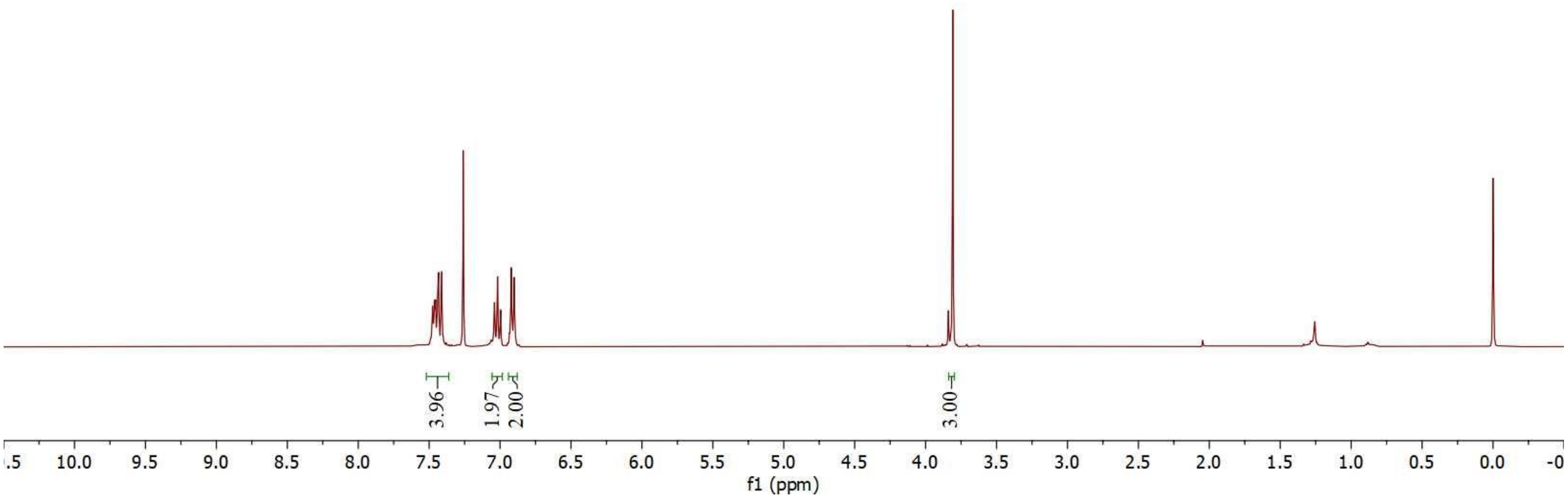

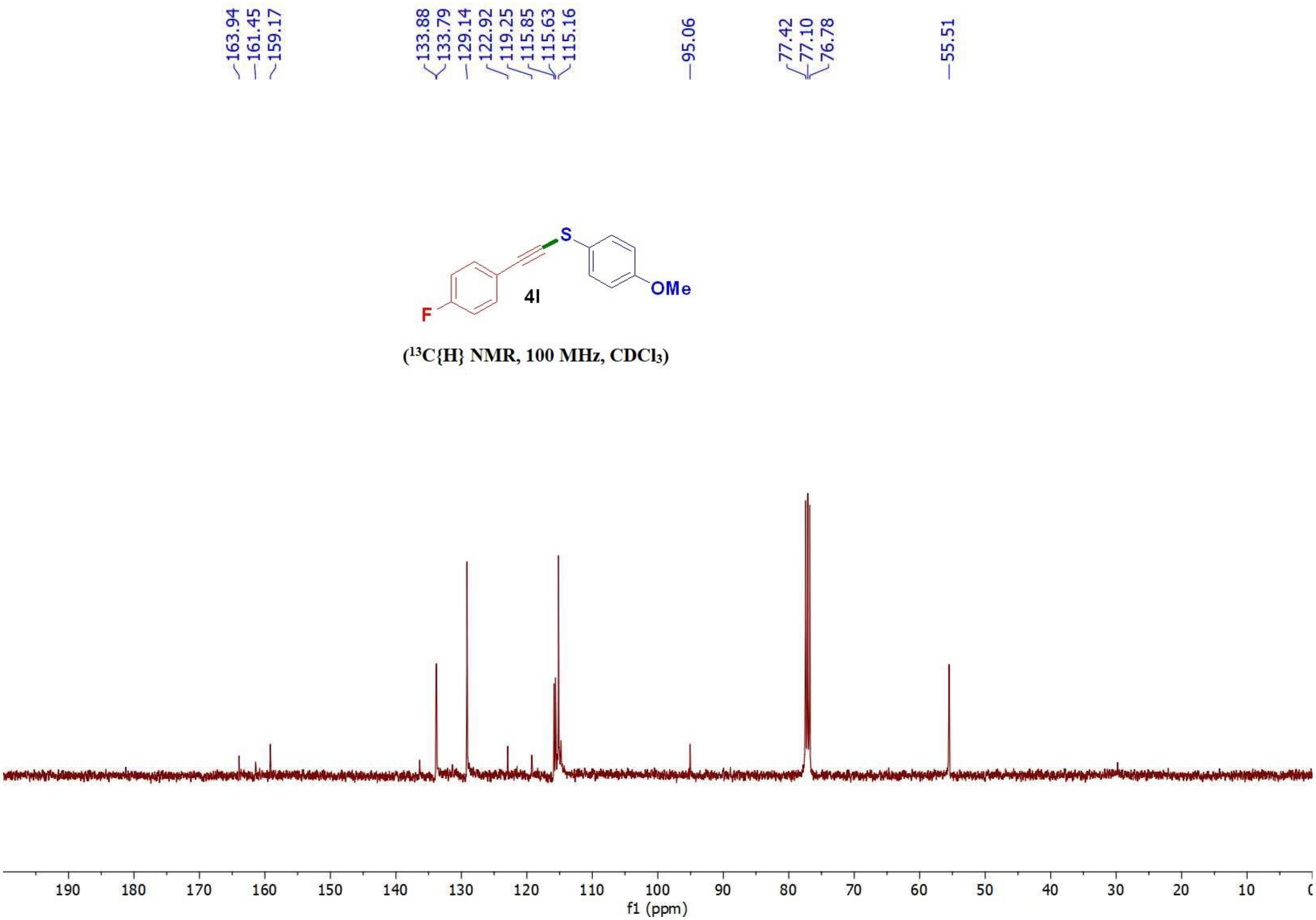

($^{13}C\{H\}$ NMR, 100 MHz, $CDCl_3$)

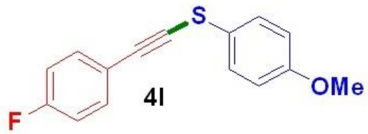

4l

(^{19}F NMR, 376 MHz, $CDCl_3$)

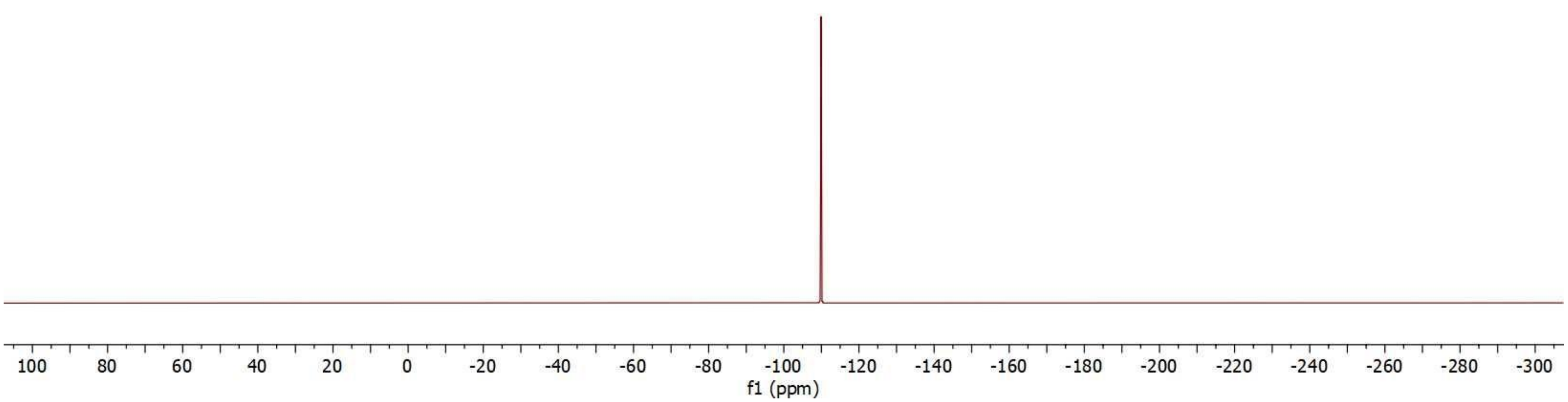

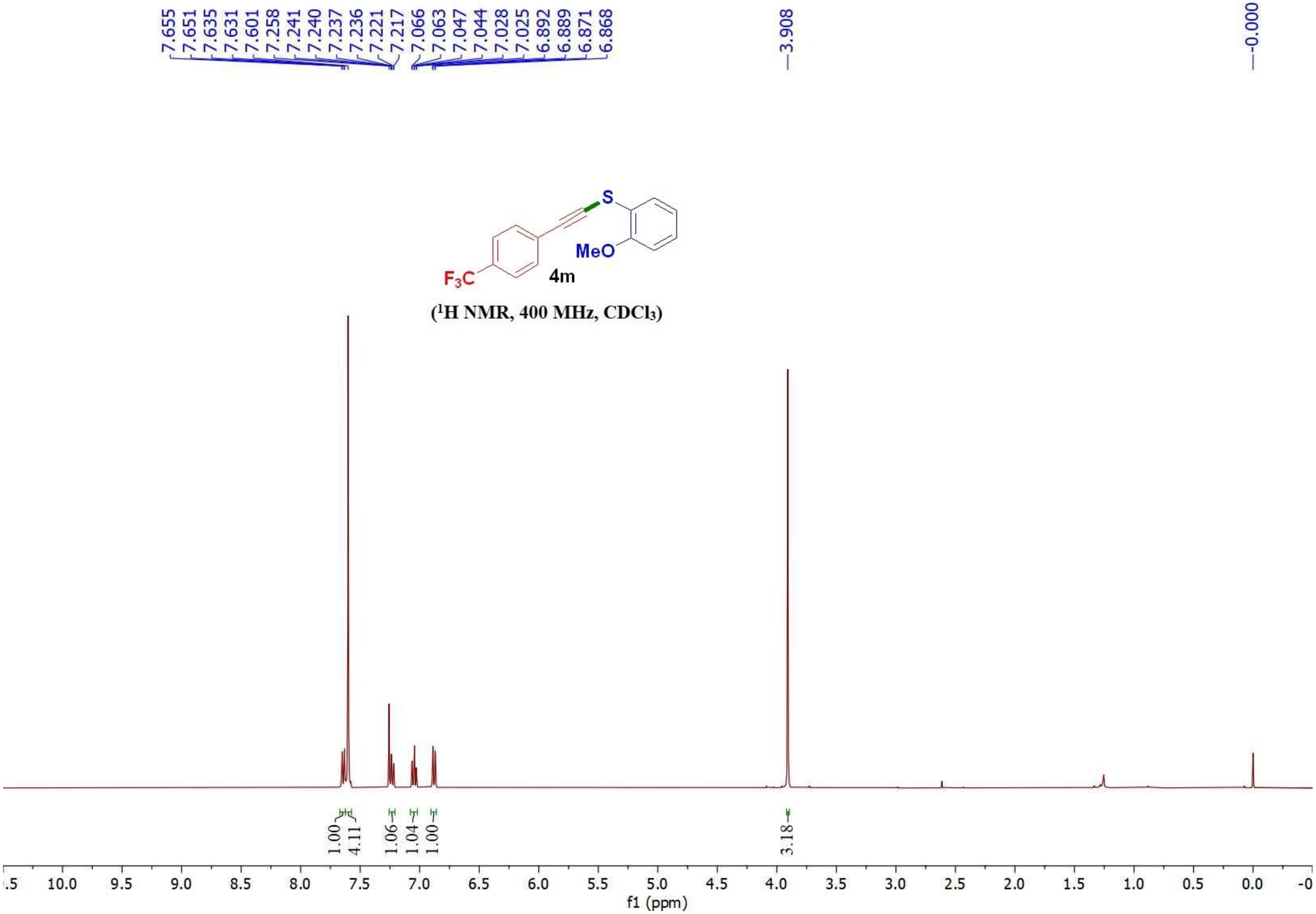
7.655
7.651
7.635
7.631
7.601
7.258
7.241
7.240
7.237
7.236
7.221
7.217
7.066
7.063
7.047
7.044
7.028
7.025
6.892
6.889
6.871
6.868
3.908
0.000
S
MeO
F_3C
4m
(^1H NMR, 400 MHz, $CDCl_3$)
1.00
4.11
1.06
1.04
1.00
3.18
f1 (ppm)

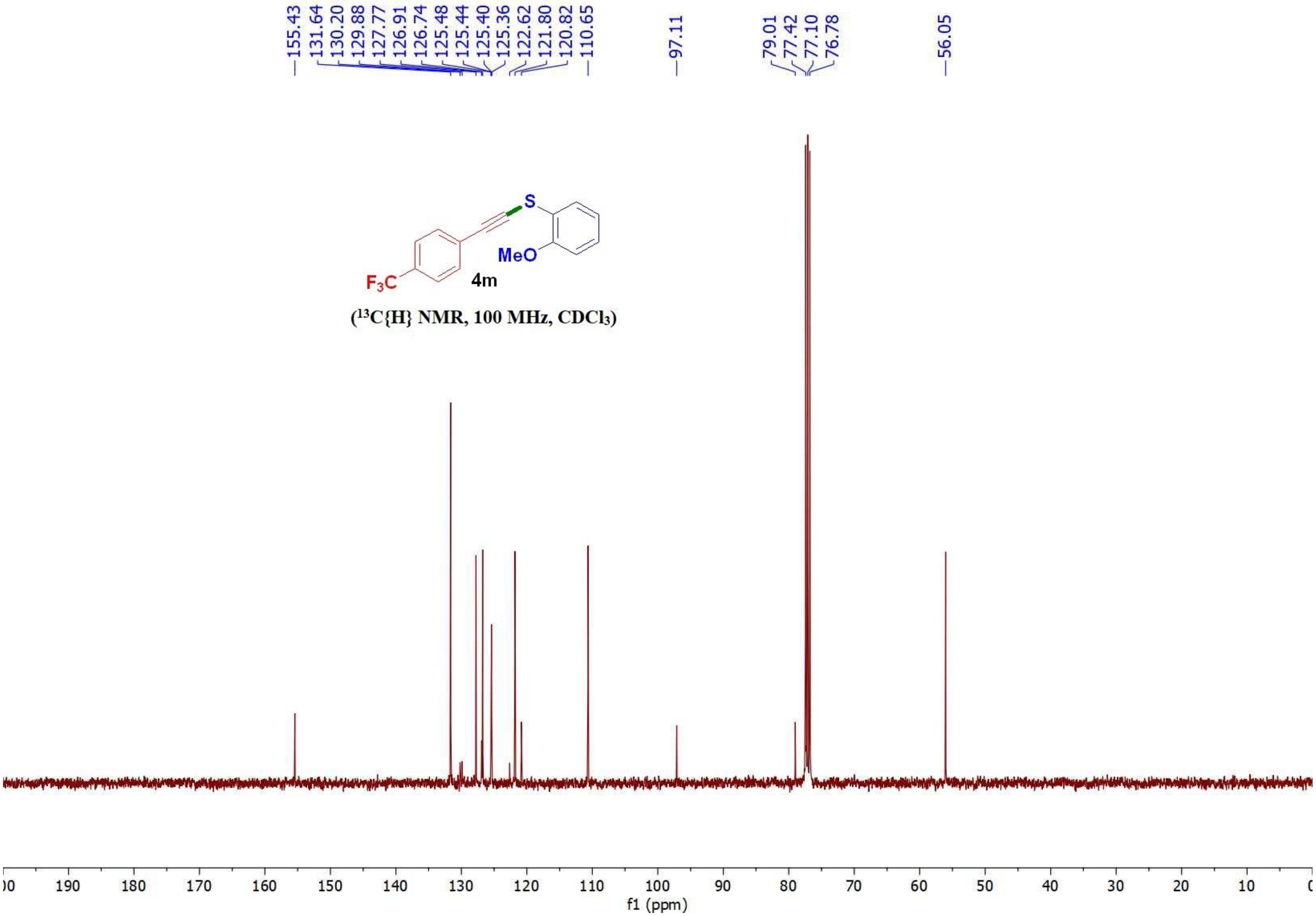
155.43
131.64
130.20
129.88
127.77
126.91
126.74
125.48
125.44
125.40
125.36
122.62
121.80
120.82
110.65
97.11
79.01
77.42
77.10
76.78
56.05
S
MeO
F3C
4m
(13C{H} NMR, 100 MHz, CDCl3)
190
180
170
160
150
140
130
120
110
100
90
80
70
60
50
40
30
20
10
f1 (ppm)

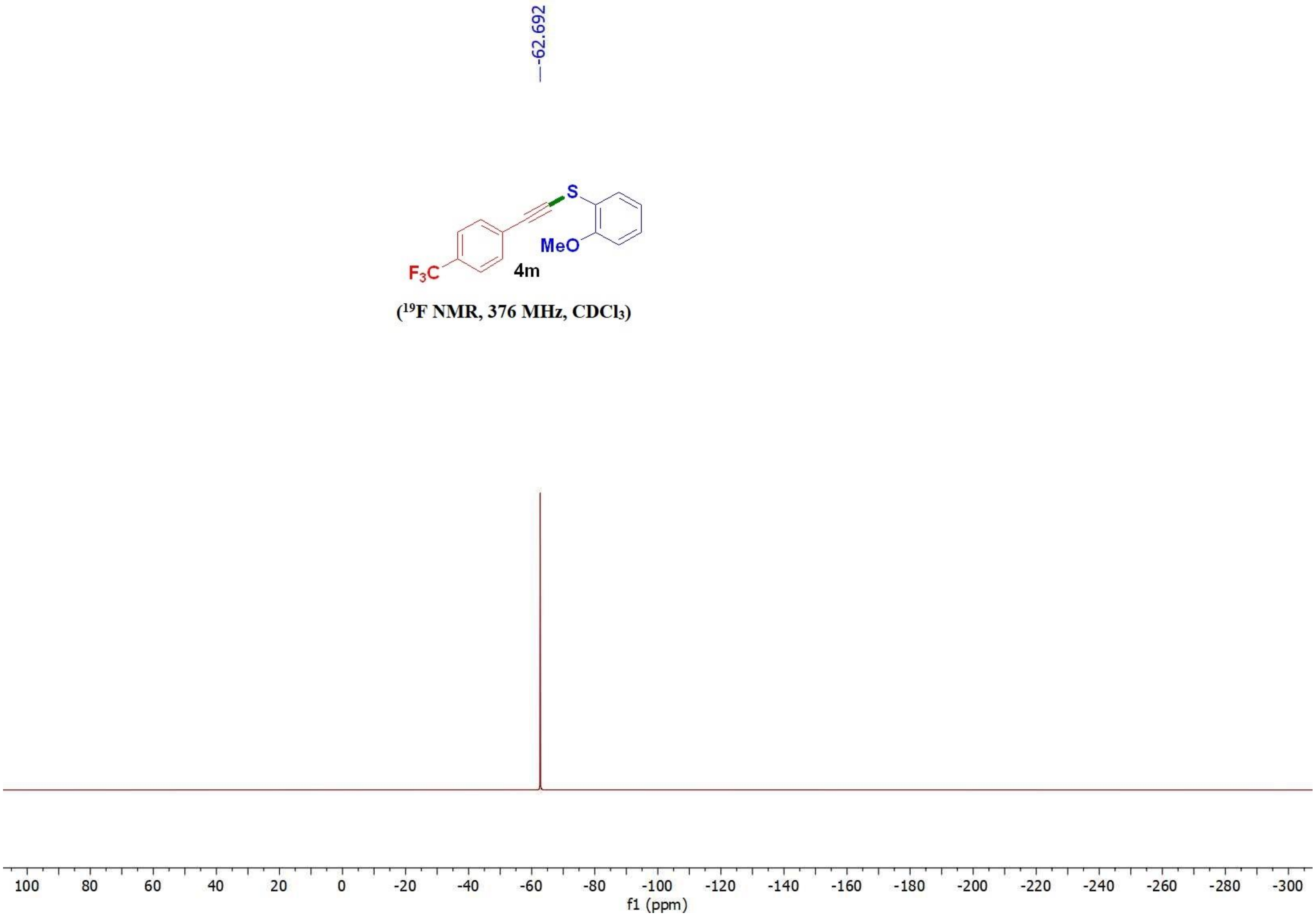
-62.692
S
MeO
F3C
4m
(19F NMR, 376 MHz, CDCl3)
100 80 60 40 20 0 -20 -40 -60 -80 -100 -120 -140 -160 -180 -200 -220 -240 -260 -280 -300
f1 (ppm)

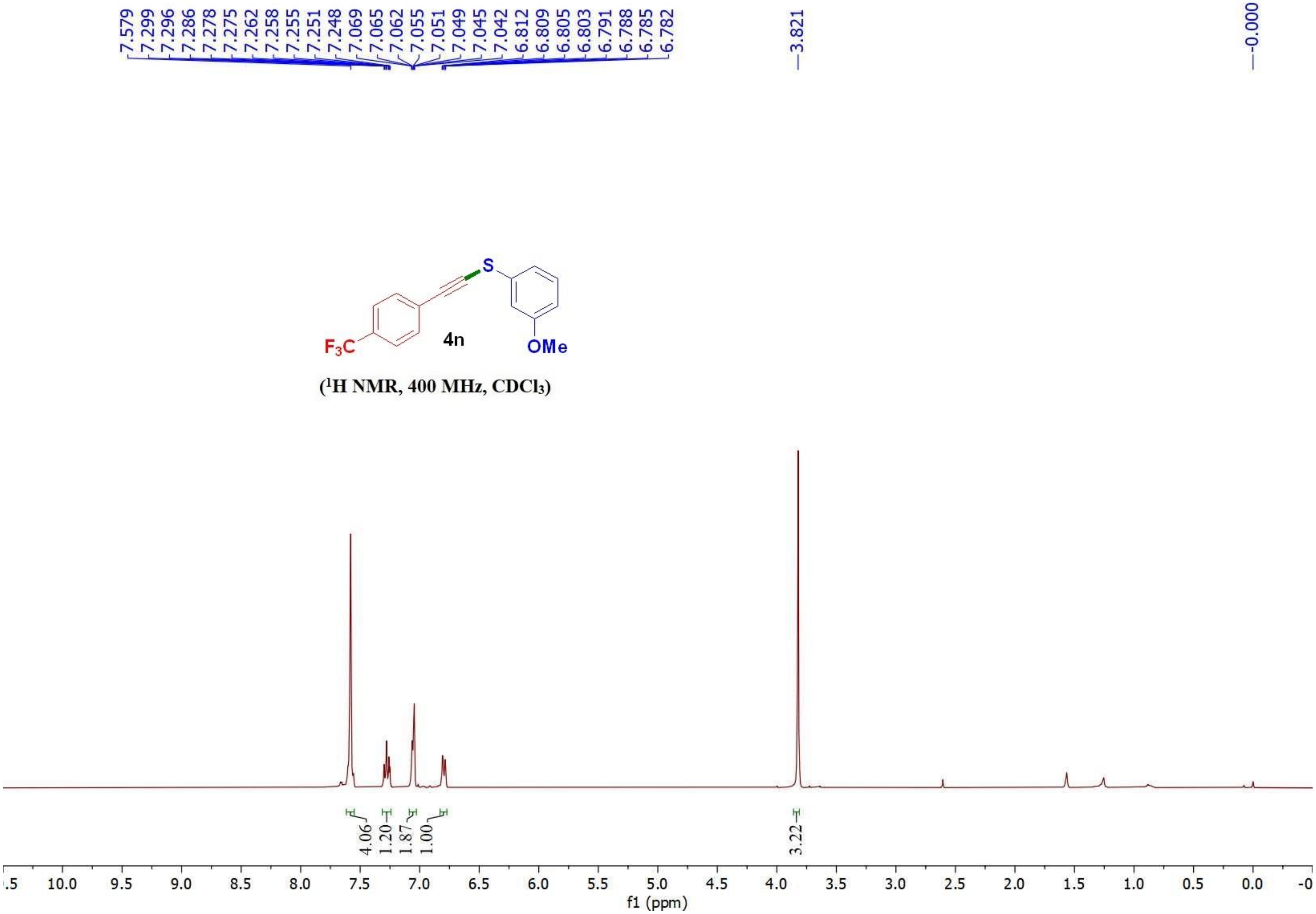

7.579
7.299
7.296
7.286
7.278
7.275
7.262
7.258
7.255
7.251
7.248
7.069
7.065
7.062
7.055
7.051
7.049
7.045
7.042
6.812
6.809
6.805
6.803
6.791
6.788
6.785
6.782
3.821
-0.000
F3C
S
4n
OMe
(1H NMR, 400 MHz, CDCl3)
4.06
1.20
1.87
1.00
3.22
f1 (ppm)

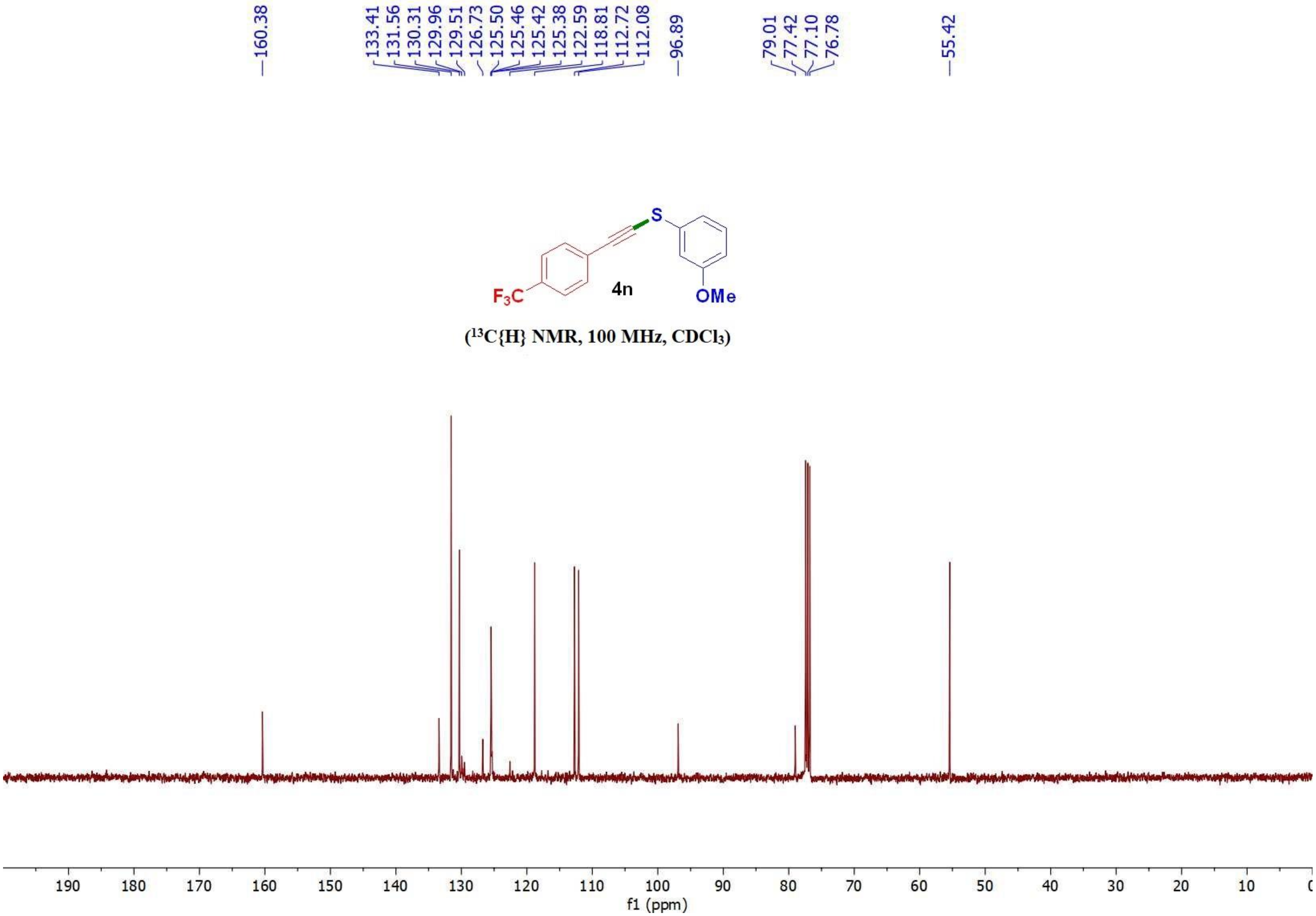

(^{13}C{H} NMR, 100 MHz, $CDCl_3$)

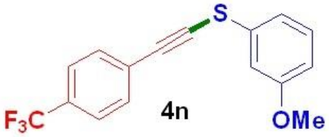

4n

(19F NMR, 376 MHz, $CDCl_3$)

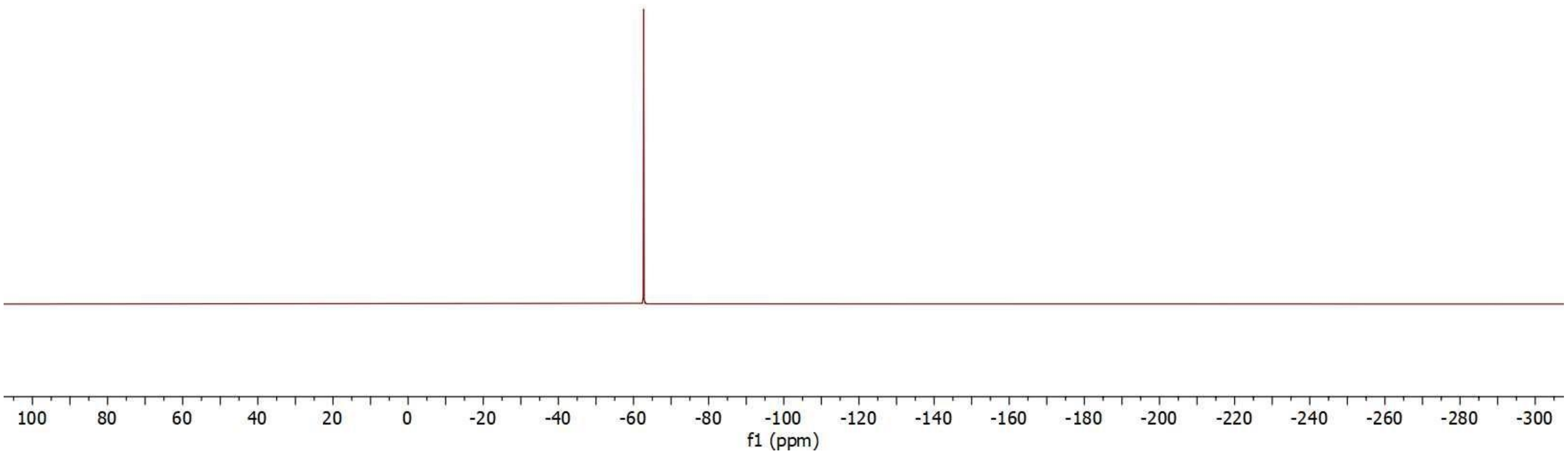

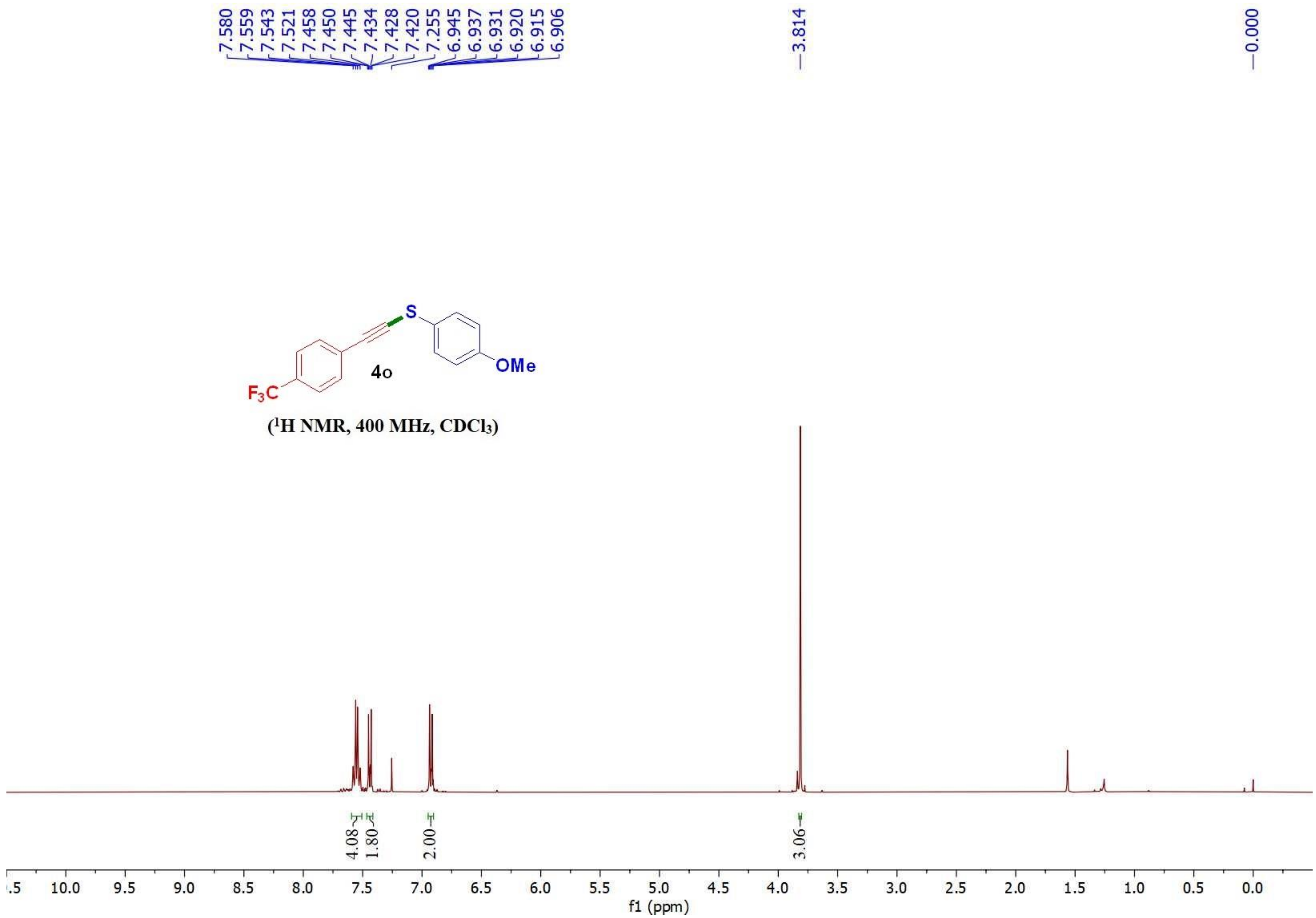
7.580
7.559
7.543
7.521
7.458
7.450
7.445
7.434
7.428
7.420
7.255
6.945
6.937
6.931
6.920
6.915
6.906
3.814
0.000
S
OMe
4o
F_3C
(^{1}H NMR, 400 MHz, $CDCl_3$)
4.08
1.80
2.00
3.06
.5
10.0
9.5
9.0
8.5
8.0
7.5
7.0
6.5
6.0
5.5
5.0
4.5
4.0
3.5
3.0
2.5
2.0
1.5
1.0
0.5
0.0
f1 (ppm)

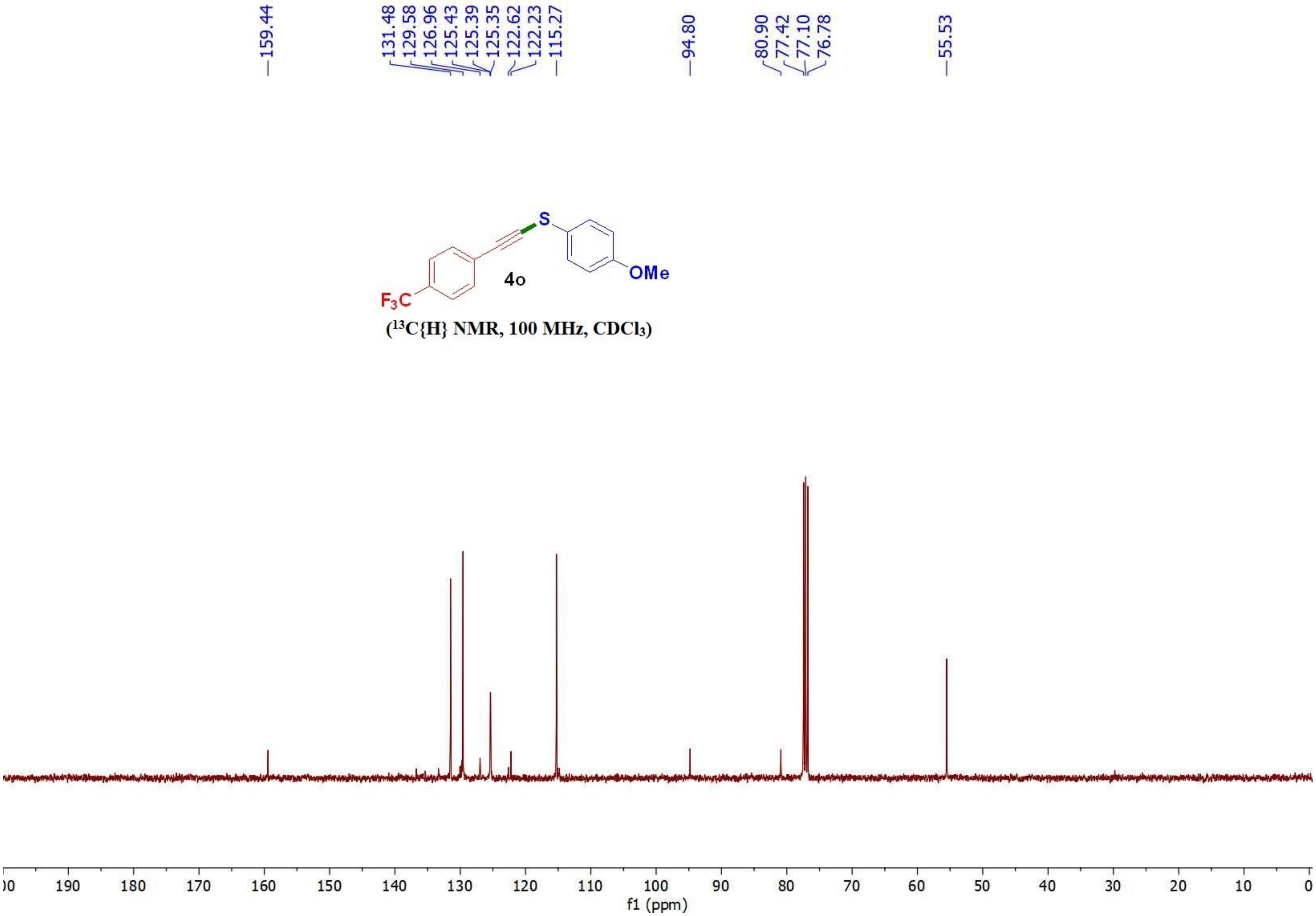
159.44
131.48
129.58
126.96
125.43
125.39
125.35
122.62
122.23
115.27
94.80
80.90
77.42
77.10
76.78
55.53
S
OMe
F_3C
4o
($^{13}C\{H\}$ NMR, 100 MHz, $CDCl_3$)
190 180 170 160 150 140 130 120 110 100 90 80 70 60 50 40 30 20 10 0
f1 (ppm)

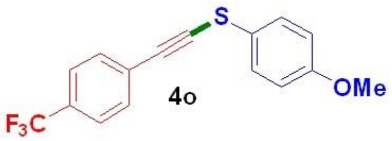

(${}^{19}F$ NMR, 376 MHz, $CDCl_3$)

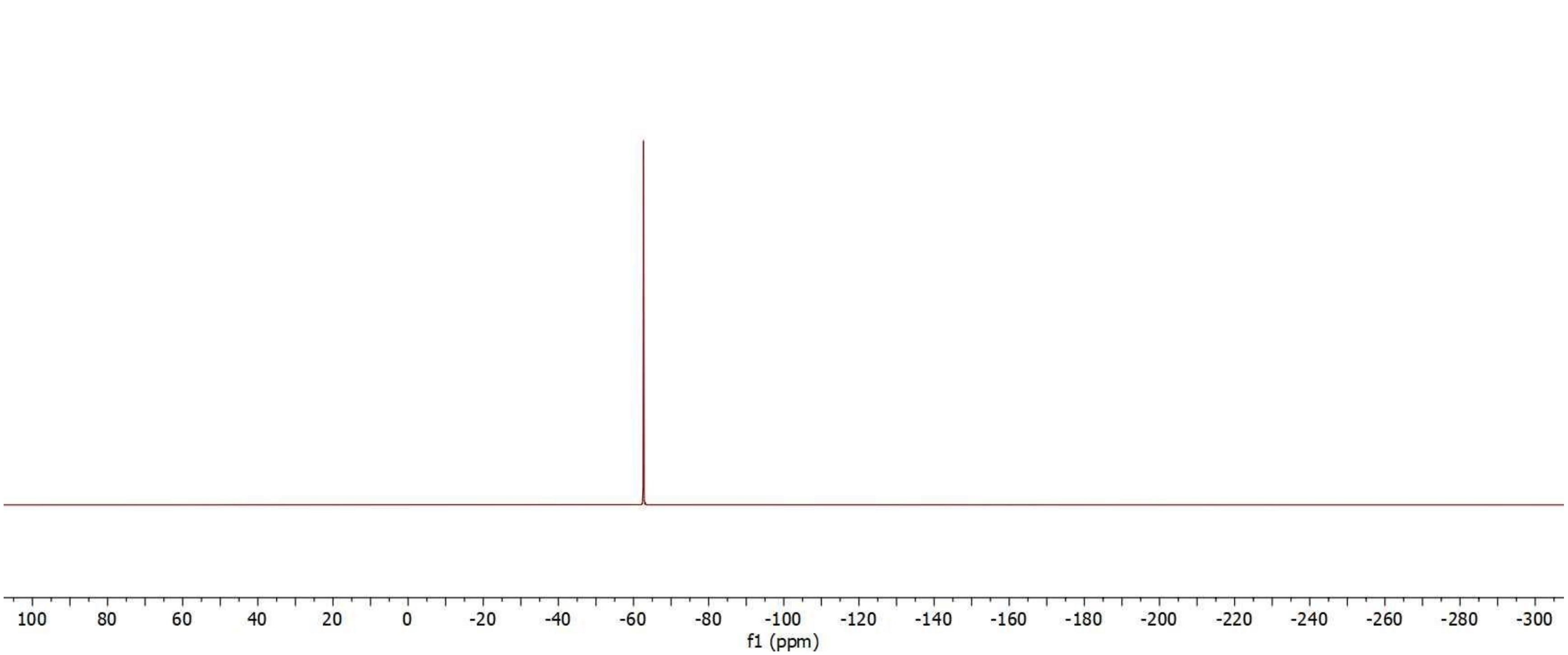

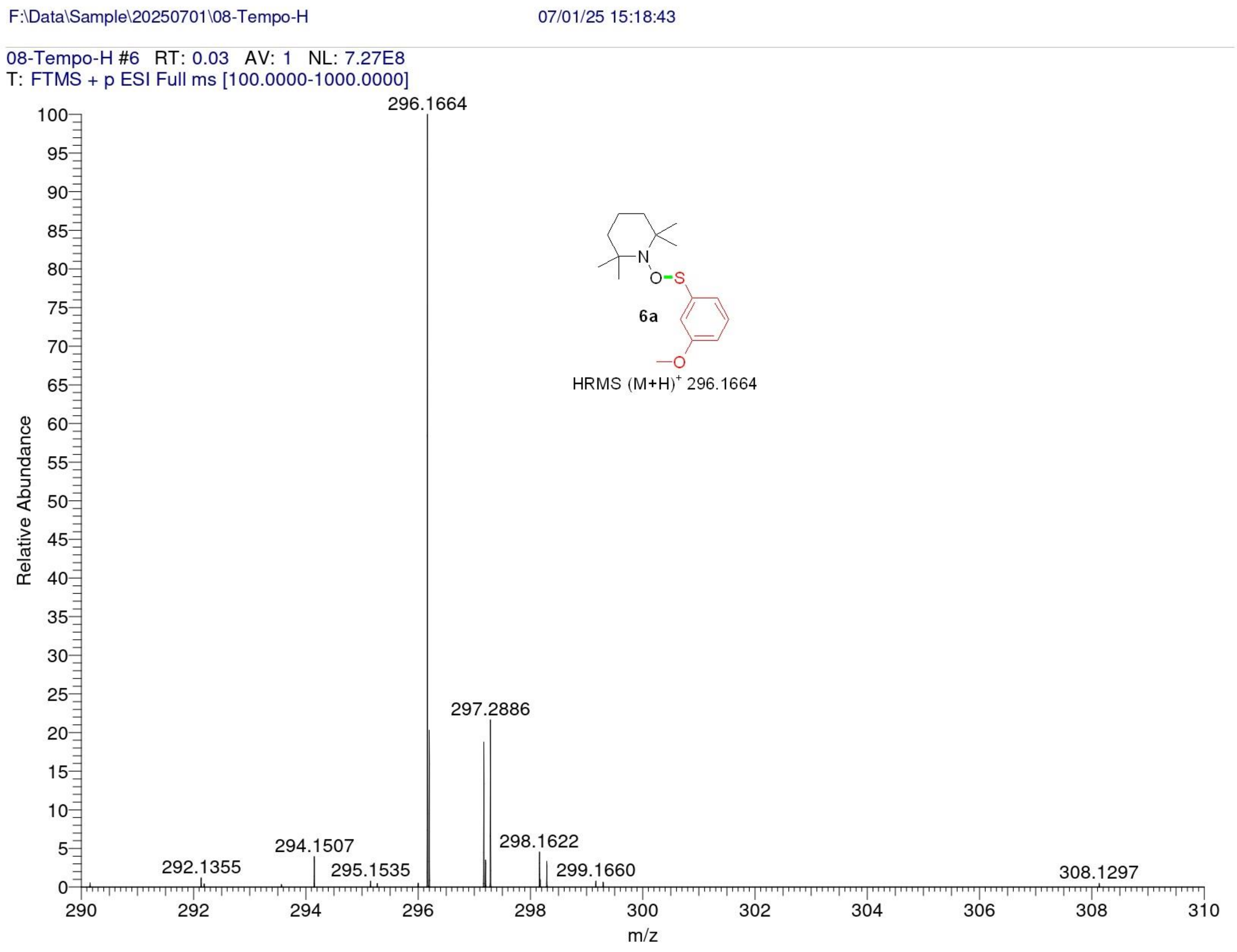

Figure S5: HRMS spectrum of the 3-methoxybenzenethiyl radical/TEMPO adduct **6a**

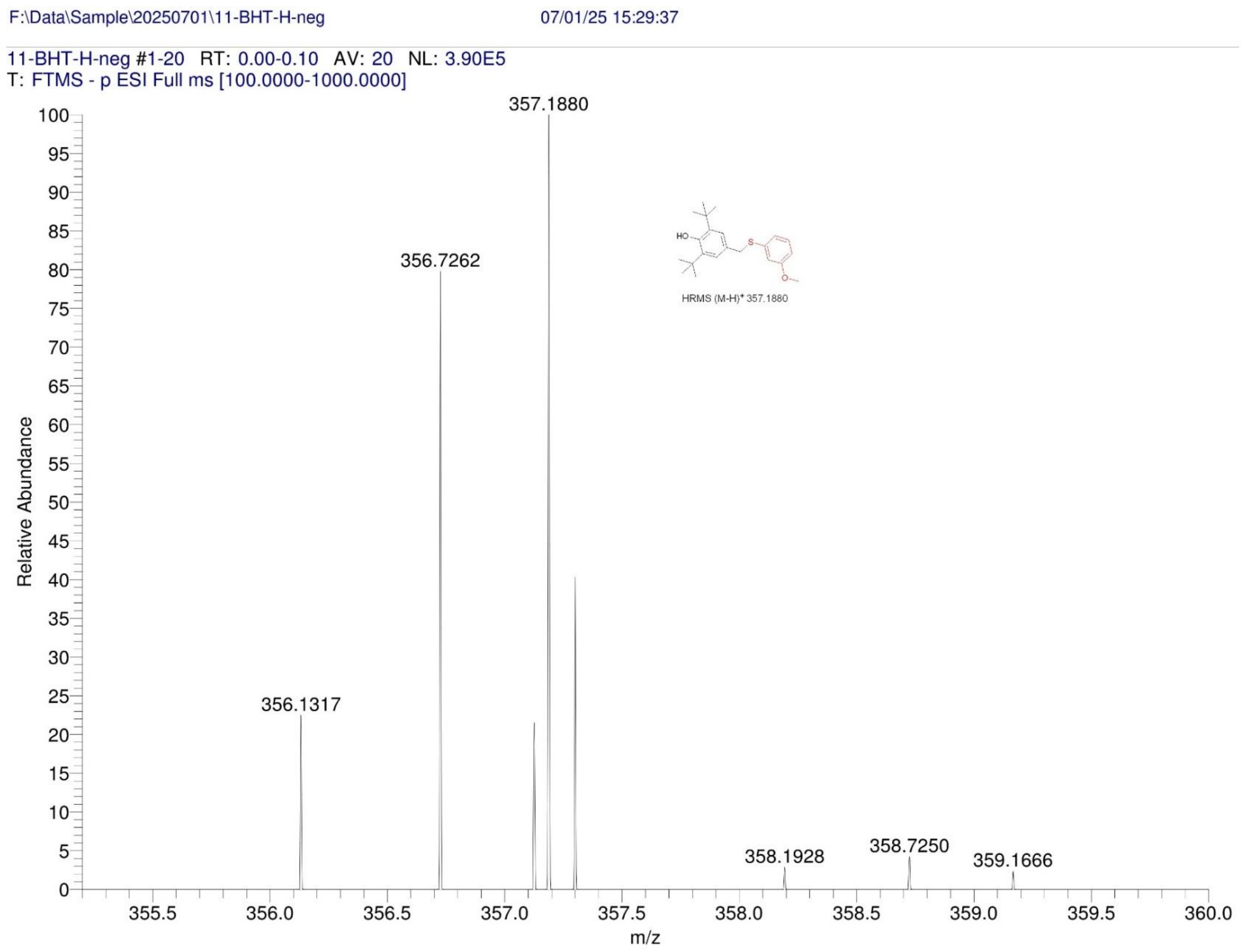

Figure S6: HRMS spectrum of the 3-methoxybenzenethiyl radical/BHT adduct
